# Genome-wide transcriptome and physiological analyses provide new insights into peanut drought response mechanisms

**DOI:** 10.1038/s41598-020-60187-z

**Published:** 2020-03-05

**Authors:** Sailaja Bhogireddy, Abishek Xavier, Vanika Garg, Nancy Layland, Renee Arias, Paxton Payton, Spurthi N. Nayak, Manish K. Pandey, Naveen Puppala, Rajeev K. Varshney

**Affiliations:** 1 International Crops Research Institute for the Semi-Arid Tropics (ICRISAT), Hyderabad, India; 2 New Mexico State University, Agricultural Science Center at Clovis, New Mexico, USA; 3 USDA FSIS OFO, Dallas, USA; 4 USDA-ARS, National Peanut Research Laboratory, Dawson, USA; 5 USDA-ARS, Cropping Systems Research Laboratory, Lubbock, USA; 6 University of Agricultural Sciences, Dharwad, India

**Keywords:** Plant stress responses, Abiotic

## Abstract

Drought is one of the main constraints in peanut production in West Texas and eastern New Mexico regions due to the depletion of groundwater. A multi-seasonal phenotypic analysis of 10 peanut genotypes revealed C76-16 (C-76) and Valencia-C (Val-C) as the best and poor performers under deficit irrigation (DI) in West Texas, respectively. In order to decipher transcriptome changes under DI, RNA-seq was performed in C-76 and Val-C. Approximately 369 million raw reads were generated from 12 different libraries of two genotypes subjected to fully irrigated (FI) and DI conditions, of which ~329 million (90.2%) filtered reads were mapped to the diploid ancestors of peanut. The transcriptome analysis detected 4,508 differentially expressed genes (DEGs), 1554 genes encoding transcription factors (TFs) and a total of 514 single nucleotide polymorphisms (SNPs) among the identified DEGs. The comparative analysis between the two genotypes revealed higher and integral tolerance in C-76 through activation of key genes involved in ABA and sucrose metabolic pathways. Interestingly, one SNP from the gene coding F-box protein (*Araip*.*3WN1Q*) and another SNP from gene coding for the lipid transfer protein (*Aradu*.*03ENG*) showed polymorphism in selected contrasting genotypes. These SNPs after further validation may be useful for performing early generation selection for selecting drought-responsive genotypes.

## Introduction

Peanut or groundnut (*Arachis hypogaea* L.) is the second most important legume in the world, generally grown as a rain-fed crop, ranking next to soybean in production. The United States is the third largest peanut producer, after China and India (FAO, 2018) and the highest peanut producing state is Georgia followed by Texas. In terms of acreage, among the four commercial types of peanut, the runner type is the most cultivated crop with 80% coverage, followed by Virginia (15%), Spanish (4%), and Valencia (1%) in the United States. Being a rainfed crop, peanut experiences drought stress in the cultivated regions of United States, including Texas, with an estimated annual loss of approximately $520 million^1,2^. The relatively low yield in the West Texas and eastern New Mexico regions may be due to the inability to provide sufficient irrigation by farmers to supplement the low rainfall received during the season. The flowering and pegging stages of the peanut life cycle are considered to be more sensitive to water deficit stress which pose adverse impact, leading to reduced yield^3,4^. Therefore, it is essential to breed improved varieties that can produce higher yield under water deficit conditions.

Being sessile, plants employ different pathway mechanisms to cope with different types of stressful environments. In particular, drought stress affects the plant’s architecture by triggering a wide range of physio-biochemical processes^5^. Plants adopt diverse strategies to combat drought stress like by reducing the stomatal conductance, decreased photosynthetic rate, accumulation of different osmoprotectants, activation of stress responsive genes and transcription factors etc.^6,7^. The level of stress tolerance in agricultural crops is generally evaluated based on the considerable loss of yield with respect to optimal growing conditions. The differences in the type of physiological response facilitate the plant either to be tolerant or sensitive towards the stress conditions. The yield of peanut among different genotypes under drought stress was screened to assess the variation of tolerance^8,9^. Different studies have reported drought-tolerant peanut genotypes, however, it is essential to check the performance of these genotypes in target locations such as West Texas and eastern New Mexico regions which faces frequent problem of limited water resources.

In West Texas and eastern New Mexico, the most widely cultivated Valencia-type peanut cultivar, Valencia-C (Val-C), is considered as standard reference, and an industrial standard variety that has been used as a control to compare yields and flavor of newly released varieties. Because of the gradual depletion of groundwater levels in these regions due to climate change, and Val-C being a low-yielding variety, it is necessary to screen different genotypes of peanuts to provide better yields for the West Texas region^10^. In peanut, related to drought there are very limited reports regarding the identification of candidate genes/genomic regions^11–13^ and transcriptome studies^14,15^. But none of these studies explored the drought tolerance mechanisms in Valencia-type peanuts; so a correct understanding of its underlying genomics will facilitate the genetic improvement of Valencia peanuts. Therefore, this study reports the identification of drought tolerance sources in the genetic background of Valencia-type peanuts. Furthermore, in recent years, the RNA-seq approach has been successfully used to understand the mechanisms behind various biotic and abiotic stresses in different crop species^16,17^. The availability of genome sequence information of diploid progenitors of cultivated peanut^18,19^ facilitates to understand the genomic complexity of the different traits in peanut. Therefore, the current study was aimed to identify the candidate genes and their molecular mechanisms involved in drought tolerance using RNA-seq approach. The results obtained from this study provide a better understanding of the tolerance mechanisms and the potential candidate genes can be utilized in breeding programs.

## Results

### Genotypic variation for pod yield and physiological features

In order to study the phenotypic variations and its associated physiological responses for pod yield among the 10 peanut genotypes (Supplementary Table S1), field level experiments were conducted in three consecutive years from 2013–2015 by imposing deficit irrigation (DI) stress. Adequate irrigation (100%) was based on the farmer’s well capacity, while deficit irrigation was achieved by reducing irrigation use by 50%. In the field experiments in different seasons, the observed precipitation varied greatly (Supplementary Fig. S1). The annual total precipitation and precipitation intervals also varied significantly. However, the average maximum and minimum temperatures were significantly consistent (Supplementary Figs. S2 and S3). Further, relative humidity and evapotranspiration rates were also recorded (Supplementary Fig. S4). Compared to other years, this result was consistent with the least rainfall in 2013. In addition, soil moisture content was also collected, where fully irrigated soil was very moist at 48 cm for most of the growing season and at 12, 24 cm, the soil moisture had a smaller interval of dampness and dryness due to irrigation and precipitation events (Supplementary Figs. S5 and S6).

Based on the phenotyping results from the annual analysis of variance, the irrigation treatment (T) was significant in the years 2013 and 2014 (*P*-value <0.0001) and in 2015 (*P*-value <0.01)). These results indicate that each year the yield was significantly different depending on the T, genotype (G) and T × G (Supplementary Table S2). In 2013, the fully irrigated (FI) plot produced about 2828 kg/ha^−1^, while the deficit irrigated plot generated about 1879 kg/ha^−1^, a reduction of about 34% compared to the fully irrigated plot. Further, yield reduction in the deficit-irrigated plots was about 67% and 13% in 2014 and 2015, respectively (Supplementary Table S3). Overall, in three years, the best genotypes obtained under full irrigation conditions in West Texas were Tamspan-90, ICGS 76 and C76-16 (C-76) and poor yielders were Valencia-C (Val-C), ICGV 86388 and TMV-2. On the other hand, under the conditions of water deficit, C-76 showed better response accounted by its mean yield 3278 kg ha^−1^, higher among the other genotypes followed by Tamspan-90, ICGS 76, COC-041, ICGV 86051 and Val-C (Table 1).

**Table 1:** Yield (Kg ha^-1^) of different peanut genotypes under contrasting irrigation treatments during 2013–2015.

	Genotype	2013		2014		2015		Average	
		Full irrigation	Deficit irrigation	Full irrigation	Deficit irrigation	Full irrigation	Deficit irrigation	Full irrigation	Deficit irrigation
1	ICGS 76	3667	2410	5719	1423	4978	3777	4788	2537
2	C76-16	3856	3827	4761	1715	5431	4292	4683	3278
3	COC041	3244	1673	4279	1246	3623	4354	3715	2424
4	ICGV 86051	2652	1667	4537	1818	4412	3574	3867	2353
5	Serenut-5R	3085	1379	2972	1194	3636	2965	3231	1846
6	Serenut-6T	2808	1513	4856	1478	4011	3075	3892	2022
7	TMV-2	2882	1719	4273	1075	3512	3512	3556	2102
8	ICGV 86388	1487	873	4738	972	3043	3168	3090	1671
9	Tamspan-90	3472	2598	4951	2083	4838	3621	4420	2767
10	Valencia-C	1131	1131	3982	1692	3824	3792	2979	2205
	**Mean**	**2828**	**1879**	**4507**	**1469**	**4131**	**3613**	**3822**	**2320**
	**LSD**	**487.78**		**827.32**		**886.45**		**748.53**	

**Figure 1:**
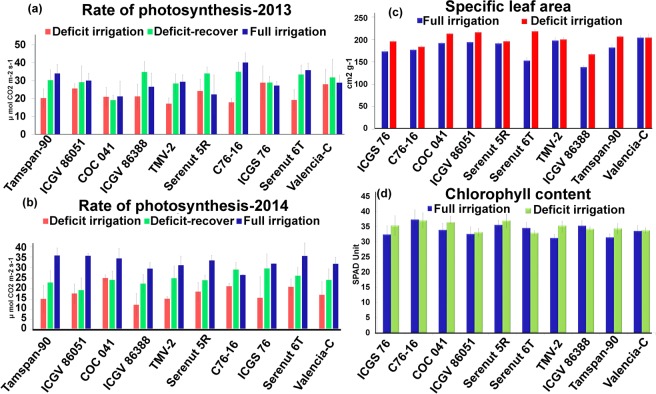
Physiological observations from fully irrigated and deficit irrigation plots at different time intervals: (**a,b**) Mean gas exchange measurements in the years 2013 and 2014. (**c**) Mean specific leaf area (SLA) during irrigated and deficit irrigation conditions in selected genotypes. (**d**) Mean chlorophyll content during irrigated and deficit irrigation conditions in selected genotypes.

Net photosynthesis at the leaf level was measured during the water deficit treatment to monitor the impact of deficit irrigation (DI) stress on the plant metabolism. In 2013, the water deficit treatment resulted in a moderate decrease in net photosynthesis for most of the genotypes, but no significant differences were observed during stress, recovery, or complete irrigation among the genotypes. In 2014, treatment resulted in a significant decrease in net photosynthesis (ranging from 18 to 38 µmolesm^−2^s^−1^) compared to the full irrigated treatment (ranging from 20 to 40 µmolesm^−2^s^−1^) but again no statistically significant differences could be observed between the genotypes. General trends emerged in both the years for photosynthetic responses, but in this case, net photosynthesis was used to guide the collection of leaf material for transcriptome studies (Fig. 1a,b). Further, SLA and chlorophyll content were also measured in 2014 but the values obtained were not statistically significant between the treatments of the same genotypes (Fig. 1c,d).

### Sequencing and mapping statistics using reference guided assembly

Based on the observed phenotypic variations from our previous^9^ and present drought studies, two genotypes- C76-16 (C-76, runner type) and Valencia-C (Val-C, Valencia type) were identified as the contrasting genotypes towards drought response and thus selected for transcriptome studies. Leaf samples for the RNA-seq were collected from C-76 and Val-C genotypes in 2014 in three biological replicates for transcriptome profiling and generated ~369 million raw reads. The raw reads were filtered to obtain high-quality reads which were then further used in the downstream analysis and for mapping on A (*A*. *duranensis*) and B (*A*. *ipaensis*) diploid ancestor genomes^18^ of peanut separately.

**Figure 2:**
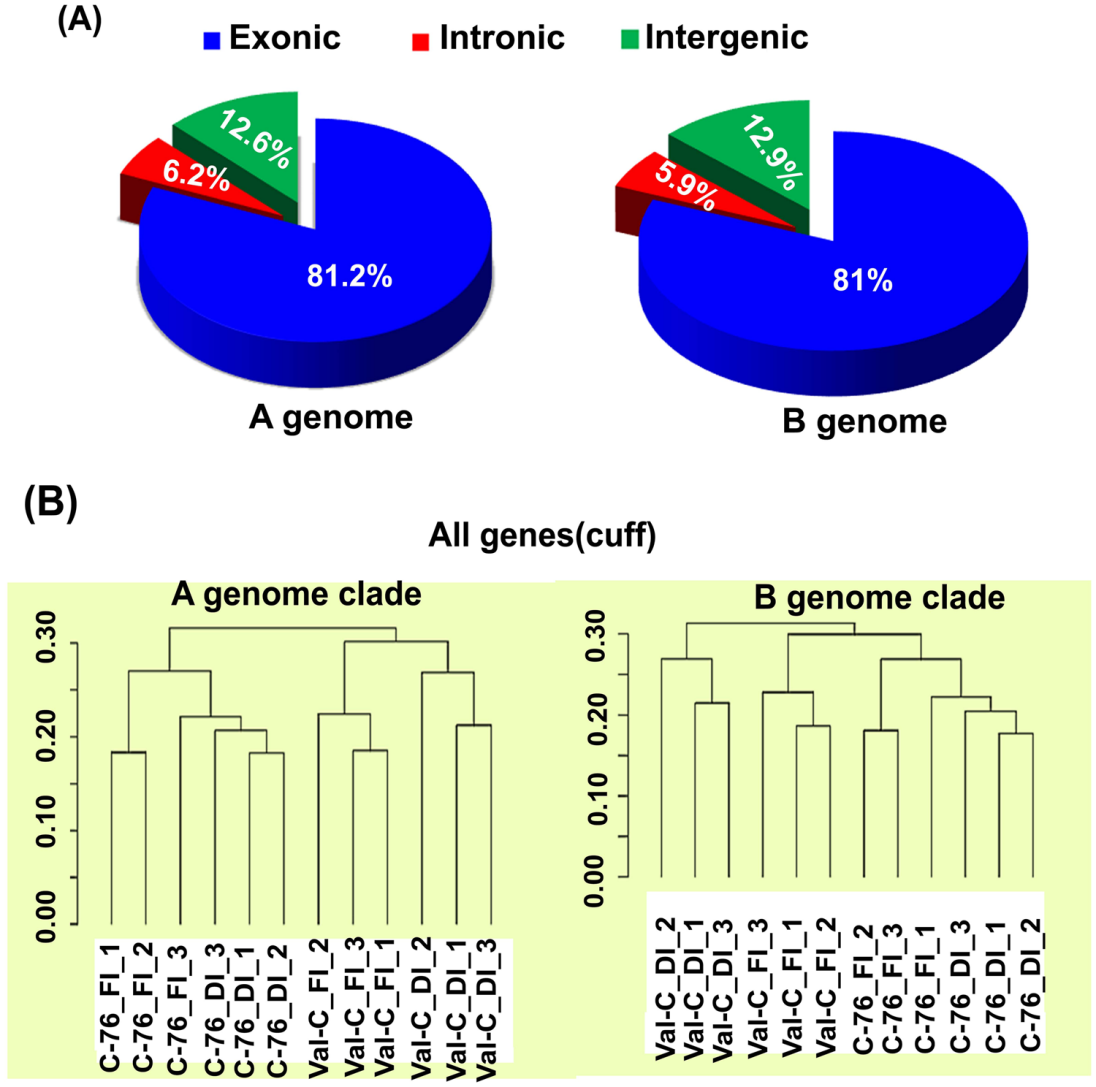
Mapping statistics and dendrogram showing correlation among the different samples: (**A**) An overview of the mapped reads on (**A,B**) genomes of *Arachis* progenitors**:** The distribution of fragments represented as the percentage of reads that map onto exons, introns and intergenic regions on A and B genomes of peanut (**B**) Dendrogram constructed based on the expression values in fully irrigated (FI) and deficit irrigation (DI) samples in two different genotypes- C-76 and Val-C with biological replicates on two genome clades.

A total of 329.2 million (90.2%) and 329.4 million (90.3%) filtered reads were mapped to A and B genomes, respectively. In C-76, on an average, 30 and 26 million reads per replicate were mapped under FI and DI conditions, respectively on A and B genomes. Whereas in Val-C genotype, it was observed that approximately ~25 and ~27 million reads per replicate were mapped under FI and DI conditions, respectively on A and B genomes (Table 2). About 81.2% of the filtered reads were mapped to the exonic regions of the A and B genomes, while ~6% were mapped on the intronic region, and ~12.8% were mapped on the intergenic regions (Supplementary Table S4 and Fig. 2A). Expression analysis was performed for the four samples, namely C-76_FI, C-76_DI, Val-C_FI and Val-C_DI. The level of gene expression was calculated in the four samples using fragments per kilobase of transcript per million mapped reads (FPKM) values. A total of 28,524 and 29,490 genes were observed to be expressed on A and B genomes, respectively. Further, in dendrogram constructed based on expression values, individual replicates of C-76 and Val-C were falling in two different groups, one belonging to the Val-C group and the other to the C-76 group consisting of their respective FI and DI samples. In A genome, the replicates of C-76 and Val-C genotypes were grouped separately. Similarly, for the two conditions- FI and DI, the replicates were also grouped separately under their respective genotypes (Fig. 2B). However, for the B genome, there is a little deviation from the A genome, where the irrigated samples of Val-C were falling under the C-76 but these were not grouped together (Fig. 2B).

**Table 2:** Summary of the RNA-seq data generated under FI and DI conditions.

Sample label	Raw reads generated (millions)	High-quality reads (millions)	Total reads mapped on A genome (millions)	Total reads mapped on B genome (millions)
C-76 -FI-1	28.18	27.79	25.13	25.18
C-76 -FI-2	37.24	36.68	33.38	33.32
C-76 -FI-3	38.35	37.73	34.04	34.03
C-76 -DI-1	31.66	31.26	28.10	28.19
C-76 -DI-2	28.14	27.75	25.19	25.25
C-76 -DI-3	27.83	27.42	24.86	24.83
Val-C-FI-1	30.03	29.59	26.73	26.81
Val-C-FI-2	28.86	28.41	25.44	25.49
Val-C-FI-3	27.65	27.26	24.73	24.78
Val-C_DI-1	30.82	30.41	27.16	27.16
Val-C-DI-2	32.49	32.07	28.99	28.96
Val-C-DI-3	28.55	28.19	25.40	25.4
**Total**	**369.8**	**364.6**	**329.2**	**329.4**

*FI-Full irrigation; DI-Deficit irrigation.

### Differential gene expression analysis

**Figure 3:**
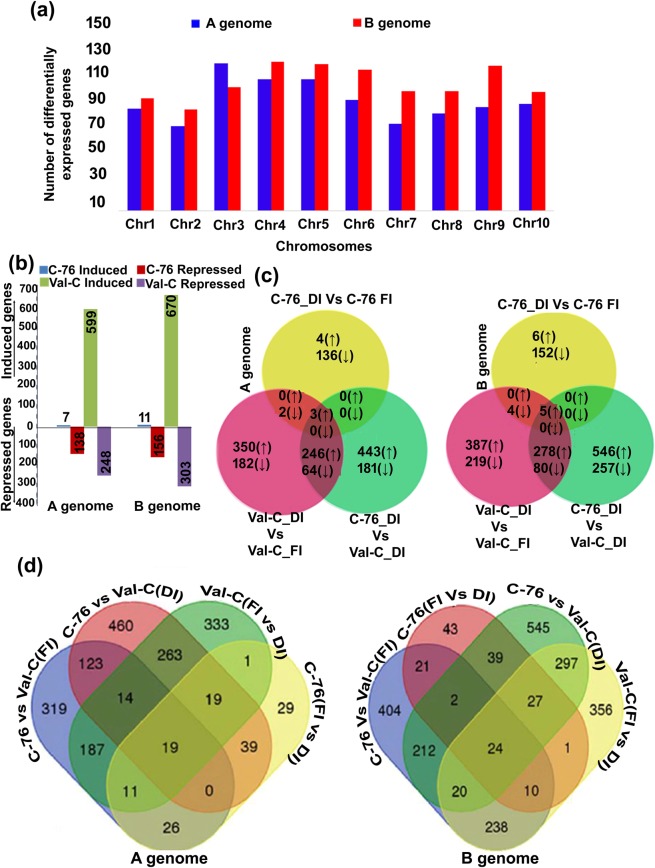
Differential gene expression analysis in C-76 and Val-C genotypes during deficit irrigation conditions: (**a**) Number of differentially expressed genes (DEGs) during deficit irrigation (DI) across 10 pseudomolecules on A and B genomes (**b**) Bar graph representing number of induced/upregulated and repressed/downregulated genes in C-76 and Val-C during DI with respect to fully irrigated (FI) conditions (numbers on the bars representing the number of up and downregulated genes) (**c**) Venn diagram representing the common and unique upregulated and down-regulated genes during deficit irrigation on A and B genomes of C-76 and Val-C (**d**) Venn diagram depicting the common and specific DEGs in different combinations of C-76 and Val-C genotypes during DI and FI on A and B genomes of peanut.

Differential gene expression analysis revealed 2,037 and 2,471 differentially expressed genes for C-76 and Val-C genotypes, respectively on comparison of their corresponding DI samples with respect to their FI samples. Genes with a *P*-value ≤ 0.05 and $|lo{g}_{2}(fold\,change)|\ge 2$ were considered as differentially expressed genes (DEGs). The identified DEGs were studied for their distribution on the pseudomolecules of A and B genomes. The highest number of DEGs were present on pseudomolecules A03 (118) and B04 (119) while the minimum were observed on pseudomolecules A02 and B02 (Fig. 3a). The number of up and downregulated genes in C-76 and Val-C during DI with respect to FI on A and B genomes were identified (Fig. 3b). Differentially expressed genes in three combinations (C-76_DI vs C-76_FI; Val-C_DI vs Val-C_FI; C-76_DI vs Val-C_DI) revealed that more DEGs were upregulated in Val-C (599) than C-76 (7) during DI compared to FI and 3 DEGs were found in common on A genome. Similarly, the number of downregulated genes were 248 in the case of Val-C and 138 in C-76 during DI compared to FI, and 2 genes were common in both the genotypes on A genome and similar is the case with the B genome (Fig. 3c). In addition, the DEGs of four different combinations [C-76 vs Val-C (FI); C-76 vs Val-C(DI); C-76 (FI vs DI); Val-C(FI vs DI)] were compared to identify common and unique DEGs (Fig. 3d), where 19 (A genome) and 24 (B genome) DEGs were common across all four comparisons (Supplementary Table S5; Fig. 3d).

### Differentially expressed genes during deficit irrigation (DI)

**Figure 4:**
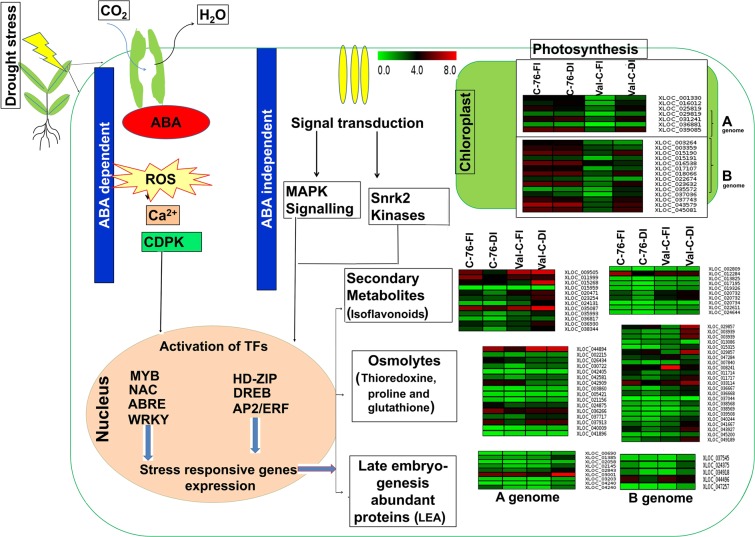
An overview of differentially expressed genes in drought stress-responsive pathway mechanism: Heat maps demonstrating the expression profiles of differentially expressed drought stress-responsive genes during fully irrigated (FI) and deficit irrigation (DI). Color scale represents normalized FPKM values. Corresponding reference gene ids (with suffix XLOC) falling have been given on the right side. The color scale on the top represents normalized FPKM values.

Differential expression analysis in DI revealed the expression of drought stress-responsive genes in the two selected genotypes. Interestingly, the common DEGs (19 on A genome) showed fairly contrasting expression in C-76 and Val-C (Fig. 3d; Supplementary Fig. S7). The common DEGs encode for *Gcn*5*-related N-acetyltransferase* (GNAT) gene, *BON1- associated protein*, the *lateral organ boundary (LOB)*, and the *late embryogenesis abundance* (*LEA*) genes etc., (Fig. 3d and Supplementary Table S5). Especially, genes related to osmoprotectants, photosynthesis, abscisic acid, secondary metabolites and other gene families that are responsive during drought stress showed distinct expression patterns in C-76 and Val-C (Fig. 4).

In C-76 under DI with respect to FI, the induced expression of plant protease inhibitor genes such as *trypsin inhibitors* and *cystatin*, as well as the *ATP- binding cassette* (*ABC*) *transporter* and the *lectin precursor* genes and *LOB* was noted. While in Val-C, induced DEGs include genes related to cell wall and membrane-bound genes such as *ureide permease 2*, *expansin A10*, *xyloglucan endotransglucosylase hydrolase*, and *expansin-A8* when compared DI *versus* FI. On the other hand, highly upregulated genes include the genes encoding *cysteine endopeptidase*, *asparticase oryzasin-1* and *ubiquitin-E3ligase* in Val-C. Similarly, other drought-responsive DEGs are *aquaporin PIP-2*, *abscisic acid (ABA) insensitive 5*, *ethylene-responsive transcription factor*, *expansin 2*, *L-ascorbate oxidase*, *LEA*, *peroxidase*, *glutathione S-transferase*, *thioredoxin reductase* and *trehalose-phosphate phosphatase*, heat shock proteins (HSP) encoding genes such as *Dnaj/HSP40*, *Class II HSPs* and *heat shock factor* (*Hsf*)30 and genes involved in secondary metabolism like isoflavonoid and flavonoid synthesis, were induced under DI in Val-C with respect to DI in C-76 (Supplementary Table S6).

When FI samples from two genotypes were compared, expression of genes for drought stress response pathways such as photosynthesis-related genes-*accumulation of photosystem one1* (*APO1*) and photosystem II family protein-coding genes (D2 protein), osmoprotectant genes like *abscisic acid 8-hydroxylase*, *proline imino-peptidase*, *peroxidase* genes and other genes encoding for *ethylene-responsive transcription factor* (ERF) and *lipid transfer protein* genes showed high expression levels in C-76, indicating its higher level of tolerance (Supplementary Table S7).

### Gene ontology

**Figure 5:**
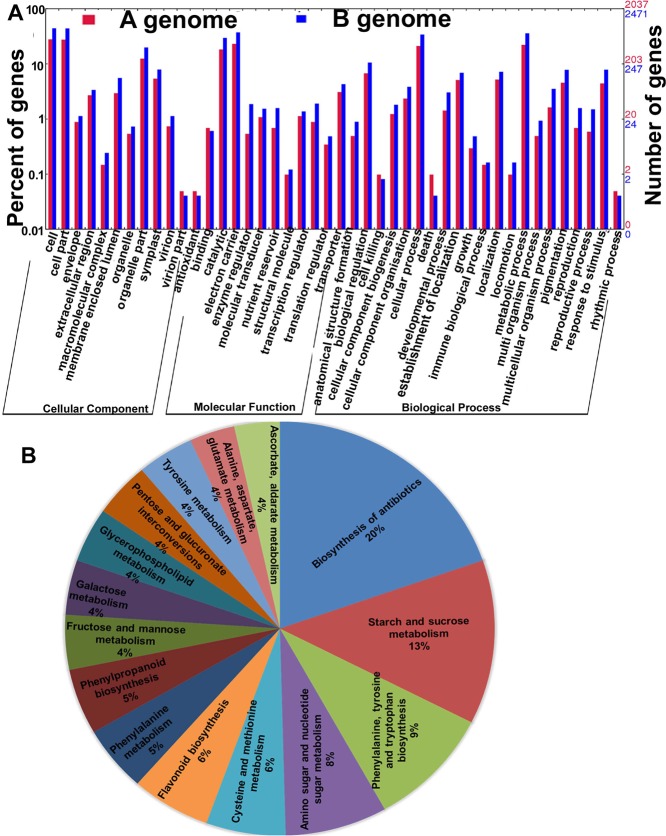
Gene ontology and pathway analysis: (**A**) Functional categorization of drought-responsive genes under different gene ontology (GO) categories-cellular component, biological process and molecular function. (**B**) The top 15 different pathways involved in deficit irrigation stress of C-76 and Val-C genotypes including the representation of percent of genes involved in each pathway.

The gene ontology of DEGs revealed their functional annotation by categorizing them into cellular, molecular, and biological components (Fig. 5A). The majority of genes have been annotated on biological processes followed by cellular component and molecular function. In total, 38.7% (A genome) and 38.0% (B genome) genes were falling under 12 categories of cellular components while 37.66% (A genome) and 38.2% (B genome) genes were falling under 21 categories of biological process, 23.7% (A genome) and 23.8% (B genome) genes were falling in molecular function under 11 categories.

### Pathways associated with deficit irrigation (DI)

Differentially expressed genes were analyzed to study their association with different metabolic pathways. The significant role was noted for 104 pathways representing biosynthesis, metabolism and degradation process. Pathways related to the biosynthesis of antibiotics (23 genes), starch and sucrose metabolism (15), biosynthesis of aromatic amino acids (11), metabolism of amino and nucleotide sugars (9), metabolism of sulfur amino acids (7), biosynthesis of flavonoids (7), etc., showed an association with DEGs (Fig. 5B and Supplementary Table S8). Different genes in the antibiotic synthesis pathway encoding for *bifunctional aspartate aminotransferase*, *aspartate prephenate-aminotransferase*, *glyceraldehyde-3-phosphate dehydrogenase*, *pyrophosphate-fructose 6-phosphate 1-phosphotransferase*, *acetyl coenzyme A*, *3-dehydroquinate dehydratase shikimat*e, *methylecgonone reductase*, *chorismate mutase*, etc. were found to be expressed (Supplementary Table S9). Similarly, genes related to starch and sucrose metabolic pathway include, *endo-1*,*3-beta-glucosidase*, *alpha-amylase*, *glucomannan 4-beta-mannosyltransferase 2*, *beta lysosomal glucosidase*, *hexokinase*, *endoglucanase 6* and *cellulose synthase*, etc., which were found differentially expressed. Secondary metabolic pathways such as flavonoids and alkaloids were also found to be affected by drought stress. In both genotypes, the amino acid and nucleotide pathways followed by the carbohydrate and lipid metabolic pathways were found associated with water deficit stress.

### Identification of transcription factors (TFs) during deficit irrigation

**Figure 6:**
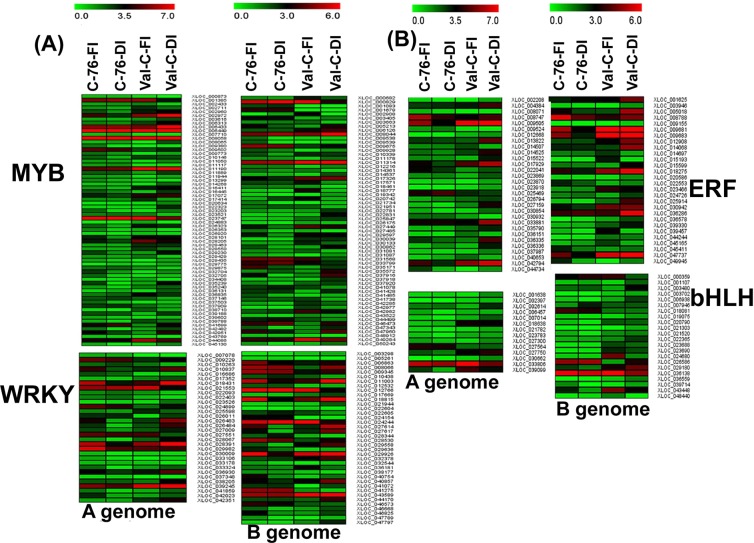
Heat maps representing the expression profiles of highly expressed transcription factor (TF) gene families: Expression of genes encoding (**A**) MYB and WRKY (**B**) ERF and bHLH TFs during fully irrigated (FI) and deficit irrigation (DI) conditions of C-76 and Val-C genotypes on A and B genomes of peanut. Corresponding reference gene ids (with suffix XLOC) falling under each TF family have been given on the right side. The color scale on the top represents normalized FPKM values.

 The differential expression of genes encoding transcription factors (TFs) were identified in the peanut under DI. A total of 715 and 839 TF genes belonging to 58 classes were differentially expressed in the peanut A and B genomes, respectively. Among the differentially expressed TF encoding genes, the highest number of genes were from MYB family for both A and B genomes followed by WRKY, and ERF during DI (Supplementary Fig. S8). The MYB, bHLH, WRKY, ERF, NAC and C2H2 transcription factor family encoding genes were abundantly expressed in both genotypes during the DI conditions (Fig. 6A,B). In addition, the analysis revealed that ABC-2 type transporter and LBD (LATERAL ORGAN BOUNDARY DOMAIN) TF were found to be upregulated during DI conditions with respect to FI in C-76. Similarly, in Val-C during DI *versus* FI, the ABC transporter, other TF genes, such as NAC, the bHLH family, cytochrome P-450, WRKY, and NF-Y, were found to be induced.

### Validation of DEGs from RNA-seq using quantitative real-time PCR (qRT-PCR)

**Figure 7:**
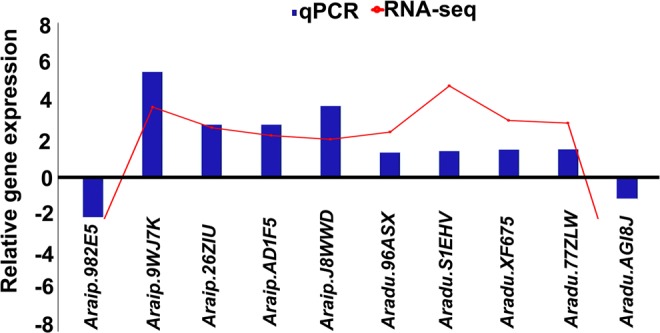
Validation of RNA-seq results using quantitative real-time PCR (qRT-PCR): Expression correlation of genes using qPCR and RNA-seq data. Validation of gene expression patterns of 10 genes during deficit irrigation (DI) in comparison with their respective fully irrigated (FI) samples. The y-axis represents the relative gene expression during DI *versus* FI.

The expression of RNA-seq was also validated by qRT-PCR. For this study, a total of 10 DEGs, five genes each from A genome and B genome were selected and primers were designed (Supplementary Table S10). These include- *ubiquitin- ligase*, *NHL domain-containing*, *peptidyl- prolyl cis-trans isomerase*, *LRR receptor serine- threonine kinase*, *vinorine synthase*, *LEA*, *DNA mismatch repair MSH6*, *ATPase alpha partial*, *laccase-14* and *plastid movement impaired 2*. Although there was a small variation in the degree of expression, almost all of the 10 genes selected for validation showed the same pattern of expression as displayed in RNA-seq data (Fig. 7) confirming the results achieved through RNA-seq analysis.

### SNP marker identification and validation

**Figure 8:**
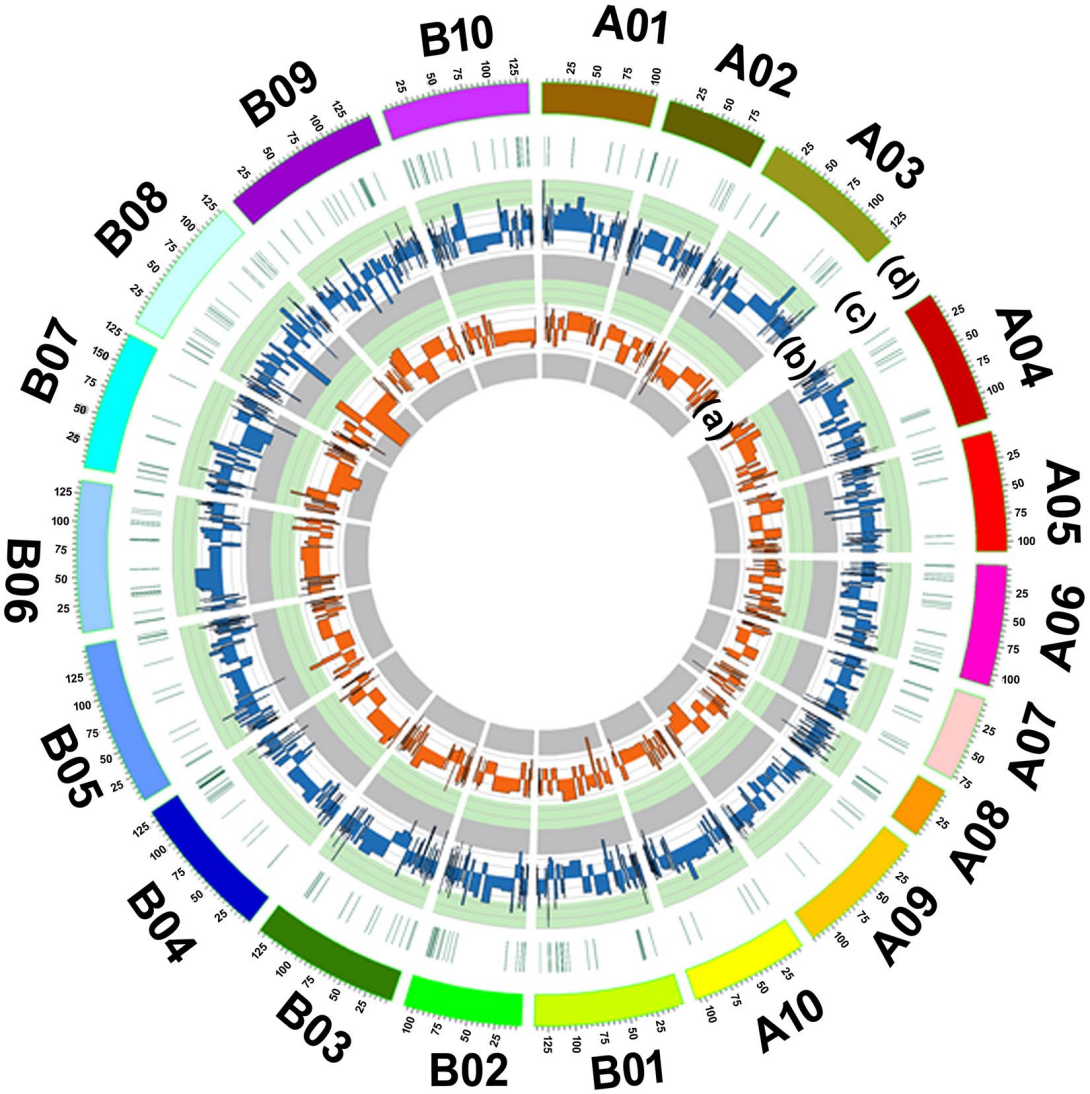
Circos representing the distribution of transcription factors (TFs), differentially expressed genes (DEGs) and SNPs across the different pseudomolecules of reference genomes of peanut: Concentric circles in the circos plot from inside to outside (**a**) orange color representing the expression profiles of transcription factors (**b**) blue color representing differentially expressed genes (DEGs) (**c**) green color representing the SNP density and (**d**) outer most track (in multicolor) representing pseudomolecules of peanut on two genomes of A and B.

A total of 514 SNPs were identified between C-76 and Val-C, occurring in DEGs when aligned with A (190) and B (324) genomes. The identified SNPs were present on 270 DEGs collectively, where 95 genes were present on the A genome and 175 genes on the B genome. The SNPs were identified in four different combinations and were divided into missense, 3′UTR, 5′UTR, splice region, stop gained, start lost and stop loss variants (Table 3). The distribution of SNP variants in pseudomolecules on different DEGs has been shown in Fig. 8. Several missense variants on A and B genomes in four combinations were 116 and 225, respectively. A total of 23 sets of allelic specific primers were designed for 23 genes to validate polymorphism in C-76 and Val-C (Supplementary Table S11) using qRT-PCR. Homozygous and heterozygous alleles were detected based on the variations obtained in the cycle threshold (Ct) values. If the Ct value is <30.0 for the set of allelic primers of one gene in one genotype but not in the other, this is considered to be the presence of two different alleles and vice versa. Of the 23 allelic primers designed, 21 sets of allelic primers did not show any polymorphism (Table 4), while two SNPs representing two genes exhibited polymorphism. The first SNP (Ct > 30 in C-76 and Ct <30 in Val-C) was from the gene coding the F-box protein (*Araip*.*3WN1Q*) while another SNP (Ct value of <20 in C-76 and Ct> 25.0 in Val-C) was from the gene coded for the lipid transfer protein (*Aradu*.*03ENG*). These validated SNPs from the genes *Araip*.*3WN1Q* and *Aradu*.*03ENG* can be used for performing early generation screening of the breeding material arising from crosses involving source parent for good performance under water deficit stress.

**Table 3:** SNPs distribution in different combinations across A and B genomes of peanut.

Genome	SNP type	C-76- FI Vs Val-C-FI	C-76-FI Vs C-76 -DI	Val-C-FI Vs Val-C-DI	C-76-DI Vs Val-C-DI	Total
**A**	3‘UTR’ variant	17	0	13	22	52
	5,‘UTR’ variant	11	0	4	5	20
	Missense variant	43	1	21	51	116
	Stop gained	1	0	0	1	2
**B**	3‘UTR’ variant	15	0	8	24	47
	5‘UTR’ variant	13	0	17	15	45
	Missense variant	81	1	55	84	221
	Splice region variant	1	0	0	0	1
	Stop gained	3	0	1	4	8
	Start lost	0	0	0	1	1

**Table 4:** SNPs validation using qRT-PCR.

S.No	Gene Id	Locus	Allele	Primer set	C-76	Val-C
1	*Araip*.*1ZJ86*	Araip.B05:9584285–9586662	T/A	Allele1	+	+
				Allele2	−	−
2	*Araip*.*XI9UR*	Araip.B01:121358660–121360450	C/G	Allele1	−	−
				Allele2	+	+
3	*Araip*.*L4UC8*	Araip.B04:128351613-128357097	G/A	Allele1	−	−
				Allele2	+	+
4	*Araip*.*J8WWD*	Araip.B05:149331140-149333249	C/G	Allele1	−	−
				Allele2	+	+
5	*Araip*.*AD1F5*	Araip.B05:149331140-149333249	T/G	Allele1	−	−
				Allele2	+	+
6	*Araip*.*9WJ7K*	Araip.B07:7841693-7846317	A/T	Allele1	−	−
				Allele2	+	+
7	*Aradu*.*XF675*	Araip.B07:7841693-7846317	G/A	Allele1	−	−
				Allele2	+	+
8	*Aradu*.*03ENGI (1 & 2)*	Aradu.A02:84440367-84441960	T/A	Allele1	++	+
				Allele2	−	−
9	*Aradu*.*03ENG II(3 & 4)*	Aradu.A02:84440367-84441960	T/A	Allelle1	−	−
				Allele2	+	+
10	*Araip*.*G4DKZ*	Araip.B03:109942672	C/T	Allele1	−	−
				Allele2	+	+
11	*Aradu*.*77ZLW*	Aradu.A10:5944901-5947659	C/T	Allele1	+	+
				Allele2	−	−
12	*Araip*.*3WN1Q*	Araip.B01:23319799-23321171	A/T	Allele1	−	+
				Allele2	−	−
13	*Araip*.*26ZIU*	Araip.B01:123879553-123882176	T/A	Allele1	+	+
				Allele2	−	−
14	*Araip*.*6FC6W*	Araip.B09:115020963-115024147	G/T	Allele1	+	+
				Allele2	−	−
15	*Araip*.*982E5*	Araip.B06:1022422-1025983	T/G	Allele1	−	−
				Allele2	+	+
16	*Aradu*.*96ASX*	Aradu.A02:1911658-1916520	T/A	Allele1	−	−
				Allele2	+	+
17	*Aradu*.*CVI2N*	Aradu.A03:28240-34249	A/G	Allele1	−	−
				Allele2	+	+
18	*Aradu*.*618YE*	Aradu.A10:95832914-95833751	A/C	Allele1	−	−
				Allele2	+	+
19	*Aradu*.*S1EHV*	Aradu.A04:30101833-30104322	G/T	Allele1	+	+
				Allele2	−	−
20	*Aradu*.*AGI8J*	Aradu.A08:34838458-34855099	G/A	Allele1	+	+
				Allele2	−	−
21	*Aradu*.*R479P*	Aradu.A05:18360103	A/G	Allele1	−	−
				Allele2	+	+
22	*Araip*.*JH8FI*	Araip.B04:127200939-127205614	C/G	Allele1	+	+
				Allele2	−	−
23	*Araip*.*JH8FI*	Araip.B04:127200939-127205614	C/G	Allele1	−	−
				Allele2	+	+

*** “+” CT Value <30.0; “++” CT Value <20.

## Discussion

Cultivated peanut is an important legume and comprises of allotetraploid genome (type AABB; 2n = 4x = 40) with a total size of about 2.7GB. The availability of genome sequence information of the diploid progenitors (*A*. *duranensis* and *A*. *ipaensis*) of cultivated peanuts^18,19^ allows us to study the genome wide transcriptome variations in different peanut lines. More recently, the high-quality reference genomes for both the subspecies of cultivated peanuts have become available (was not available at time of this study), which will further enhance the precision of future genomics and transcriptomics studies in peanut^20–22^. This study is a combined approach to physiological and transcriptomic responses of peanut genotypes or breeding material under water deficit conditions. Yield and physiological attributes were measured in 10 selected peanut genotypes and further RNA-seq was performed in two different genotypes to study the transcriptome changes that occur during deficit irrigation (DI) and fully irrigated (FI) conditions. In addition, this is a unique study, where the samples from our study were collected in the field to facilitate natural environmental conditions rather than using growth chambers for controlled conditions.

Among the ten different genotypes selected for this study C-76-16 (C-76) showed better performance in terms of mean yield under DI conditions. While on the other hand, check cultivar Valencia-C (Val-C) showed lesser yield. These results are well synchronizing with our earlier study on the pod yield performance of different peanut genotypes under different soil and regional conditions^9^, where C-76 produced more than Val-C in rainfed conditions without irrigation but there is a considerable reduction in C-76 yield by 15–20% compared to Val-C, which was up to 50–70% reduction in yield compared to complete irrigation^9^. Another study in peanut^23^ showed a lower yield of Valencia-type pods (PI 536121) under drought conditions. Since Val-C is considered standard control for West Texas and eastern New Mexico regions, there is a need to improve the genetic potential of Val-C in terms of yield in case of water deficit conditions. This facilitates the realization of transcriptomic study on genotypes C-76 (runner type) and Val-C (Valencia type) to understand the underlying genetic mechanisms. Although the genotypes selected belong to different market classes and several factors are added, the comparison of the responses in the transcriptome of the leaf is adequate because the two genotypes represent the same species. Transcriptome analysis in C-76 and Val-C revealed the expression of 28,524 and 29,490 genes on A and B genomes of peanut, where more number of expressed genes hitting to B genome described the direct effect on the progeny of *A*. *ipaensis* from the same population that contributed to the B genome of the cultivated peanut^18^. Further, the analysis showed the expression of a very less number of DEGs in C-76 under DI *versus* FI. The number of induced genes was very less in C-76 is a rather interesting phenomenon. These results are consistent with those of the sorghum study where more DEGs were expressed in drought-sensitive than drought-tolerant subjects^24^.

Differential expression of functional and regulatory genes responsive to drought stress was induced in Val-C relative to C-76 on comparison of their corresponding DI samples with respect to their FI samples (Supplementary Table S6). This shows that C-76 showed a significant level of tolerance in DI and may not be experiencing water deficit stress, unlike Val-C. This can also be explained in the other direction, as the stress imposed was not sufficient for the C-76 to express more number of drought stress responsive genes during DI *versus* FI. However, in Val-C for the same level of imposed stress, various genes related to drought stress-responsive mechanisms/pathways were activated robustly to combat stress. Comparatively, Val-C showed the activation of a maximum number of drought-responsive pathways than C-76 during DI *versus* FI conditions, which could form the basis of the contrasting response of both genotypes. The adaptation to different environmental conditions varies according to the genotypes of solitary plant species, which can be explained by the phenomenon of natural variation^25^ and the level of tolerance to any type of stress varies from one genotype to another^26^.

It is interesting to note that the contrasting expression profiles of common DEGs, such as *trypsin inhibitors*, *late embryogenesis abundant* (*LEA*), *ABC transporter* and *LOB containing domain*, etc. in both genotypes during DI, suggest that there are significant differences between genotypes in terms of irrigation and water deficit conditions (Supplementary Fig. S7). In maize, the reciprocal expression of DEGs that responds to drought between two contrasting inbred lines, causing under water deficit stress reveals drought tolerance mechanisms^27^. In Arabidopsis a strong accumulation of *trypsin inhibitor* genes in water deficit periods was reported^28^. Similarly, LEA proteins are known to accumulate during water stress to prevent intracellular water loss. The increased expression of cellular LEA proteins shows tolerance to drought stress in sorghum^29^, and foxtail millet^30^.

Phytohormones, also play a crucial role in water deficit stress in plants. The accumulation of abscisic acid (ABA) can lead to stomatal closure, which reduces water loss and subsequently stops cell growth and eventually results in increased concentration of reactive oxygen species (ROS)^31,32^. Genes such as *abscisic acid 8 hydroxylase*, *proline imino-peptidase* and *sucrose synthase*, which play a crucial role in drought stress response, shown the decreased expression in Val-C during DI. On the other hand, the increased expression of these genes was noticed in C-76 compared to Val-C under FI. The increased abscisic acid can be catabolized in the cell by the *abscisic acid 8-hydroxylase* acid to form 8′-OH abscisic acid to maintain cellular homeostasis, has been reported in the grapevine^33^. The accumulation^33^ of ABA is negatively regulated by the plant’s mechanism through the activation of catabolic enzymes of ABA during water stress to enhance tolerance^34,35^. In the present study, the downregulation of genes related to *abscisic acid 8-hydroxylase* and *cytochrome p450* (*CYP*) genes in Val-C implies the downregulation of abscisic acid pathway under DI, which in turn leads to an accumulation of ROS and finally cell damage. In addition, reduced expression of sucrose synthase in Val-C could play an important role in yield reduction due to the disproportion of the source to sink ratio. The *sucrose synthase* activity plays a crucial role in the biosynthesis of starch and storage proteins^36^. The increased activity of sucrose synthase leads to increased grain assimilation in wheat under water stress^37^.

In addition, genes related to photosynthesis showed significant discrimination between two contrasting genotypes especially during FI conditions. The genes related to light reactions of photosynthesis of photosystem I and II were found to be downregulated in Val-C *versus* C-76 during FI conditions (Supplementary Table S12). The downregulated genes related to the subunits of photosystems I and II under drought stress conditions, reduced plant tolerance levels to drought stress in sorghum^24^. Decreased expression of genes involved in light-harvesting chlorophyll a/b-binding proteins (LHCBs) and photosystems I and II results in decreased drought tolerance in plants^38,39^. During irrigated conditions, the genes encoding *P700 apo A1* of photosystem I, and *D2 of photosystem II* exhibited comparatively high expression in C-76 than Val-C. The D2 protein of photosystem II plays a vital role in stress tolerance (Supplementary Table S12). Among the different DEGs identified, genes related to photosynthesis showed a distinct expression in both genotypes. Therefore, it is essential to comprehensively study the photosynthetic genes associated with drought stress in peanuts, which gives a better idea in the development of drought-tolerant genotypes. In addition, the expression of genes encoding different transcription factors (TFs) such as MYB, NF-Y, NAC, WRKY, ERF, bHLH represents the induction of the ABA-dependent signalling pathway as a stress response (Supplementary Fig. S8). The involvement of drought-responsive WRKY and NAC TFs may be useful in the detoxification of ROS by regulating downstream target genes^40^. In addition, the SNP validation study has provided the information on genetic markers which upon further validation in larger set of genotypes can be used in genomic-assisted breeding for enhancing drought stress tolerance.

In summary, this is a combined study of phenotypic, physiological and genome-wide transcriptome analysis in diverse peanut genotypes. The germplasm line C-76 yielded the best results among the 10 accessions tested in our study. Although demand for the high oleic trait is high, when water resources are limited, processors will contract farmers to grow a variety that can provide better performance under limited irrigation conditions. Hopefully, a variety of lineages such as C-76 can overcome the challenges where farmers can irrigate less and be able to produce a decent yield in regions of West Texas and eastern New Mexico. On the basis of yield, C-76 and Val-C were selected for transcriptome analysis. Also, this is the first attempt on the genetic improvement of Valencia-type peanut to understand the drought tolerance mechanisms. When compared to DI *versus* FI, the more number of DEGs in Val-C genotype than C-76 infers its steep response to water stress. While, for the C-76, the treatment of the imposed stress was not sufficient to respond and could not be under stress, which could explain in terms of the expression of less number of DEGs during DI *versus* FI, implies its higher tolerance. Also, the comparative transcriptome analysis of both genotypes under FI conditions, the high expression of *proline imino-peptidase*, *peroxidase*, *abscisic acid 8-hydroxylase* and *photosystem II D2* genes in C-76 reflect its tolerance level and its ability to survive under limited water conditions. Although Val-C, is capable of activating different genes and pathways responsive to drought stress, decreased expression of key pathway genes related to ABA metabolism may result in cellular accumulation of ROS, which in turn may cause the devastating effects of the cell to resist water stress. Further, the decreased expression of the photosynthetic related genes, and genes related to sucrose metabolism may be one of the reasons for the reduced yield in Val-C. Also, identified SNPs related to *F-box protein* and *lipid transfer* genes showed polymorphism between C-76 and Val-C genotypes which can be used as linked markers for performing early generation selection in genomics-assisted breeding programs. In conclusion, this study revealed genotype information appropriate for the regions of West Texas and eastern New Mexico regions and the underlying complex mechanisms associated with contrasting peanut genotypes under water deficit conditions.

## Materials and Methods

### Germplasm and details of the experimental setup

Ten different peanut genotypes used in this study were obtained from four different market types, namely Spanish, Virginia, runner type and Valencia (Supplementary Table S1). Seven genotypes – ICGV 86388, ICGV 86051, TMV-2, Tamspan-90, Serenut-5R, Serenut-6T, and COC 041 belong to the Spanish market type, while ICGS 76 belongs to the Virginia group. While C-76-16 (C-76) is a breeding line which belongs to the runner type and Valencia-C (Val-C) belong to Valencia market. Detailed information on crop management, weather and meteorological conditions, soil moisture and temperature conditions have been provided in Supplementary Information). Field trials were conducted on a producer’s peanut field (Delwin Marrow Farm) in Terry County, Brownfield, Texas, USA (33° 18′N, 102° 16′W, elevation 1009 m). Two treatments, one with complete/full irrigation (FI) and the other with deficit irrigation (DI), was imposed. Complete irrigation received 100% available water content (AWC) while deficit irrigation received 50% throughout the growing season, from 2013 to 2015. Adequate irrigation (100%) was based on the farmer’s well capacity of the farmer’s wells, and the low irrigation (50%) were treatment was implemented by reducing the irrigation rate by 50%. The experiment was conducted on the basis of split-plot design using main plots with irrigation as a factor and each genotype was replicated four times. The collection of meteorological data, weather data and the total volume of water received (rain + rainfall + irrigation) as a percentage of the ETo (TWRPET) were provided in the Supplementary Information.

### Crop harvest and yield measurement

At physiological maturity, the crops were mechanically harvested in September in the FI and DI fields using a specialized digging equipment to dig peanuts under the vines (Pearman Corporation, GA, USA). Maturity was determined by examining the color of the pod mesocarp along the saddle region of the dorsal seed. Black or brown colors were used as indicators of physiological maturity. To analyze the color of the pods, 25 plants were randomly selected from the middle of the plot and were extracted using a rotating nozzle pressure washer.

### Leaf-level net photosynthesis

Photosynthesis at the leaf-level was measured between 60–90 days after sowing. For the fully irrigated control plants, photosynthesis was measured 48 h after an irrigation event. For the treatment of the deficit, measurements were taken during the interval of irrigation deficit interval (10 to 12 days) between the irrigation events. The recovery rates of photosynthesis for deficit treatments were measured to the plot after giving an irrigation for 48 h following 2 weeks of without irrigation. The rate of photosynthesis was measured using a portable photosynthesis system (Li-6400 XT, LI-COR, Inc.). All measurements were taken in between 10:00 and 12:00 h. For each genotype, three measurements per replicate were measured. The leaves of the Li-Cor 6400 XT cuvette were maintained and measured under the following conditions: saturated photosynthetic photon flux density (PPFD) of 2000 μmolm^−2^s^−1^, 40 Pa of CO_2_ and leaf temperature of 27 °C. When measuring gas exchange, carefully fill the sensor head with the leaflets to avoid errors.

### Total chlorophyll content and specific leaf area (SLA)

The chlorophyll content was recorded using SPAD (502 Minolta Corp., Ramsey, NJ). Measurements were taken every 30 days after emergence (DAE). The second or third fully developed tetrafoliate leaf was used to measure the SPAD reading when attached to the plant. After recording the measurements, the leaf was detached from the plant, placed in an envelope covered with a block of ice in an ice chest for the measurement of leaf area using a leaf area meter (Li 3050 C, LI-COR, Inc.), followed by drying in a hot air oven at 60 °C for 48 h to measure the dry weight.

The dry weight was measured to determine the specific leaf area (SLA) in cm^2^g^−1^ and determined using the formula:

$${\rm{SLA}}={\rm{Leaf}}\,{\rm{area}}\,({{\rm{cm}}}^{2})/{\rm{Leaf}}\,{\rm{dry}}\,{\rm{weight}}\,({\rm{g}})$$

### Statistical analysis

Data for each variable were analyzed using the PROC ANOVA procedure in Statistical Analysis System 9.3 (SAS Institute, 2013, https://www.sas.com/en_us/software/sas9.html). Before performing a separate analysis per year, the Bartlett variance, homogeneity of variance test was performed. In each year, irrigation and varieties were used as random factors, and appropriate error terms were used to calculate the mean sum of squares for each variable factor. If the F value was significant, a mean separation test was performed using the LSMEANS procedure in SAS software. In the LSMEANS procedure, additional PDIFF and PDMIX 800 options were used to evaluate the probability of significance among various main and interaction effects. Means were considered statistically different if *P-*value ≤0.05.

### RNA isolation, quality check, and sequencing

RNA-seq leaf samples were collected during 2014 from the fully developed third leaf of the apex. Three biological replicates were collected for profiling the transcriptome. Total RNA was extracted from 12 leaf tissues (two genotypes × two treatments × three biological replicates) of genotypes C-76 (tolerant) and Val-C (susceptible) during FI and DI conditions at the active pegging and fruit development stages using the TRIzol reagent (Life Technologies, Carlsbad, CA, USA). RNA purification was performed using the RNeasy Mini Kit (Qiagen, US) and the integrity of the RNA was verified using Bioanalyzer 2100 (Agilent Technologies, US). cDNA libraries for RNA-seq were constructed using Illumina TruSeq RNA Sample Preparation Kit (Illumina Inc., San Diego, CA, USA) following the manufacturer’s instructions. Paired-end sequencing (2 × 54 bp) was performed using the Illumina HiSeq 2000 (Illumina, San Diego, CA,USA) system at National Genomic Resource Center (NCGR).

### Reference-based assembly and annotation

RNA-seq data was analyzed using the Tuxedo pipeline^41^. The raw reads obtained from the sequencing were subjected to quality control using NGS-QCbox^42^ and trimmomatic v0.35^43^. The filtered reads of all samples were aligned to the A and B diploid progenitors of the peanut^18^ with Tophat v2.1.0^44^. The aligned reads of each sample were then used to create a Reference Annotation Based Transcript (RABT) assembly using cufflinks^45^. The resulting assemblies were then merged into a consensus assembly using cuffmerge for downstream analysis^41^ (Supplementary Fig. S9). The relationship between the transcriptomes of two genotypes (C-76 and Val-C) under two conditions (FI and DI) in three biological replicates on both A and B genomes was shown by generating a dendrogram by using the cummeRbund package^46^.

### Transcript abundance and identification of differentially expressed genes

Gene expression was estimated as fragments per kilobase of transcript per million mapped reads (FPKM) and differentially expressed genes were identified using Cuffdiff. Genes with $|lo{g}_{2}(fold\,change)|\ge 2$ and *P*-value ≤ 0.05 were identified as DEGs. The identified DEGs were scanned against the National Biotechnology Information Center (NCBI) non-redundant (*taxon*. Viridiplantae) protein database using BLASTX with an *E*-value cutoff of ≤10^−5^ to determine their putative function.

### Gene ontology (GO) and pathway analysis

Gene ontology for the expressed genes was performed using Blast2GO v 3.3^47^. In parallel, the expressed genes were searched against Plant TFDB with an *E*-value cutoff of ≤10^−10^ to identify the genes encoding the transcription factors. The pathway analysis was then carried out using the KEGG database. Heat maps with expression profiles were generated based on the transformed FPKM values using MeV^48^.

### SNP identification

The identification of SNPs was performed using SAMtools^49^, followed by their annotation using the SnpEff program^50^. The BatchPrimer3 tool (https://probes.pw.usda.gov/cgi-bin/batchprimer3/batchprimer3.cgi) was used to design allele-specific primers between C-76 and Val-C. The information on primers has been provided in Supplementary Tables S10 and S11. Validation of SNPs was performed by qRT-PCR using SYBR green chemistry. Homozygous and heterozygous alleles were detected based on variations in the Ct values obtained^51^.

### Quantitative real-time PCR (qRT-PCR)

The real-time qPCR was performed on the Applied Biosystems QuantStudioTM7 Flex real-time PCR system (Life Technologies, Carlsbad, CA, USA) using RT2 SYBR Green ROX qPCR master mix chemistry (Qiagen, CA, USA). Three biological replicates per peanut genotype (C-76 and Val-C) and treatment (FI and DI), and three technical replicates per biological sample were processed by qPCR in real-time for each set of SNP primers. Each biological sample was analyzed with three technical replicates with the *Actin* as housekeeping gene. The conditions used for the amplification were as follows: 2 min at 50 °C, 10 min at 95 °C, followed by 45 cycles of 15 s at 95 °C, 1 min at 58 °C and analysis of the dissociation curve of 15 s at 95 °C, 1 min 58 °C, 15 s at 95 °C and 15 s at 60 °C. Expression levels were normalized at actin expression levels for each sample^52^. The relative fold change (log2) of the normalized data was represented by the 2^−ΔΔCt^ method^53^.

## Supplementary information

Supplementary information.

## Data Availability

Sequencing RNA-seq data described in this article have been published in the National Biotechnology Center Archives database with BioProject IDPRJNA498570.
