# Supplementary material for: Genome-wide transcriptome and physiological analyses provide new insights into peanut drought response mechanisms
Source: Sci Rep. 2020 Mar 5;10:4071. doi: 10.1038/s41598-020-60187-z (PMC7058030; doi:10.1038/s41598-020-60187-z)
Supplement: Supplementary file 1 — Supplementary information. [file 41598_2020_60187_MOESM1_ESM.pdf]

Genome-wide transcriptome and physiological analyses provide new insights into peanut drought response mechanisms

Sailaja Bhogireddy[1,$], Abishek Xavier[2,$], Vanika Garg[1,$], Nancy Layland[3], Renee Arias[4], Paxton Payton[5], Spurthi N. Nayak[6], Manish K. Pandey[1], Naveen Puppala[2*], Rajeev K. Varshney[1*]

[1]International Crops Research Institute for the Semi-Arid Tropics (ICRISAT), Hyderabad, India

[2]New Mexico State University, Agricultural Science Center at Clovis, New Mexico, USA

[3]USDA FSIS OFO, Dallas, USA

[4]USDA-ARS, National Peanut Research Laboratory, Dawson, USA

5USDA-ARS, Cropping Systems Research Laboratory, Lubbock, USA

[6]University of Agricultural Sciences, Dharwad, India

[$]Authors contributed equally

[*]Corresponding authors: npuppala@nmsu.edu; r.k.varshney@cgiar.org

Table S1: List of the selected peanut genotypes used in the present study

S. No.	Name of the cultivar	Market type	Plant Introduction	Sub species	Reference and year of release
1	ICGV 86388	Spanish	PI 593239	*fastigiata*	Dwivedi et al., 1996 [54]
2	ICGV 86015	Spanish	PI 585005	*fastigiata*	Nigam et al., 1995 [55]
3	TAMSPAN 90	Spanish	PI 550721	*fastigiata*	Smith et al., 1998 [56]
4	Serenut 5R	Spanish	PI 676092	*fastigiata*	Okello et al., 2016 [57]
5	Serenut 6T	Spanish	PI 682726	*fastigiata*	Okello et al., 2018 [58]
6	COC041	Spanish	PI 493631	*fastigiata*	Holbrook and Dong, 2005 [59]
7	ICGS 76	Virginia	PI 546372	*hypogaea*	Nigam et al., 1991 [60]
8	C76-16	Runner	unreleased	*hypogaea*	Holbrook unreleased
9	Valencia-C	Valencia	PI 355987	*fastigiata*	Hsi, 1980 [61]
10	TMV2	Spanish	Released variety	*fastigiata*	Tamil Nadu Ag. Uni., India [62]

Table S2: Significant and non-significant factors in combined and year wise analysis of variance for yield

Source	2013	2 014	2015	Combined
Irrigation Treatment	***	***	**	***
Replication	NS	***	NS	NS
Genotypes	***	**	***	***
Irrigation Trt x Genotypes	***	*	*	**

*: p value<0.05

**: p value<0.01

***: p value< 0.0001

NS: Non-Significant (p value>=0.05)

Table S3: Three-year average of irrigation treatment on peanut pod yield at Brownfield, Texas

Treatment	Pod yield (Kg ha^{-1})			
	**2013**	**2014**	**2015**	**Combined**
Full irrigation	2828	4507	4131	3822
Deficit irrigation	1879	1469	3613	2320
LSD	487.78	827.32	886.45	748.53

Table S4: Number of mapped reads (in millions) to the genomic regions of A and B genomes of peanut

	A-genome				B-genome			
**Sample Name**	**Reads mapped on A genome**	**Mapped reads on exonic region**	**Mapped reads on intronic region**	**Mapped reads on intergenic region**	**Reads mapped on B genome**	**Mapped reads on exonic region**	**Mapped reads on intronic region**	**Mapped reads on intergenic region**
C-76-I	25133223	18,571,191 (80.9%)	1,423,642 (6.2%)	2,935,998 (12.8%)	25187811	18,505,052 (80.8%)	1,472,063 (6.4%)	2,902,549 (12.6%)
C-76-I	33385577	25,117,881 (80.8%)	1,919,919 (6.1%)	4,038,523 (13%)	33328532	24,722,874 (80.8%)	1,788,118 (5.8%)	4,067,669 (13.3%)
C-76-I	34042312	24,924,015 (78.9%)	2,317,281 (7.3%)	4,313,015 (13.6%)	34030525	24,642,074 (79.1%)	2,056,062 (6.6%)	4,437,985 (14.2%)
C-76-S	28109209	21,468,853 (82.2%)	1,732,072 (6.6%)	2,902,934 (11.1%)	28197948	21,332,690 (82.1%)	1,714,585 (6.6%)	2,913,117 (11.2%)
C-76-S	25193642	18,932,536 (82.2%)	1,438,205 (6.2%)	2,662,603 (11.5%)	25251860	18,779,946 (81.9%)	1,446,468 (6.3%)	2,687,056 (11.7%)
C-76-S	24867006	18,776,355 (81.1%)	1,558,956 (6.8%)	2,587,768 (11.2%)	24837478	18,550,248 (81.9%)	1,477,426 (6.5%)	2,620,095 (11.5%)
Val-C-I	26736043	20,439,083 (82.5%)	1,458,002 (5.8%)	2,953,847 (11.8%)	26814433	20,282,232 (82.1%)	1,530,815 (6.2%)	2,881,020 (11.6%)
Val-C-I	25445709	19,281,804 (81.7%)	1,258,005 (5.3%)	3,098,804 (13.1%)	25490501	19,156,149 (81.5%)	1,363,737 (5.8%)	2,962,334 (12.6%)
Val-C-I	24735311	19,424,337 (84.3%)	1,324,071 (5.7%)	2,312,992 (10.0%)	24784990	19,100,958 (83.8%)	1,352,824 (5.9%)	2,317,990 (10.1%)
Val-C-S	27166927	20,456,696 (80.9%)	1,269,194 (5.0%)	3,563,843 (14.0%)	27167978	20,365,423 (81.0%)	1,297,374 (5.1%)	3,464,733 (13.7%)
Val-C-S	28993197	20,954,382 (77.9%)	1,640,015 (6.0%)	4,377,419 (16.2%)	28968562	20,667,793 (77.6%)	1,588,332 (5.9%)	4,360,194 (16.3%)
Val-C-S	25406521	19,026,778 (81.5%)	1,527,256 (6.5%)	2,892,081 (12.3%)	25436515	18,814,043 (81.1%)	1,458,243 (6.2%)	2,926,270 (12.6%)

Table S5: List of the common differentially expressed genes identified between C-76 and Val-C during full irrigation and deficit irrigation (DI) conditions

Gene Id	Locus	C-76-I (FPKM)	Val-C-I (FPKM)	C-76-DI (FPKM)	Val-C-DI (FPKM)	Annotation
-	Aradu.A01:22588649-22589716	5.6	0.2	0.3	3.5	Acetyltransferase (Gnat) Domain
Aradu.1W5FD	Aradu.A02:7550917-7554786	4.6	0.3	0.3	9.6	Bon1-Associated 2-Like
Aradu.80YRY	Aradu.A06:91252607-91255011	18.1	235.1	3.5	17.3	Flowering Locus T
-	Aradu.A04:44982507-44983397	4.0	0.5	0.7	3.1	Formin 5-Like
-	Aradu.A07:564179-565023	23.0	3.2	4.5	26.3	Hypothetical Protein
-	Aradu.A06:107807670-107808240	22.0	2.0	3.6	21.5	Indole-3-Acetic Acid-Induced Arg7
Aradu.RU8HS	Aradu.A10:104125539-104130118	5.0	0.4	0.8	3.6	Late Embryogenesis Abundant
-	Aradu.A09:19492157-19492961	7.9	1.7	0.9	9.9	LRR receptor serine threonine tyrosine kinase
Aradu.I2J7R	Aradu.A04:7915685-7919707	0.3	6.8	1.8	55.8	LOB domain-containing 4-like
Aradu.BX6BE	Aradu.A08:30519573-30521204	5.3	1.3	0.3	10.9	Malonyl-co A:anthocyanin 3-O-glucoside-6 -O-malonyltransferase
-	Aradu.A03:123850610-123851151	30.3	5.4	6.0	27.6	-
-	Aradu.A05:54816004-54816860	3.5	0.2	0.6	3.3	-
Aradu.CP3UH	Aradu.A02:88923822-88930409	12.9	3.2	2.5	18.0	Phenazine Biosynthesis Family
Aradu.78K64	Aradu.A09:8043188-8046037	18.0	0.5	1.2	9.3	Plastocyanin-Like Domain
Aradu.PEA62	Aradu.A10:30590205-30600505	8.0	0.7	0.8	4.3	Replication Factor-A Carboxy-Terminal
Aradu.M8IZW	Aradu.A10:10708749-10710803	3.6	0.5	0.5	2.4	Sufe Chloroplastic Isoform X1
-	Aradu.A05:82781943-82783188	1.2	94.6	14.5	640.8	Trypsin Inhibitor
-	Aradu.A07:15133893-15135572	3.7	0.7	0.5	3.8	U-Box Domain-Containing 21-Like
-	Aradu.A06:19507802-19508489	11.3	1.4	1.1	16.0	Zinc Finger Zat11-Like

Table S6: List of differentially expressed genes in different combinations during deficit irrigation (DI) stress on A and B genomes

Gene Id	Annotation	Sample1	Sample2	Value 1 (FPKM)	Value 2 (FPKM)	log 2 fold
Aradu.I2J7R	LOB domaincontaining 4	C-76-FI-A	C-76-DI-A	0.33	1.79	2.44
Aradu.T9EI4	ABC transporter G family member 15	C-76-FI-A	C-76-DI-A	0.37	1.49	2.02
Aradu.WC0YR	Heavy Metal Transport Detoxification	C-76-FI-A	C-76-DI-A	11.57	0.32	-5.18
Aradu.XW8AZ	Probable Nucleoredoxin 1	C-76-FI-A	C-76-DI-A	3.57	0.13	-4.80
Aradu.Q3KFX	Wrky Transcription Factor 70	C-76-FI-A	C-76-DI-A	5.16	0.23	-4.48
Aradu.1W5FD	Cytochrome P450 94A1	C-76-FI-A	C-76-DI-A	4.58	0.27	-4.06
Aradu.P4T20	Linoleate 13Slipoxygenase 2 Chloroplastic	C-76-FI-A	C-76-DI-A	66.33	4.23	-3.97
Aradu.38TXW	Probable Receptor Kinase	C-76-FI-A	C-76-DI-A	7.56	0.61	-3.63
Aradu.VE705	Sufe Chloroplastic Isoform X1	C-76-FI-A	C-76-DI-A	12.82	1.05	-3.60
Aradu.289WG	Abc Transporter G Family Member 15	C-76-FI-A	C-76-DI-A	9.28	0.89	-3.38
Aradu.KD2FK	Probable Mitochondrial Chaperone Bcs1B	C-76-FI-A	C-76-DI-A	5.70	0.57	-3.33
Aradu.49DKF	E3 Ubiquitin Ligase Rglg2	C-76-FI-A	C-76-DI-A	2.36	0.24	-3.33
Aradu.83N8C	Nitrate Transporter	C-76-FI-A	C-76-DI-A	1.28	0.14	-3.20
Aradu.Y1LYG	Peroxidase 47	C-76-FI-A	C-76-DI-A	11.22	1.30	-3.11
Aradu.08UMR	Lysine Histidine Transporter 1	C-76-FI-A	C-76-DI-A	1.53	0.18	-3.09
Aradu.1S9TS	Ubox Domaincontaining 21	C-76-FI-A	C-76-DI-A	66.31	7.84	-3.08
Aradu.Y3QBI	Isoflavone 7Omethyltransferase	C-76-FI-A	C-76-DI-A	4.66	0.57	-3.03
Araip.56XNF	Leucoanthocyanidin Dioxygenase	C-76-FI-B	C-76-DI-B	1.23	5.04	2.03
Araip.8C4ZH	Kunitz Trypsin Protease Inhibitor	C-76-FI-B	C-76-DI-B	1.32	19.22	3.87
Araip.A0CQ3	Lectin Precursor	C-76-FI-B	C-76-DI-B	1.34	7.21	2.43
Araip.BLF65	Cystatin	C-76-FI-B	C-76-DI-B	3.37	16.38	2.28
Araip.RBQ5E	Abc Transporter G Family Member 15	C-76-FI-B	C-76-DI-B	0.33	1.67	2.33
Araip.RJJ5V	LOBDomaincontaining 4	C-76-FI-B	C-76-DI-B	0.32	1.63	2.34
Araip.ZD9NB	Cytochrome P450 94A1	C-76-FI-B	C-76-DI-B	3.80	0.15	-4.66
Araip.I5AXB	Acetyltransferase (Gnat) Domain	C-76-FI-B	C-76-DI-B	8.43	0.33	-4.66
Araip.WY6XF	Heavy Metalassociated Isoprenylated Plant 26	C-76-FI-B	C-76-DI-B	5.11	0.27	-4.22
Araip.M7BCB	Probable LRR Serine Threonine Kinase	C-76-FI-B	C-76-DI-B	2.57	0.14	-4.20
Araip.13WAT	HXXXDtype acyltransferase family	C-76-FI-B	C-76-DI-B	6.08	0.36	-4.09
Araip.Q2JPR	BON1associated 2	C-76-FI-B	C-76-DI-B	3.06	0.18	-4.06

Araip.T4J1X	Unknown	C-76-FI-B	C-76-DI-B	2.67	0.21	-3.68
Araip.PQ3Y0	Salicylic Acid Carboxyl Methyltransferase	C-76-FI-B	C-76-DI-B	2.66	0.21	-3.66
Araip.D6MF0	Probable Receptor Kinase At1G11050	C-76-FI-B	C-76-DI-B	1.71	0.14	-3.66
Araip.WHJ14	WRKY Transcription Factor 40	C-76-FI-B	C-76-DI-B	13.49	1.14	-3.57
Araip.UJR3L	Zinc Finger Zat11	C-76-FI-B	C-76-DI-B	12.16	1.10	-3.47
Araip.20FU5	Probable Wrky Transcription Factor 40	C-76-FI-B	C-76-DI-B	6.23	0.60	-3.38
Araip.P8RKN	Peroxidase 47	C-76-FI-B	C-76-DI-B	1.15	0.12	-3.29
Araip.67HK3	Glycinerich Rnabinding Mitochondrial	C-76-FI-B	C-76-DI-B	4.28	0.45	-3.24
Araip.BF1SB	Probable Calciumbinding Cml41	C-76-FI-B	C-76-DI-B	6.00	0.64	-3.22
Araip.HGI2J	Linoleate 13Slipoxygenase 2 Chloroplastic	C-76-FI-B	C-76-DI-B	11.46	1.24	-3.21
Araip.E4AWE	Transmembrane Protein,	C-76-FI-B	C-76-DI-B	89.57	9.72	-3.20
Araip.J0W46	Senescencespecific Cysteine Protease Sag39	C-76-FI-B	C-76-DI-B	145.76	17.37	-3.07
Araip.ZS1T2	Fbox At1G61340	C-76-FI-B	C-76-DI-B	3.24	0.39	-3.04
Araip.RB0W1	Lysine Histidine Transporter 1	C-76-FI-B	C-76-DI-B	2.66	0.33	-3.02
Araip.J5GRU	7Methylxanthosine Synthase 1	C-76-FI-B	C-76-DI-B	2.16	0.27	-3.02
Aradu.02IKS	Glutamine Dumper 5	C-76-DI-A	Val-C-DI-A	0.28	1.77	2.64
Aradu.SMZ6S	Bromodomaincontaining Factor 1 Isoform X2	C-76-DI-A	Val-C-DI-A	4.22	0.05	-6.43
Aradu.1H6YX	Sieve Element Occlusion C	C-76-DI-A	Val-C-DI-A	8.17	0.12	-6.03
Aradu.93Y3Y	Ulp1 Protease Carboxyterminal Domain	C-76-DI-A	Val-C-DI-A	23.01	0.37	-5.97
Aradu.ZV9H6	Serine Threonine Kinase Tousled	C-76-DI-A	Val-C-DI-A	6.49	0.13	-5.70
Aradu.CMH7M	Oxidoreductase Transition Metal Ionbinding	C-76-DI-A	Val-C-DI-A	33.79	0.68	-5.63
Aradu.24J3X	6Binteracting 2	C-76-DI-A	Val-C-DI-A	1.72	0.04	-5.34
Aradu.398CK	Receptor Kinase 2	C-76-DI-A	Val-C-DI-A	0.98	0.03	-5.25
Aradu.TYN4X	Lrr Receptor Serine Threonine Kinase	C-76-DI-A	Val-C-DI-A	4.36	0.17	-4.64
Aradu.0XN7S	Epidermal Growth Factor Receptor Substrate 15 1	C-76-DI-A	Val-C-DI-A	10.54	0.43	-4.60
Aradu.42UNZ	Serine Threonine Kinase Fray2	C-76-DI-A	Val-C-DI-A	3.87	0.18	-4.44
Aradu.E00AS	Coldregulated 413 Plasma Membrane 1	C-76-DI-A	Val-C-DI-A	30.77	1.44	-4.42
Aradu.8Q8DX	E3 Ubiquitin Ligase Rf298	C-76-DI-A	Val-C-DI-A	6.48	0.31	-4.39
Aradu.5H0AD	Lrr Receptor Serine Threonine Kinase Fls2	C-76-DI-A	Val-C-DI-A	20.58	1.04	-4.30
Aradu.IAN0B	Kinesin4 Isoform X1	C-76-DI-A	Val-C-DI-A	2.70	0.14	-4.24
Aradu.92LSD	Hypothetical Protein Phavu_003G055000G	C-76-DI-A	Val-C-DI-A	52.33	3.27	-4.00
Aradu.61A42	Cytochrome P450 78A5	C-76-DI-A	Val-C-DI-A	22.38	1.43	-3.96
Aradu.G8P6Y	Fruitfull Partial	C-76-DI-A	Val-C-DI-A	2.50	0.18	-3.79

Aradu.EAG5M	Vacuolarsorting Receptor 1	C-76-DI-A	Val-C-DI-A	1.02	0.07	-3.78
Aradu.32P5Q	Chloroplastic	C-76-DI-A	Val-C-DI-A	7.64	0.57	-3.75
Aradu.D8WVG	Proline Iminopeptidase	C-76-DI-A	Val-C-DI-A	22.25	1.70	-3.71
Aradu.ZH9NQ	Aspartyl Glutamyltrna(Asn Gln) Amidotransferase	C-76-DI-A	Val-C-DI-A	2.40	0.19	-3.69
Aradu.PD37S	Sucrose Synthase 6	C-76-DI-A	Val-C-DI-A	3.43	0.29	-3.58
Aradu.QVM9Q	Sucrase Ferredoxin Family	C-76-DI-A	Val-C-DI-A	39.94	3.39	-3.56
Aradu.15WFY	Phosphatidylinositol:Ceramide Inositol-p-transferase	C-76-DI-A	Val-C-DI-A	2.69	0.23	-3.52
Aradu.M2FWD	Dnadirected Rna Polymerase I Subunit Rpa1	C-76-DI-A	Val-C-DI-A	10.38	0.91	-3.52
Aradu.Q4CGX	Ubiquitin Ligase	C-76-DI-A	Val-C-DI-A	6.51	0.57	-3.50
Aradu.L67UJ	Hmg (High Mobility Group) Box	C-76-DI-A	Val-C-DI-A	3.82	0.34	-3.49
Aradu.RY9LY	Glucan Endo1,3Betaglucosidase 12	C-76-DI-A	Val-C-DI-A	2.91	0.26	-3.46
Aradu.W9WVE	Hypothetical Protein Glysoja_041312	C-76-DI-A	Val-C-DI-A	0.81	0.07	-3.44
Aradu.RA346	Sel1 Repeatcontaining L18	C-76-DI-A	Val-C-DI-A	13.36	1.24	-3.43
Aradu.ZXE5G	Pyruvate Dehydrogenase E1 Component Mitochondri	C-76-DI-A	Val-C-DI-A	3.20	0.30	-3.42
Aradu.42MBK	Udpglucuronic Acid Decarboxylase 5	C-76-DI-A	Val-C-DI-A	5.58	0.53	-3.40
Aradu.B9QD5	Gtype Lectin Sreceptor Serine Threonine Kinase	C-76-DI-A	Val-C-DI-A	1.58	0.16	-3.34
Aradu.NP81U	Metacaspase3 Isoform X1	C-76-DI-A	Val-C-DI-A	1.48	0.15	-3.31
Aradu.DA392	Leucinerich Repeat Receptor Kinase	C-76-DI-A	Val-C-DI-A	5.75	0.58	-3.31
Aradu.H8XF8	Cathepsin B	C-76-DI-A	Val-C-DI-A	8.10	0.83	-3.29
Aradu.2EU9J	Adpribosylation Factor Gtpaseactivating Agd12	C-76-DI-A	Val-C-DI-A	6.19	0.64	-3.28
Aradu.N441K	Pleiotropic Drug Resistance 3	C-76-DI-A	Val-C-DI-A	0.87	0.09	-3.27
Aradu.V3HC5	Upf0187 Chloroplastic	C-76-DI-A	Val-C-DI-A	4.72	0.50	-3.24
Aradu.ABT8D	Eh Domaincontaining 1	C-76-DI-A	Val-C-DI-A	5.38	0.59	-3.20
Aradu.I7LA7	Myosinj Heavy Chain	C-76-DI-A	Val-C-DI-A	76.43	8.40	-3.19
Aradu.65DGV	Uncharacterized Protein Loc100810300 Isoform X1	C-76-DI-A	Val-C-DI-A	32.07	3.54	-3.18
Aradu.JI62L	Transcription Factor Bhlh61	C-76-DI-A	Val-C-DI-A	28.96	3.20	-3.18
Aradu.G0THS	Hypothetical Protein Prupe_Ppa011961Mg	C-76-DI-A	Val-C-DI-A	2.85	0.33	-3.13
Aradu.Z0G82	Acetylco-A Carboxylase Carboxyl Transferase	C-76-DI-A	Val-C-DI-A	1.70	0.20	-3.11
Aradu.90PU5	Ferricchelate Reductase 1	C-76-DI-A	Val-C-DI-A	4.45	0.52	-3.11
Aradu.639I9	Cytochrome P450 89A2	C-76-DI-A	Val-C-DI-A	3.94	0.48	-3.04
Aradu.73Q5I	Structural Maintenance Of Chromosomes 21	C-76-DI-A	Val-C-DI-A	4.21	0.52	-3.02
Aradu.DF5FL	Endo1,4Betaxylanase A	C-76-DI-A	Val-C-DI-A	1.26	0.16	-3.00
Aradu.W6H6W	Hypothetical Protein Glysoja_015001	C-76-DI-A	Val-C-DI-A	1.07	8.72	3.03

Aradu.YL3ZN	Udpglucuronic Acid Decarboxylase 6 Isoform X1	C-76-DI-A	Val-C-DI-A	1.11	9.12	3.04
Aradu.47XL4	Gdsl Esterase Lipase Exl3	C-76-DI-A	Val-C-DI-A	0.36	2.93	3.04
Aradu.RXA66	Cblinteracting Serine Threonine Kinase 14	C-76-DI-A	Val-C-DI-A	1.46	12.05	3.05
Aradu.EI6YW	Uncharacterized Protein Loc102665099	C-76-DI-A	Val-C-DI-A	42.48	352.83	3.05
Aradu.7M2ZA	Phenolic Glucoside Malonyltransferase 1	C-76-DI-A	Val-C-DI-A	10.93	90.93	3.06
Aradu.82DSF	Glutathione Stransferase F9	C-76-DI-A	Val-C-DI-A	0.27	2.27	3.06
Aradu.Z5Y8Q	Pathogenesisrelated Bet V I Family	C-76-DI-A	Val-C-DI-A	2.93	24.46	3.06
Aradu.67IGJ	Cysteinerich Repeat Secretory 55	C-76-DI-A	Val-C-DI-A	0.13	1.13	3.06
Aradu.54FFT	Miraculin	C-76-DI-A	Val-C-DI-A	3.96	33.12	3.06
Aradu.J6XSM	Serine Carboxypeptidase	C-76-DI-A	Val-C-DI-A	0.29	2.48	3.10
Aradu.AW9GY	Nucleobaseascorbate Transporter 11	C-76-DI-A	Val-C-DI-A	9.98	85.73	3.10
Aradu.G09L8	Coldregulated 413 Plasma Membrane 2	C-76-DI-A	Val-C-DI-A	0.75	6.44	3.10
Aradu.M1N9L	Alpha Beta Fold Hydrolase	C-76-DI-A	Val-C-DI-A	0.85	7.29	3.11
Aradu.9IJ7G	Wrky Transcription Factor 23	C-76-DI-A	Val-C-DI-A	0.09	0.81	3.12
Aradu.751D4	Ovate Transcriptional Repressor	C-76-DI-A	Val-C-DI-A	0.32	2.76	3.13
Aradu.XEX7M	Pathogenesisrelated 1	C-76-DI-A	Val-C-DI-A	0.89	7.79	3.13
Aradu.H9B5W	Duf1677 Family	C-76-DI-A	Val-C-DI-A	1.47	12.90	3.13
Aradu.CNF9F	Probable Galacturonosyltransferase 1	C-76-DI-A	Val-C-DI-A	9.75	85.93	3.14
Aradu.PRD5G	Glutathione Stransferase	C-76-DI-A	Val-C-DI-A	0.20	1.81	3.15
Aradu.M7NEJ	Cytochrome P450 82G1	C-76-DI-A	Val-C-DI-A	1.98	17.59	3.15
Aradu.D7NJ0	Sufe Chloroplastic Isoform X1	C-76-DI-A	Val-C-DI-A	0.18	1.63	3.16
Aradu.RYN4L	Transmembrane Protein,	C-76-DI-A	Val-C-DI-A	5.86	52.26	3.16
Aradu.H563E	Kda Class II Heat Shock	C-76-DI-A	Val-C-DI-A	0.80	7.12	3.16
Aradu.LIS00	Udpglycosyltransferase 83A1	C-76-DI-A	Val-C-DI-A	0.75	6.71	3.17
Aradu.EQ6UY	Hypothetical Protein Mtr_1G114340	C-76-DI-A	Val-C-DI-A	2.63	23.80	3.18
Aradu.7FC0G	Fatty Acyl Reductase 3	C-76-DI-A	Val-C-DI-A	1.07	9.77	3.19
Aradu.QY0RJ	Oacyltransferase Wsd1	C-76-DI-A	Val-C-DI-A	0.64	5.81	3.19
Aradu.QI99C	Expansina8 Precursor	C-76-DI-A	Val-C-DI-A	1.37	12.60	3.21
Aradu.203ID	Hydroxysteroid 11Betadehydrogenase 1	C-76-DI-A	Val-C-DI-A	0.25	2.36	3.22
Aradu.70DQ7	Malonylco-A:Anthocyanin 3Ogluco6malonyltransfer	C-76-DI-A	Val-C-DI-A	0.58	5.44	3.24
Aradu.W8QUD	Notum Homolog	C-76-DI-A	Val-C-DI-A	0.32	3.07	3.24
Aradu.0572C	Plastid Movement Impaired	C-76-DI-A	Val-C-DI-A	0.07	0.66	3.24
Aradu.L3QY1	Pathogenesisrelated Class 10	C-76-DI-A	Val-C-DI-A	0.17	1.63	3.27

Aradu.I8HGC	Udpglucosyltransferase Family	C-76-DI-A	Val-C-DI-A	3.46	33.39	3.27
Aradu.8L0QQ	Ralf 34	C-76-DI-A	Val-C-DI-A	0.73	7.09	3.28
Aradu.WB4HU	21 Kda	C-76-DI-A	Val-C-DI-A	0.31	2.99	3.28
Aradu.TZ1M5	Nonsymbiotic Hemoglobin	C-76-DI-A	Val-C-DI-A	0.87	8.49	3.29
Aradu.N2VA8	Rac Gtpbinding Rac2	C-76-DI-A	Val-C-DI-A	0.12	1.19	3.29
Aradu.04RDY	Serine Threonine Phosphatase 6 Regulatory Ankyrin	C-76-DI-A	Val-C-DI-A	85.71	841.72	3.30
Aradu.Y2TN0	Hypothetical Protein Mtr_5G017880	C-76-DI-A	Val-C-DI-A	0.23	2.26	3.31
Aradu.GIN82	Eukaryotic Aspartyl Protease Family	C-76-DI-A	Val-C-DI-A	0.79	8.07	3.35
Aradu.5M2H3	Senescencespecific Cysteine Protease Sag39	C-76-DI-A	Val-C-DI-A	32.20	330.54	3.36
Aradu.1S3NC	Coiledcoil Domaincontaining 132 Isoform X1	C-76-DI-A	Val-C-DI-A	0.41	4.26	3.38
Aradu.X06N9	CMPNacetylneuramingalactosamide23Sialyltransfera	C-76-DI-A	Val-C-DI-A	0.47	4.92	3.38
Aradu.XU5YD	Nonspecific Lipidtransfer 1	C-76-DI-A	Val-C-DI-A	1.24	13.22	3.41
Aradu.AE0GD	Flowering Locus T	C-76-DI-A	Val-C-DI-A	8.25	87.83	3.41
Aradu.348PZ	Probable Lrr Receptor Serine Threonine Kinase	C-76-DI-A	Val-C-DI-A	3.82	40.74	3.42
Aradu.L1HGF	Duf4228 Domain	C-76-DI-A	Val-C-DI-A	0.71	7.67	3.44
Aradu.0N518	Hypothetical Protein Glysoja_027391, Partial	C-76-DI-A	Val-C-DI-A	0.46	5.06	3.45
Aradu.8FL49	Zip Zinc Iron Transport Family	C-76-DI-A	Val-C-DI-A	6.78	74.49	3.46
Aradu.M0ENQ	Ei24 Homolog Isoform X2	C-76-DI-A	Val-C-DI-A	0.38	4.24	3.47
Aradu.C09GA	Xylem Cysteine Ase 2	C-76-DI-A	Val-C-DI-A	1.32	14.56	3.47
Aradu.P0ZYD	Cation Calcium Exchanger 2	C-76-DI-A	Val-C-DI-A	16.27	180.73	3.47
Aradu.0L5CU	1Deoxydxylulose5Phosphate Synthase	C-76-DI-A	Val-C-DI-A	2.58	28.64	3.47
Aradu.G235T	Crib Domaincontaining Ric7	C-76-DI-A	Val-C-DI-A	2.23	24.92	3.48
Aradu.1P1D6	Isoflavone Reductase	C-76-DI-A	Val-C-DI-A	14.77	166.36	3.49
Aradu.IR2XG	Hypothetical Protein Poptr_0017S11350G	C-76-DI-A	Val-C-DI-A	1.39	15.65	3.50
Aradu.31AY0	Probable Auxin Efflux Carrier Component 1B	C-76-DI-A	Val-C-DI-A	0.84	9.53	3.50
Aradu.ZR9ZC	Lectin	C-76-DI-A	Val-C-DI-A	0.18	2.06	3.50
Aradu.CYG6N	Legume Lectin Beta Domain	C-76-DI-A	Val-C-DI-A	5.35	61.30	3.52
Aradu.JZT16	Hxxxdtype Acyltransferase Family	C-76-DI-A	Val-C-DI-A	2.38	27.33	3.52
Aradu.M2RVT	Serine Carboxypeptidase 20	C-76-DI-A	Val-C-DI-A	0.14	1.58	3.54
Aradu.Z6652	Glucan Endo1,3Betaglucosidase 3	C-76-DI-A	Val-C-DI-A	0.54	6.35	3.56
Aradu.NN4ME	Probable Membraneassociated Kinase Regulator 6	C-76-DI-A	Val-C-DI-A	0.07	0.85	3.56
Aradu.MJ134	Uncharacterized Protein Loc100779898	C-76-DI-A	Val-C-DI-A	4.97	58.65	3.56
Aradu.F8AYK	Ribonuclease 1	C-76-DI-A	Val-C-DI-A	0.88	10.74	3.60

Aradu.P6ZPS	Alternative NAD(P)Hubiquinone Oxidoreductase	C-76-DI-A	Val-C-DI-A	14.98	182.49	3.61
Aradu.440M4	Dnabinding Escarola	C-76-DI-A	Val-C-DI-A	16.78	204.78	3.61
Aradu.ZPP4Z	Tubulin Beta1 Chain	C-76-DI-A	Val-C-DI-A	0.53	6.49	3.61
Aradu.16IRP	Endo1,3 1,4Betadglucanase	C-76-DI-A	Val-C-DI-A	0.10	1.17	3.62
Aradu.Z9Y3J	Trichome Berefringence 7	C-76-DI-A	Val-C-DI-A	0.42	5.21	3.62
Aradu.EWL0R	Legume Lectin Beta Domain	C-76-DI-A	Val-C-DI-A	0.13	1.66	3.62
Aradu.J1ZY0	Glutathione S Aminoterminal Domain	C-76-DI-A	Val-C-DI-A	0.23	2.85	3.63
Aradu.0R425	Zinc Finger Bed Domaincontaining Ricesleeper 2	C-76-DI-A	Val-C-DI-A	0.56	7.05	3.65
Aradu.Q4J8J	Gtype Lectin Sreceptor Serine Threonine Kinase Rlk	C-76-DI-A	Val-C-DI-A	0.30	3.78	3.65
Aradu.0A8NK	Dnaj Heat Shock Family	C-76-DI-A	Val-C-DI-A	2.60	32.52	3.65
Aradu.XUN5S	Hypothetical Protein Phavu_010G061000G	C-76-DI-A	Val-C-DI-A	0.25	3.11	3.65
Aradu.B1FJV	Abc Transporter C Family Member 10	C-76-DI-A	Val-C-DI-A	0.27	3.47	3.67
Aradu.08UAJ	Nim1Interacting 1	C-76-DI-A	Val-C-DI-A	17.18	218.54	3.67
Aradu.M62BY	Sucrose Transport Suc1	C-76-DI-A	Val-C-DI-A	0.84	10.72	3.68
Aradu.0M1UL	Lactoylglutathione Lyase	C-76-DI-A	Val-C-DI-A	2.67	34.22	3.68
Aradu.17DUP	Arabinogalactan Peptide 20	C-76-DI-A	Val-C-DI-A	0.59	7.57	3.68
Aradu.QVF0N	Replication Factora Carboxyterminal Domain	C-76-DI-A	Val-C-DI-A	0.57	7.40	3.69
Aradu.7065G	14 Kda Prolinerich	C-76-DI-A	Val-C-DI-A	0.71	9.21	3.69
Aradu.U7IGL	Bifunctional Polynucleotide Phosphatase Kinase	C-76-DI-A	Val-C-DI-A	0.73	9.46	3.70
Aradu.9I4XH	Seed Linoleate 9Slipoxygenase	C-76-DI-A	Val-C-DI-A	0.87	11.40	3.70
Aradu.TEW52	Extracellular Ligandgated Ion Channel	C-76-DI-A	Val-C-DI-A	0.48	6.47	3.75
Aradu.B90GQ	Villin4 Isoform X1	C-76-DI-A	Val-C-DI-A	6.32	85.06	3.75
Aradu.54P9X	Polyphenol Oxidase Chloroplastic	C-76-DI-A	Val-C-DI-A	4.32	58.30	3.76
Aradu.G3DHZ	Lrr Receptor Serine Threonine Kinase Gso2	C-76-DI-A	Val-C-DI-A	0.85	11.53	3.77
Aradu.T939V	Receptor Kinase At1G72540	C-76-DI-A	Val-C-DI-A	0.91	12.60	3.79
Aradu.I6GSC	LOB Domaincontaining 1	C-76-DI-A	Val-C-DI-A	0.33	4.57	3.81
Aradu.1S9TS	Alphaexpansin 4	C-76-DI-A	Val-C-DI-A	7.84	111.55	3.83
Aradu.QJ19Z	Lactosylceramide 4Alphagalactosyltransferase	C-76-DI-A	Val-C-DI-A	10.55	150.97	3.84
Aradu.L4Z5B	Wat1Related At1G70260	C-76-DI-A	Val-C-DI-A	1.07	15.53	3.85
Aradu.XE5AY	Phosphatidylglycerol Phosphatidylinositol TransferD	C-76-DI-A	Val-C-DI-A	0.23	3.43	3.88
Aradu.H8T23	Gdsl Esterase Lipase Apg	C-76-DI-A	Val-C-DI-A	0.12	1.74	3.88
Aradu.M0QVM	RNase Hi	C-76-DI-A	Val-C-DI-A	0.17	2.61	3.90
Aradu.K2F98	Hypothetical Protein	C-76-DI-A	Val-C-DI-A	0.35	5.31	3.91

Aradu.25DKA	Dnabinding Escarola	C-76-DI-A	Val-C-DI-A	7.03	106.16	3.92
Aradu.38TXW	Lob Domaincontaining 21	C-76-DI-A	Val-C-DI-A	0.61	9.47	3.96
Aradu.5474V	Isoflavone 2 Hydroxylase	C-76-DI-A	Val-C-DI-A	10.40	164.49	3.98
Aradu.JQ0FN	Lob Domaincontaining 4	C-76-DI-A	Val-C-DI-A	0.61	9.63	3.98
Aradu.Q2835	Chalcone Synthase	C-76-DI-A	Val-C-DI-A	0.68	10.93	4.00
Aradu.4118A	Asparticase	C-76-DI-A	Val-C-DI-A	0.08	1.26	4.01
Aradu.Y6LUX	Nac Domaincontaining 100	C-76-DI-A	Val-C-DI-A	2.19	35.74	4.03
Aradu.U1NA9	Zinc Finger Ccch Domaincontaining 39	C-76-DI-A	Val-C-DI-A	0.38	6.34	4.06
Aradu.IY25U	Mitochondrial Phosphate Carrier Mitochondrial	C-76-DI-A	Val-C-DI-A	0.76	12.89	4.09
Aradu.UP79J	Probable Wrky Transcription Factor 75	C-76-DI-A	Val-C-DI-A	1.25	21.59	4.11
Aradu.A834G	Hypothetical Protein Phavu_009G079000G	C-76-DI-A	Val-C-DI-A	0.33	5.66	4.11
Aradu.E2KJV	Auxininduced 22E	C-76-DI-A	Val-C-DI-A	0.69	12.01	4.13
Aradu.Y1LYG	Pathogenesisrelated Pr1	C-76-DI-A	Val-C-DI-A	1.30	22.76	4.13
Aradu.FW9SW	Lysine Ketoglutarate Reductase Transsplicing	C-76-DI-A	Val-C-DI-A	0.16	2.79	4.14
Aradu.UN18Y	Peroxisome Biogenesis 7	C-76-DI-A	Val-C-DI-A	0.36	6.38	4.14
Aradu.Q86YJ	Udpglucosyl Transferase Isoform 1	C-76-DI-A	Val-C-DI-A	1.38	24.65	4.16
Aradu.Z9DZQ	Uncharacterized Protein Loc100779434	C-76-DI-A	Val-C-DI-A	0.11	1.93	4.18
Aradu.CAT7T	Kiwellin	C-76-DI-A	Val-C-DI-A	0.06	1.13	4.21
Aradu.6A07N	Flavonol Synthase Flavanone 3Hydroxylase	C-76-DI-A	Val-C-DI-A	0.90	17.02	4.24
Aradu.K3ZSF	Hypothetical Protein Glysoja_040812	C-76-DI-A	Val-C-DI-A	1.25	23.64	4.25
Aradu.GS29Q	Pathogenesisrelated Class Partial	C-76-DI-A	Val-C-DI-A	4.83	91.96	4.25
Aradu.G5NTR	Uncharacterized Protein Loc101255289	C-76-DI-A	Val-C-DI-A	0.21	4.19	4.29
Aradu.LE9UW	Duf538 Family	C-76-DI-A	Val-C-DI-A	2.87	58.16	4.34
Aradu.XGK7G	Plastocyanin Domain	C-76-DI-A	Val-C-DI-A	0.15	3.30	4.43
Aradu.XCA3M	Aquaporin Tip21	C-76-DI-A	Val-C-DI-A	14.81	321.10	4.44
Aradu.9C9ZX	Disease Resistance Response	C-76-DI-A	Val-C-DI-A	4.60	100.50	4.45
Aradu.NHB6E	Intracellular Chloride Channel	C-76-DI-A	Val-C-DI-A	0.11	2.43	4.45
Aradu.RDQ06	Late Embryogenesis Abundant Hydroxyprolinerich	C-76-DI-A	Val-C-DI-A	21.65	516.14	4.58
Aradu.CZ6KH	Cytochrome P450 71A9	C-76-DI-A	Val-C-DI-A	0.31	7.54	4.59
Aradu.TMM2A	Nonspecific Lipidtransfer 4	C-76-DI-A	Val-C-DI-A	0.57	13.81	4.60
Aradu.ZN3FN	Disease Resistance Rpp13 1	C-76-DI-A	Val-C-DI-A	0.15	3.59	4.62
Aradu.AA5UH	Dentin Sialophospho Isoform 3	C-76-DI-A	Val-C-DI-A	0.23	5.73	4.64
Aradu.FDB38	Peroxidase 15	C-76-DI-A	Val-C-DI-A	1.45	36.77	4.66

Aradu.B20QU	Woundinduced 1 Partial	C-76-DI-A	Val-C-DI-A	5.69	147.76	4.70
Aradu.TQ01E	Uncharacterized Protein Loc100527541	C-76-DI-A	Val-C-DI-A	0.35	9.11	4.70
Aradu.HZI51	Zinc Finger Zat4	C-76-DI-A	Val-C-DI-A	0.05	1.49	4.80
Aradu.Z2UCJ	Probable Receptor Kinase At1G67000 Isoform X1	C-76-DI-A	Val-C-DI-A	0.40	11.72	4.86
Aradu.1T3UD	Chitinase 2	C-76-DI-A	Val-C-DI-A	1.32	38.67	4.87
Aradu.U5008	Auxininduced 5Ng4	C-76-DI-A	Val-C-DI-A	0.12	3.64	4.92
Aradu.GX5W4	Disease Resistance Response 206	C-76-DI-A	Val-C-DI-A	0.26	7.94	4.96
Aradu.I2J7R	Linoleate 13Slipoxygenase 2 Chloroplastic	C-76-DI-A	Val-C-DI-A	1.79	55.76	4.96
Aradu.BX6BE	LRR Receptor Serine Threonine Tyrosine Kinase	C-76-DI-A	Val-C-DI-A	0.33	10.92	5.05
Aradu.WSW8I	Sbp (Sribonucleasebinding) Family	C-76-DI-A	Val-C-DI-A	0.96	32.51	5.09
Aradu.STH91	Alphaamylase	C-76-DI-A	Val-C-DI-A	0.20	6.97	5.11
Aradu.KD2FK	Phenazine Biosynthesis Family	C-76-DI-A	Val-C-DI-A	0.57	19.67	5.12
Aradu.H4BH8	Alpha Tubulin 1	C-76-DI-A	Val-C-DI-A	0.33	11.71	5.13
Aradu.L3677	Lysine Histidine Transporter 1	C-76-DI-A	Val-C-DI-A	0.31	11.44	5.21
Aradu.VE705	Probable Wrky Transcription Factor 51	C-76-DI-A	Val-C-DI-A	1.05	39.27	5.22
Aradu.65WG6	Disease Resistance Rpp13 1 Isoform X1	C-76-DI-A	Val-C-DI-A	0.13	4.68	5.23
Aradu.ZD9KZ	Heat Shock Factor Hsf30	C-76-DI-A	Val-C-DI-A	0.47	19.17	5.36
Aradu.N5BYB	Plant F18G18200	C-76-DI-A	Val-C-DI-A	0.10	4.38	5.51
Aradu.8Q6IV	Madsbox Soc1	C-76-DI-A	Val-C-DI-A	0.29	13.53	5.55
Aradu.D14Q2	Probable Carboxylesterase 15	C-76-DI-A	Val-C-DI-A	9.91	471.28	5.57
Aradu.Q3KFX	Probable Arabinose 5Phosphate Isomerase	C-76-DI-A	Val-C-DI-A	0.23	10.98	5.57
Aradu.4E997	Pgr5 Chloroplastic	C-76-DI-A	Val-C-DI-A	1.02	50.55	5.62
Aradu.WLE0A	Gdsl Esterase Lipase	C-76-DI-A	Val-C-DI-A	0.38	24.01	5.96
Aradu.G737E	Ribonuclease 3	C-76-DI-A	Val-C-DI-A	0.68	54.31	6.31
Aradu.4V2X0	10 Kda Chaperonin	C-76-DI-A	Val-C-DI-A	0.83	69.54	6.38
Aradu.ZFE21	Lectin	C-76-DI-A	Val-C-DI-A	0.16	13.88	6.43
Aradu.P4T20	CASP Poptrdraft_823430	C-76-DI-A	Val-C-DI-A	4.23	370.66	6.45
Aradu.WC0YR	Sigma Factor Binding Chloroplastic	C-76-DI-A	Val-C-DI-A	0.32	107.11	8.40
Aradu.EGH8I	Duf4408 Domain	C-76-DI-A	Val-C-DI-A	0.02	15.37	9.50
Araip.PNG34	Nucleoporin Nup188 Homolog Isoform X1	C-76-DI-B	Val-C-DI-B	22.58	0.03	-9.40
Araip.HC3YR	Atpdependent Dna Helicase Pif1	C-76-DI-B	Val-C-DI-B	10.07	0.07	-7.22
Araip.RU4HV	Uncharacterized Protein Loc101503342	C-76-DI-B	Val-C-DI-B	50.52	0.52	-6.61
Araip.UPC07	Probable Polygalacturonase	C-76-DI-B	Val-C-DI-B	4.18	0.06	-6.04

Araip.WZN7R	Sieve Element Occlusion C	C-76-DI-B	Val-C-DI-B	6.99	0.11	-5.98
Araip.GI4T0	Probable Isoleucinetrna Cytoplasmic	C-76-DI-B	Val-C-DI-B	8.28	0.13	-5.96
Araip.DTE4E	Serine Threonine Kinase Ht1 Isoform X3	C-76-DI-B	Val-C-DI-B	23.76	0.39	-5.93
Araip.0HT7P	Uncharacterized Protein Loc101255289	C-76-DI-B	Val-C-DI-B	6.14	0.11	-5.83
Araip.W2QM3	Chromosomeassociated Kinesin Kif4A	C-76-DI-B	Val-C-DI-B	24.32	0.47	-5.71
Araip.ED0ID	Partial	C-76-DI-B	Val-C-DI-B	12.81	0.28	-5.51
Araip.IW8UC	Bromodomaincontaining Factor 1 Isoform X2	C-76-DI-B	Val-C-DI-B	22.06	0.58	-5.25
Araip.S2BMK	Vacuolarsorting Receptor 1	C-76-DI-B	Val-C-DI-B	6.82	0.19	-5.15
Araip.98VT4	Transcription Factor Bhlh90	C-76-DI-B	Val-C-DI-B	9.43	0.28	-5.07
Araip.QR2N6	Poly(Rc)Binding 1	C-76-DI-B	Val-C-DI-B	6.37	0.20	-4.97
Araip.PET3Z	Hgwp Repeat Containing	C-76-DI-B	Val-C-DI-B	2.56	0.08	-4.95
Araip.A8NDA	Disease Resistance	C-76-DI-B	Val-C-DI-B	0.40	0.01	-4.88
Araip.U71AB	Disease Resistance (Ccnbslrr Class) Family	C-76-DI-B	Val-C-DI-B	13.50	0.48	-4.81
Araip.NY165	K+H+ Exchange	C-76-DI-B	Val-C-DI-B	5.54	0.21	-4.74
Araip.N3U2H	2Isopropylmalate Synthase Chloroplastic	C-76-DI-B	Val-C-DI-B	3.95	0.16	-4.59
Araip.C0SVX	Gtype Lectin Sreceptor Serine Threonine Kinase	C-76-DI-B	Val-C-DI-B	0.60	0.03	-4.55
Araip.A8BYM	Hypothetical Protein B456_002G216700	C-76-DI-B	Val-C-DI-B	13.43	0.58	-4.54
Araip.YJY2H	Coproporphyrinogen Iii Oxidase	C-76-DI-B	Val-C-DI-B	4.51	0.21	-4.45
Araip.13WTY	Syntaxin124 Isoform X2	C-76-DI-B	Val-C-DI-B	12.83	0.61	-4.39
Araip.90JS8	Leucinerich Repeat Receptor Kinase	C-76-DI-B	Val-C-DI-B	13.02	0.67	-4.29
Araip.XLK0H	Probable Leucinerich Repeat Receptor Kinase	C-76-DI-B	Val-C-DI-B	2.13	0.12	-4.17
Araip.8A1NA	Aldose Reductase	C-76-DI-B	Val-C-DI-B	8.91	0.51	-4.13
Araip.0H886	Sadenosyllmethioninedependent Methyltransferases	C-76-DI-B	Val-C-DI-B	4.43	0.26	-4.12
Araip.H9S11	Entcopalyl Diphosphate Chloroplastic	C-76-DI-B	Val-C-DI-B	1.27	0.07	-4.09
Araip.28IXU	Kinase Superfamily Isoform 1	C-76-DI-B	Val-C-DI-B	5.60	0.33	-4.07
Araip.7J81A	Cytochrome P450 78A5	C-76-DI-B	Val-C-DI-B	21.65	1.36	-3.99
Araip.L7IDG	1Aminocyclopropane1Carboxylate Oxidase Homolog	C-76-DI-B	Val-C-DI-B	16.84	1.06	-3.99
Araip.DM9S9	Receptor 12 Isoform X1	C-76-DI-B	Val-C-DI-B	2.56	0.17	-3.91
Araip.6ZX2I	Proline Iminopeptidase	C-76-DI-B	Val-C-DI-B	20.63	1.40	-3.89
Araip.BI3R5	Syntaxin121 Isoform 1	C-76-DI-B	Val-C-DI-B	4.75	0.32	-3.87
Araip.I60BC	Nac Domaincontaining 90	C-76-DI-B	Val-C-DI-B	1.90	0.13	-3.85
Araip.92HTJ	Disease Resistance (Tirnbslrr Class)	C-76-DI-B	Val-C-DI-B	1.84	0.13	-3.85
Araip.VU7AW	Nadpdependent Glyceraldehyde3PDehydrogenase	C-76-DI-B	Val-C-DI-B	5.94	0.44	-3.77

Araip.TH7G5	Gdsl Esterase Lipase At2G03980	C-76-DI-B	Val-C-DI-B	2.15	0.16	-3.76
Araip.C4T4Y	Chromosomeassociated Kinesin Kif4A	C-76-DI-B	Val-C-DI-B	7.47	0.59	-3.65
Araip.982E5	Ubiquitin Ligase	C-76-DI-B	Val-C-DI-B	7.02	0.57	-3.61
Araip.VQ0MQ	Microtubuleassociated 705	C-76-DI-B	Val-C-DI-B	2.68	0.22	-3.61
Araip.51VQE	Sucrose Synthase 6	C-76-DI-B	Val-C-DI-B	3.53	0.30	-3.57
Araip.D4SZE	Ring Finger 141	C-76-DI-B	Val-C-DI-B	6.88	0.59	-3.53
Araip.P6D1W	Phosphatidylinositol:CeramideInositolphosphotransfe	C-76-DI-B	Val-C-DI-B	2.69	0.23	-3.53
Araip.PC9SS	Plant T8M16150	C-76-DI-B	Val-C-DI-B	4.82	0.42	-3.52
Araip.E2PJR	Receptor 12	C-76-DI-B	Val-C-DI-B	31.49	2.87	-3.46
Araip.C1BTX	Regulator Of Nonsense Transcripts Upf3 Isoform X1	C-76-DI-B	Val-C-DI-B	4.14	0.39	-3.42
Araip.M0UQ9	Lalad Lglu Epimerase	C-76-DI-B	Val-C-DI-B	8.08	0.75	-3.42
Araip.NP2VV	Atpase Family Aaa Domaincontaining 1	C-76-DI-B	Val-C-DI-B	1.68	0.16	-3.39
Araip.Y51FS	Cullinassociated Nedd8Dissociated 1	C-76-DI-B	Val-C-DI-B	10.88	1.06	-3.35
Araip.438GZ	Transmembrane 194A	C-76-DI-B	Val-C-DI-B	2.19	0.21	-3.35
Araip.62RED	Clp Proteaserelated Chloroplastic	C-76-DI-B	Val-C-DI-B	2.71	0.27	-3.35
Araip.GYU8U	Deoxyuridine 5 Triphosphate Nucleotidohydrolase	C-76-DI-B	Val-C-DI-B	18.77	1.86	-3.33
Araip.IBW09	Sucrose Synthase	C-76-DI-B	Val-C-DI-B	1.67	0.17	-3.30
Araip.CI0BZ	Phosphoglycerate 2,3Bisphosphoglycerateindependen	C-76-DI-B	Val-C-DI-B	12.97	1.33	-3.29
Araip.GC54R	Metacaspase3 Isoform X1	C-76-DI-B	Val-C-DI-B	1.63	0.17	-3.26
Araip.68YBY	Rj2	C-76-DI-B	Val-C-DI-B	2.27	0.24	-3.22
Araip.75NYJ	Probable Mitochondrial Chaperone Bcs1B	C-76-DI-B	Val-C-DI-B	1.48	0.16	-3.21
Araip.B3EI3	Adpribosylation Factor Gtpaseactivating Agd12	C-76-DI-B	Val-C-DI-B	6.10	0.66	-3.20
Araip.TJ4P5	Nrt1 Ptr Family	C-76-DI-B	Val-C-DI-B	0.99	0.11	-3.19
Araip.30TPF	Cysteine Synthase	C-76-DI-B	Val-C-DI-B	4.99	0.56	-3.17
Araip.IYR09	Atpdependent Rna Helicase Dhx33	C-76-DI-B	Val-C-DI-B	0.66	0.07	-3.17
Araip.CX9DW	Myosinj Heavy Chain	C-76-DI-B	Val-C-DI-B	71.89	8.10	-3.15
Araip.G3UJ6	Endo1,4Betaxylanase A	C-76-DI-B	Val-C-DI-B	1.41	0.16	-3.13
Araip.77JRH	Acetylcoenzyme A Carboxylase Carboxyl Transferase	C-76-DI-B	Val-C-DI-B	1.92	0.22	-3.12
Araip.9ZC59	Abscisic Acid 8 Hydroxylase 1	C-76-DI-B	Val-C-DI-B	4.46	0.52	-3.11
Araip.J28NT	Photosystem Ii D1 (Chloroplast)	C-76-DI-B	Val-C-DI-B	16.98	1.98	-3.10
Araip.YA0CB	Uncharacterized Aarf Domaincontaining Kinase	C-76-DI-B	Val-C-DI-B	1.99	0.23	-3.10
Araip.RM081	Transcription Factor Bhlh61	C-76-DI-B	Val-C-DI-B	23.42	2.76	-3.08
Araip.3DN9J	Dna Rnabinding Kin17	C-76-DI-B	Val-C-DI-B	7.57	0.94	-3.01

Araip.DN365	P21 Isoform X1	C-76-DI-B	Val-C-DI-B	6.30	50.78	3.01
Araip.NUE3Q	Xylem Cysteine Ase 2	C-76-DI-B	Val-C-DI-B	0.59	4.84	3.03
Araip.GZ59D	Acyl Thioesterase 2	C-76-DI-B	Val-C-DI-B	2.48	20.40	3.04
Araip.CCW4K	Em Gea6	C-76-DI-B	Val-C-DI-B	9.81	80.81	3.04
Araip.D98D3	Ureide Permease 1 Isoform X1	C-76-DI-B	Val-C-DI-B	3.48	28.87	3.05
Araip.GNL1S	Lightdependent Short Hypocotyls 10	C-76-DI-B	Val-C-DI-B	1.28	10.67	3.06
Araip.L1EC6	Udpglycosyltransferase 87A1	C-76-DI-B	Val-C-DI-B	1.23	10.23	3.06
Araip.GZF1H	Subtilisin Protease	C-76-DI-B	Val-C-DI-B	0.18	1.54	3.07
Araip.K1FJE	Peroxidase 4	C-76-DI-B	Val-C-DI-B	3.96	33.26	3.07
Araip.KAF3M	Dnadamagerepair Toleration Drt100	C-76-DI-B	Val-C-DI-B	0.25	2.09	3.07
Araip.E4AWE	Transmembrane Protein,	C-76-DI-B	Val-C-DI-B	9.72	83.63	3.11
Araip.WHP10	Par1	C-76-DI-B	Val-C-DI-B	3.16	27.16	3.11
Araip.4IT0A	Isoflavone 7Omethyltransferase	C-76-DI-B	Val-C-DI-B	0.28	2.39	3.11
Araip.R4F7W	Mitochondrial Inner Membrane Protease Subunit 1	C-76-DI-B	Val-C-DI-B	0.51	4.41	3.11
Araip.S6YA0	Purple Acid Phosphatase 17	C-76-DI-B	Val-C-DI-B	1.93	16.64	3.11
Araip.06TZY	Lysosomal Prox Carboxypeptidase	C-76-DI-B	Val-C-DI-B	0.87	7.49	3.11
Araip.LL05Y	Fbox Rni Fbd Domain	C-76-DI-B	Val-C-DI-B	0.76	6.56	3.12
Araip.RA8PB	Ethyleneresponsive Transcription Factor Erf023	C-76-DI-B	Val-C-DI-B	3.33	29.15	3.13
Araip.GUE86	Probable Lrr Receptor Serine Threonine Kinase	C-76-DI-B	Val-C-DI-B	0.25	2.23	3.14
Araip.E3E4E	Caffeic Acid 3Omethyltransferase	C-76-DI-B	Val-C-DI-B	1.32	11.73	3.15
Araip.R7MQK	Lactosylceramide 4Alphagalactosyltransferase	C-76-DI-B	Val-C-DI-B	0.33	2.90	3.15
Araip.5W15W	Ploop Nucleoside Triphosphate Hydrolase Superfami	C-76-DI-B	Val-C-DI-B	0.98	8.71	3.16
Araip.2B206	Receptor Kinase	C-76-DI-B	Val-C-DI-B	0.14	1.22	3.16
Araip.VCE11	Sieve Element Occlusion	C-76-DI-B	Val-C-DI-B	4.27	38.20	3.16
Araip.G3WHQ	Pra1 Family	C-76-DI-B	Val-C-DI-B	1.30	11.66	3.16
Araip.YA4KW	Phosphatidylglycerol Phosphatidylinositol TransferD	C-76-DI-B	Val-C-DI-B	1.16	10.43	3.16
Araip.R2FTY	Glutathione S Aminoterminal Domain	C-76-DI-B	Val-C-DI-B	1.94	17.38	3.16
Araip.1K5GM	Coumaroyl:Anthocyanidin3Ogluco6 maroyltransferas	C-76-DI-B	Val-C-DI-B	0.13	1.16	3.16
Araip.M07D2	Tetraspanin Family	C-76-DI-B	Val-C-DI-B	0.86	7.77	3.17
Araip.9Y1W2	Unknown	C-76-DI-B	Val-C-DI-B	2.27	20.45	3.17
Araip.43WXS	Amidase	C-76-DI-B	Val-C-DI-B	0.21	1.91	3.17
Araip.97TWR	Notum Homolog	C-76-DI-B	Val-C-DI-B	0.75	6.77	3.17
Araip.M9LG8	Ringh2 Finger Atl22	C-76-DI-B	Val-C-DI-B	0.99	9.01	3.18

Araip.TK3NZ	3Dehydroquinate Dehydratase Shikimate Chloroplast	C-76-DI-B	Val-C-DI-B	2.13	19.59	3.20
Araip.IQ2QT	Uncharacterized Acetyltransferase At3G50280	C-76-DI-B	Val-C-DI-B	0.74	6.81	3.20
Araip.Y9DP8	DNA Mismatch Repair Msh3 Isoform X1	C-76-DI-B	Val-C-DI-B	1.75	16.16	3.20
Araip.KRG2J	Serine Threonine Phosphatase 7 Long Form Homolog	C-76-DI-B	Val-C-DI-B	0.09	0.87	3.21
Araip.BH9LK	2Ogfe(Ii) Oxygenase Family Oxidoreductase	C-76-DI-B	Val-C-DI-B	1.91	17.87	3.22
Araip.849ER	Linoleate 13Slipoxygenase 3 Chloroplastic	C-76-DI-B	Val-C-DI-B	0.06	0.52	3.22
Araip.KTN2S	Nonsymbiotic Hemoglobin	C-76-DI-B	Val-C-DI-B	0.28	2.63	3.23
Araip.5UF8N	LRR Receptor Serine Threonine Kinase Gso2	C-76-DI-B	Val-C-DI-B	0.65	6.10	3.24
Araip.I49B9	Nbslrr Resistance	C-76-DI-B	Val-C-DI-B	0.41	3.86	3.24
Araip.P8QST	Homogentisate Phytylprenyltransferase	C-76-DI-B	Val-C-DI-B	0.36	3.39	3.24
Araip.V205R	Allergen Pru	C-76-DI-B	Val-C-DI-B	5.51	52.35	3.25
Araip.U23Q6	Patatin 7	C-76-DI-B	Val-C-DI-B	0.32	3.10	3.25
Araip.UC7G4	Calmodulinbinding Isoform Partial	C-76-DI-B	Val-C-DI-B	0.13	1.21	3.25
Araip.BXG5M	Udprhamnose:Rhamnosyltransferase 1	C-76-DI-B	Val-C-DI-B	1.66	16.03	3.27
Araip.ZX8HU	Stemspecific Tsjt1	C-76-DI-B	Val-C-DI-B	35.84	345.36	3.27
Araip.KF0IU	Flavonol Synthase Flavanone 3Hydroxylase	C-76-DI-B	Val-C-DI-B	0.17	1.63	3.27
Araip.ZS1T2	Fbox At1G61340	C-76-DI-B	Val-C-DI-B	0.39	3.81	3.27
Araip.20CID	Chitinase (Class Ib) Hevein	C-76-DI-B	Val-C-DI-B	0.76	7.36	3.28
Araip.BS7A3	Hypersensitiveinduced Response 2	C-76-DI-B	Val-C-DI-B	1.03	9.98	3.28
Araip.C5RH9	Casp Aralydraft_485429	C-76-DI-B	Val-C-DI-B	1.72	16.76	3.28
Araip.342YB	Kunitztype Trypsin Inhibitor 2	C-76-DI-B	Val-C-DI-B	2.80	27.40	3.29
Araip.XVL9X	Auxininduced Aux22	C-76-DI-B	Val-C-DI-B	0.87	8.56	3.29
Araip.BH5Q5	Isoflavone 2 Hydroxylase	C-76-DI-B	Val-C-DI-B	0.09	0.90	3.32
Araip.YM0NV	Nuclearpore Anchor	C-76-DI-B	Val-C-DI-B	0.06	0.64	3.34
Araip.KF4YH	Transmembrane Protein,	C-76-DI-B	Val-C-DI-B	0.72	7.30	3.35
Araip.XK9AB	Nac Domaincontaining 74	C-76-DI-B	Val-C-DI-B	0.18	1.86	3.35
Araip.X5DDA	Heavy Metal Transport Detoxification Superfamily	C-76-DI-B	Val-C-DI-B	0.62	6.29	3.35
Araip.64TPH	Glucan Endo1,3Betaglucosidase	C-76-DI-B	Val-C-DI-B	0.31	3.14	3.36
Araip.I5KPB	Probable Inorganic Phosphate Transporter 19	C-76-DI-B	Val-C-DI-B	5.16	53.00	3.36
Araip.CI752	Cyclin D31	C-76-DI-B	Val-C-DI-B	0.40	4.11	3.36
Araip.L3Q4J	Anthocyanidin Synthase	C-76-DI-B	Val-C-DI-B	0.25	2.55	3.37
Araip.T873S	Ripening Related Family	C-76-DI-B	Val-C-DI-B	5.64	58.83	3.38
Araip.89U7Q	Alphaamylase	C-76-DI-B	Val-C-DI-B	0.61	6.38	3.39

Araip.QZA57	Expansin A10	C-76-DI-B	Val-C-DI-B	1.76	18.55	3.40
Araip.30QH3	ABC Transporter C Family Member 10	C-76-DI-B	Val-C-DI-B	0.16	1.70	3.40
Araip.5HK6K	Glutathione Stransferase	C-76-DI-B	Val-C-DI-B	0.72	7.64	3.41
Araip.WT898	LRR Receptor Serine Threonine Kinase Fls2	C-76-DI-B	Val-C-DI-B	0.42	4.51	3.41
Araip.V9JLA	Zip Zinc Iron Transport Family	C-76-DI-B	Val-C-DI-B	27.95	298.07	3.41
Araip.E29HH	Phospholipase A(1) Lcat3	C-76-DI-B	Val-C-DI-B	0.26	2.76	3.42
Araip.JPJ83	Pathogenesisrelated Class 10	C-76-DI-B	Val-C-DI-B	0.40	4.28	3.42
Araip.LL42G	Bcell Lymphoma 6	C-76-DI-B	Val-C-DI-B	1.45	15.72	3.44
Araip.K8QIE	Desiccationrelated Pcc306	C-76-DI-B	Val-C-DI-B	0.47	5.16	3.45
Araip.DV8IK	Blue Copper	C-76-DI-B	Val-C-DI-B	0.32	3.56	3.46
Araip.GN3MY	Plant F18G18200	C-76-DI-B	Val-C-DI-B	0.43	4.73	3.46
Araip.V8R8N	2Aminoethanethiol Dioxygenase	C-76-DI-B	Val-C-DI-B	0.08	0.89	3.46
Araip.Z7H22	Madsbox Transcription Factor 23 Isoform X1	C-76-DI-B	Val-C-DI-B	0.26	2.87	3.47
Araip.19RI9	2Aminoethanethiol Dioxygenase	C-76-DI-B	Val-C-DI-B	0.66	7.38	3.48
Araip.SJ708	Serine Threonine Kinase Sapk2	C-76-DI-B	Val-C-DI-B	0.68	7.62	3.49
Araip.23PZD	Coiledcoil Domaincontaining 132	C-76-DI-B	Val-C-DI-B	0.09	1.05	3.49
Araip.Y9MKL	Tau Class Glutathione Stransferase	C-76-DI-B	Val-C-DI-B	2.24	25.14	3.49
Araip.EZ9B8	Derlin	C-76-DI-B	Val-C-DI-B	2.01	22.59	3.49
Araip.JU6JI	Probable LRR Receptor Serine Threonine Kinase	C-76-DI-B	Val-C-DI-B	0.11	1.23	3.49
Araip.UU6MU	Auxinresponsive Iaa29	C-76-DI-B	Val-C-DI-B	0.45	5.08	3.50
Araip.E30MW	Expansin 2	C-76-DI-B	Val-C-DI-B	2.13	24.26	3.51
Araip.6EI1C	Tpr Repeat	C-76-DI-B	Val-C-DI-B	0.11	1.25	3.51
Araip.AH1BZ	Probable Glutathione Stransferase	C-76-DI-B	Val-C-DI-B	2.85	32.71	3.52
Araip.DQ8EI	Branchedchainaminoacid Aminotransferase	C-76-DI-B	Val-C-DI-B	4.38	50.30	3.52
Araip.5M5DL	Ralf 34	C-76-DI-B	Val-C-DI-B	0.45	5.22	3.55
Araip.FVS81	Uncharacterized Protein Loc101499882	C-76-DI-B	Val-C-DI-B	2.39	28.11	3.56
Araip.B7MSZ	Salt Overly Sensitive 1B Isoform 1	C-76-DI-B	Val-C-DI-B	0.37	4.33	3.56
Araip.I0HKJ	Xyloglucan Endotransglucosylase Hydrolase 32	C-76-DI-B	Val-C-DI-B	11.60	137.54	3.57
Araip.VWS8G	Pathogenesisrelated 1	C-76-DI-B	Val-C-DI-B	6.65	79.79	3.59
Araip.213GN	Defensin	C-76-DI-B	Val-C-DI-B	18.04	216.64	3.59
Araip.RV92P	Mitochondrial Phosphate Carrier Mitochondrial	C-76-DI-B	Val-C-DI-B	0.18	2.14	3.59
Araip.KI88P	Hypothetical Protein Mtr_4G068220	C-76-DI-B	Val-C-DI-B	1.65	19.95	3.59
Araip.T1I8I	Uncharacterized Protein Loc101507125 Isoform X2	C-76-DI-B	Val-C-DI-B	0.63	7.58	3.60

Araip.20T4P	Udpglycosyltransferase 83A1	C-76-DI-B	Val-C-DI-B	0.23	2.84	3.60
Araip.35VAB	Uncharacterized Membrane At1G06890	C-76-DI-B	Val-C-DI-B	0.58	7.02	3.61
Araip.D3HLX	Kdeltailed Cysteine Endopeptidase Cep1	C-76-DI-B	Val-C-DI-B	2.03	24.99	3.62
Araip.6K7BL	Probable LRR Receptor Serine Threonine Kinase	C-76-DI-B	Val-C-DI-B	0.25	3.11	3.63
Araip.09YU8	Isoflavone 7Omethyltransferase	C-76-DI-B	Val-C-DI-B	0.19	2.33	3.63
Araip.E4KR5	Isoflavone Reductase	C-76-DI-B	Val-C-DI-B	1.46	18.12	3.63
Araip.D59WV	Ammonium Transporter 3 Member 1	C-76-DI-B	Val-C-DI-B	1.10	13.75	3.65
Araip.GC9VN	Pectate Lyase	C-76-DI-B	Val-C-DI-B	0.84	10.56	3.65
Araip.RC9AW	Nbslrr Type Disease Resistance	C-76-DI-B	Val-C-DI-B	0.38	4.81	3.67
Araip.AVV74	Nac Domaincontaining 100	C-76-DI-B	Val-C-DI-B	0.37	4.72	3.67
Araip.AK95Y	Bifunctional Dihydroflavonol Flavanone 4Reductase	C-76-DI-B	Val-C-DI-B	0.94	12.09	3.69
Araip.197DB	Lrr Receptor Serine Threonine Kinase Gso1	C-76-DI-B	Val-C-DI-B	0.22	2.90	3.70
Araip.VEV8X	Pathogenesisrelated 1	C-76-DI-B	Val-C-DI-B	0.24	3.06	3.70
Araip.T3D3V	Pathogenesisrelated Genes Transcriptional Activator	C-76-DI-B	Val-C-DI-B	6.51	85.07	3.71
Araip.3J520	Duf538 Family	C-76-DI-B	Val-C-DI-B	0.79	10.40	3.71
Araip.KIA1S	Aspartic Ase Asp1	C-76-DI-B	Val-C-DI-B	0.29	3.85	3.73
Araip.JR3SU	Unknown	C-76-DI-B	Val-C-DI-B	5.14	68.24	3.73
Araip.8C3IU	Chitinase Family	C-76-DI-B	Val-C-DI-B	0.10	1.38	3.74
Araip.9WJ7K	Nhl Domaincontaining Isoform 1	C-76-DI-B	Val-C-DI-B	0.98	13.05	3.74
Araip.XGB85	Caffeoyl Omethyltransferase	C-76-DI-B	Val-C-DI-B	1.16	15.43	3.74
Araip.LC867	Iaaamino Acid Hydrolase Ilr1 4	C-76-DI-B	Val-C-DI-B	0.31	4.15	3.75
Araip.BG5YS	Btb Poz Domaincontaining At3G49900	C-76-DI-B	Val-C-DI-B	0.47	6.40	3.75
Araip.S11GW	Lactoylglutathione Lyase	C-76-DI-B	Val-C-DI-B	0.65	9.03	3.79
Araip.T9454	Kda Class II Heat Shock	C-76-DI-B	Val-C-DI-B	4.21	58.18	3.79
Araip.GD0W9	Bromodomaincontaining Ddb_G0271118	C-76-DI-B	Val-C-DI-B	0.25	3.43	3.81
Araip.9P0YM	Wrky Transcription Factor	C-76-DI-B	Val-C-DI-B	7.01	99.06	3.82
Araip.6K01Z	Isoflavone7Omethyltransferase	C-76-DI-B	Val-C-DI-B	0.12	1.68	3.83
Araip.Z4ATC	Nonspecific Lipidtransfer 4	C-76-DI-B	Val-C-DI-B	8.60	122.81	3.84
Araip.V48DY	Allene Oxide Cyclase Chloroplastic	C-76-DI-B	Val-C-DI-B	0.25	3.58	3.84
Araip.IN97X	Fbox Pp2B15	C-76-DI-B	Val-C-DI-B	0.19	2.81	3.85
Araip.RB0W1	Lysine Histidine Transporter 1	C-76-DI-B	Val-C-DI-B	0.33	4.78	3.87
Araip.YB61P	Uncharacterized Protein Loc101506875	C-76-DI-B	Val-C-DI-B	1.01	14.96	3.89
Araip.CP7MF	Glucan Endo1,3Betaglucosidase	C-76-DI-B	Val-C-DI-B	7.54	113.44	3.91

Araip.84L6B	Late Embryogenesis Abundant Group 3	C-76-DI-B	Val-C-DI-B	3.89	59.21	3.93
Araip.BA4XW	Probable Nucleoredoxin 1	C-76-DI-B	Val-C-DI-B	4.82	74.82	3.96
Araip.AWV0M	Lrr Receptor Serine Threonine Kinase Fls2	C-76-DI-B	Val-C-DI-B	0.28	4.39	3.97
Araip.RXA31	Expansina8 Precursor	C-76-DI-B	Val-C-DI-B	1.59	25.02	3.97
Araip.8ZE1Y	Pathogenesisrelated Bet V I Family	C-76-DI-B	Val-C-DI-B	1.81	28.70	3.98
Araip.WUT0T	3Isopropylmalate Dehydratase Large Subunit	C-76-DI-B	Val-C-DI-B	0.17	2.72	3.99
Araip.X7Y0N	Plant Mnj717	C-76-DI-B	Val-C-DI-B	0.45	7.23	4.00
Araip.G3NMG	Wuschelrelated Homeobox 5	C-76-DI-B	Val-C-DI-B	0.79	13.09	4.04
Araip.I4NIK	Chitinase 2	C-76-DI-B	Val-C-DI-B	0.56	9.24	4.05
Araip.X6A1T	Alphaxylosidase 1	C-76-DI-B	Val-C-DI-B	0.08	1.33	4.06
Araip.9DW4T	Choline Transporter 2	C-76-DI-B	Val-C-DI-B	0.27	4.51	4.07
Araip.M1W05	Probable Nucleoredoxin 1	C-76-DI-B	Val-C-DI-B	0.65	11.20	4.11
Araip.4F9Q5	Glutathione Stransferase	C-76-DI-B	Val-C-DI-B	0.64	11.12	4.13
Araip.EZV82	Cblinteracting Serine Threonine Kinase 8 Isoform X1	C-76-DI-B	Val-C-DI-B	1.24	21.58	4.13
Araip.V6GGV	Profilin 1	C-76-DI-B	Val-C-DI-B	0.47	8.20	4.13
Araip.UJR3L	Zinc Finger Zat11	C-76-DI-B	Val-C-DI-B	1.10	19.27	4.14
Araip.QE51A	Bifunctional Aspartate Aminotransferase	C-76-DI-B	Val-C-DI-B	0.21	3.65	4.14
Araip.2V924	F0 V0 Subunit C	C-76-DI-B	Val-C-DI-B	1.78	31.79	4.16
Araip.M7BCB	Lrr Receptor Kinase	C-76-DI-B	Val-C-DI-B	0.14	2.49	4.16
Araip.I5AXB	Acetyltransferase (Gnat) Domain	C-76-DI-B	Val-C-DI-B	0.33	6.28	4.23
Araip.68F0J	Srg1	C-76-DI-B	Val-C-DI-B	0.10	1.80	4.23
Araip.2E3EC	Glucan Endo1,3Betaglucosidase	C-76-DI-B	Val-C-DI-B	4.89	92.67	4.25
Araip.Y4XX5	Hypothetical Protein Glysoja_021291	C-76-DI-B	Val-C-DI-B	2.82	54.00	4.26
Araip.RQ18Z	N(6)Adeninespecific Dna Methyltransferase 2	C-76-DI-B	Val-C-DI-B	0.14	2.74	4.28
Araip.G2UJA	Probable Wrky Transcription Factor 50	C-76-DI-B	Val-C-DI-B	9.15	180.21	4.30
Araip.SK1X8	Trichome Birefringence 33	C-76-DI-B	Val-C-DI-B	0.10	2.07	4.35
Araip.18JNS	Pectinesterase Inhibitor	C-76-DI-B	Val-C-DI-B	0.43	8.87	4.36
Araip.Q6KXG	Eg45 Domain Containing	C-76-DI-B	Val-C-DI-B	3.87	79.41	4.36
Araip.T5KFI	Vacuolar Sortingassociated 35A	C-76-DI-B	Val-C-DI-B	1.75	35.88	4.36
Araip.77QS1	Wallassociated Receptor Kinase	C-76-DI-B	Val-C-DI-B	0.20	4.17	4.38
Araip.31ZB6	Alphaamylase Subtilisin Inhibitor	C-76-DI-B	Val-C-DI-B	4.90	104.21	4.41
Araip.Y41TM	Beta1,4Xylosyltransferase Irx9	C-76-DI-B	Val-C-DI-B	0.14	2.98	4.41
Araip.NPQ3A	Uncharacterized Protein Loc104215674	C-76-DI-B	Val-C-DI-B	0.07	1.60	4.47

Araip.ZTP1V	Subtilisin Inhibitor 1	C-76-DI-B	Val-C-DI-B	1.05	23.39	4.47
Araip.J0W46	Xylem Cysteine Ase 1	C-76-DI-B	Val-C-DI-B	17.37	389.89	4.49
Araip.BF1SB	Probable Calciumbinding Cml41	C-76-DI-B	Val-C-DI-B	0.64	14.63	4.50
Araip.UY6ZE	Pathogenesisrelated Pr4	C-76-DI-B	Val-C-DI-B	20.48	469.54	4.52
Araip.11VE0	Kda Prolinerich	C-76-DI-B	Val-C-DI-B	0.32	7.49	4.55
Araip.BPR1L	Nonspecific Lipidtransfer 1	C-76-DI-B	Val-C-DI-B	6.54	158.03	4.60
Araip.2H071	Xyloglucan Endotransglucosylase Hydrolase	C-76-DI-B	Val-C-DI-B	0.21	5.09	4.60
Araip.67HK3	Glycinerich Rnabinding Mitochondrial	C-76-DI-B	Val-C-DI-B	0.45	11.01	4.60
Araip.HV7J0	Probable Acylactivating Enzyme Peroxisomal	C-76-DI-B	Val-C-DI-B	0.09	2.17	4.61
Araip.I128H	Flavonol Synthase Flavanone 3Hydroxylase	C-76-DI-B	Val-C-DI-B	0.15	3.66	4.61
Araip.PS98H	Trichome Birefringence 23	C-76-DI-B	Val-C-DI-B	0.18	4.65	4.66
Araip.983RG	Glycoside Hydrolase Family 18	C-76-DI-B	Val-C-DI-B	1.58	41.38	4.71
Araip.K1GCE	Swi Snf Complex Subunit Swi3B	C-76-DI-B	Val-C-DI-B	0.59	16.29	4.80
Araip.QZ06Q	Miraculin	C-76-DI-B	Val-C-DI-B	0.39	11.08	4.81
Araip.4G0SR	Polyphenol Oxidase Chloroplastic	C-76-DI-B	Val-C-DI-B	0.09	2.48	4.84
Araip.JK92J	Fbox Plant	C-76-DI-B	Val-C-DI-B	0.04	1.23	4.84
Araip.TUE3L	E3 Ubiquitin Ligase Ring1	C-76-DI-B	Val-C-DI-B	0.13	3.81	4.85
Araip.RJJ5V	Lob Domaincontaining 4	C-76-DI-B	Val-C-DI-B	1.63	49.06	4.91
Araip.TE1P2	Transcription Factor Bhlh18	C-76-DI-B	Val-C-DI-B	0.18	5.45	4.91
Araip.WHJ14	Wrky Transcription Factor 40	C-76-DI-B	Val-C-DI-B	1.14	35.05	4.94
Araip.54UE2	Da1Related 2	C-76-DI-B	Val-C-DI-B	0.22	6.81	4.96
Araip.2N7ZX	Elongation Of Fatty Acids 3	C-76-DI-B	Val-C-DI-B	0.23	7.89	5.10
Araip.20FU5	Probable Wrky Transcription Factor 40	C-76-DI-B	Val-C-DI-B	0.60	20.54	5.10
Araip.56E0J	Allergen Pru	C-76-DI-B	Val-C-DI-B	0.38	13.20	5.13
Araip.NFP9Z	Kda Prolinerich	C-76-DI-B	Val-C-DI-B	1.36	49.62	5.19
Araip.13WAT	Hxxxdtype Acyltransferase Family	C-76-DI-B	Val-C-DI-B	0.36	13.32	5.22
Araip.W4FZ1	Phosphatidate Phosphatase Lpin2 Isoform X1	C-76-DI-B	Val-C-DI-B	0.30	11.44	5.26
Araip.EXQ89	Gdsl Esterase Lipase Apg	C-76-DI-B	Val-C-DI-B	0.30	11.71	5.30
Araip.WY6XF	Heavy Metalassociated Isoprenylated Plant 26	C-76-DI-B	Val-C-DI-B	0.27	10.90	5.31
Araip.Q2JPR	Bon1Associated 2	C-76-DI-B	Val-C-DI-B	0.18	7.45	5.35
Araip.0Y5YA	Gcn5Related Nacetyltransferase Family	C-76-DI-B	Val-C-DI-B	0.18	7.40	5.36
Araip.SCU5A	Nuclear Export Mediator Factor Nemf	C-76-DI-B	Val-C-DI-B	0.81	33.74	5.39
Araip.5T1RR	Expansin Alpha ,Expa1	C-76-DI-B	Val-C-DI-B	0.42	17.80	5.41

Araip.BP0EH	Glucan Endo1,3Beta Basic Isoform	C-76-DI-B	Val-C-DI-B	0.30	13.04	5.43
Araip.JWB3B	Allergen Pru	C-76-DI-B	Val-C-DI-B	6.39	283.21	5.47
Araip.8C4ZH	Kunitz Trypsin Protease Inhibitor	C-76-DI-B	Val-C-DI-B	19.22	913.66	5.57
Araip.5X8UP	Mlp 43	C-76-DI-B	Val-C-DI-B	1.72	82.85	5.59
Araip.CH8F6	Ribonucleosidediphosphate Reductase Large Subunit	C-76-DI-B	Val-C-DI-B	0.06	3.83	6.06
Araip.3M3I5	Efr3 B	C-76-DI-B	Val-C-DI-B	1.34	97.06	6.18
Araip.SE39K	Expansin A10	C-76-DI-B	Val-C-DI-B	0.30	22.00	6.19
Araip.G5SR2	Amp Deaminase	C-76-DI-B	Val-C-DI-B	0.08	5.77	6.26
Araip.U02GD	Serine Threonine Phosphatase 5 Isoform X2	C-76-DI-B	Val-C-DI-B	0.31	24.11	6.27
Araip.FGG0P	Dna Mismatch Repair Muts Family	C-76-DI-B	Val-C-DI-B	0.05	4.79	6.51
Araip.7BB5W	Thioredoxin H2	C-76-DI-B	Val-C-DI-B	0.70	66.86	6.57
Araip.D0HHT	Ap4 Complex Subunit Mu	C-76-DI-B	Val-C-DI-B	0.14	13.50	6.62
Araip.X0I1L	Mlp 43	C-76-DI-B	Val-C-DI-B	0.39	51.32	7.02
Araip.3H4YH	Plasma Membrane Isoform 1	C-76-DI-B	Val-C-DI-B	0.42	55.05	7.03
Araip.T5AGT	Cyclic Nucleotidegated Ion Channel 15	C-76-DI-B	Val-C-DI-B	0.09	37.90	8.67
Aradu.1ZE7E	Homeoboxleucine Zipper Athb6	Val-C-FI-A	Val-C-DI-A	59.43	0.10	-9.19
Aradu.AGI8J	Plastid Movement Impaired 2	Val-C-FI-A	Val-C-DI-A	24.20	0.16	-7.21
Aradu.57J9Q	Myb G	Val-C-FI-A	Val-C-DI-A	4.12	0.03	-6.93
Aradu.S9NPM	Ubiquitin Ligase	Val-C-FI-A	Val-C-DI-A	28.02	0.71	-5.30
Aradu.94UA3	Uncharacterized Protein Loc101504672	Val-C-FI-A	Val-C-DI-A	12.60	0.35	-5.16
Aradu.QYL3B	Rna Polymerase Sigma Factor Chloroplastic	Val-C-FI-A	Val-C-DI-A	7.07	0.23	-4.93
Aradu.42MBK	Myosinj Heavy Chain	Val-C-FI-A	Val-C-DI-A	15.21	0.53	-4.84
Aradu.07384	Serine Threonine Phosphatase 4 Regulatory Subunit 2	Val-C-FI-A	Val-C-DI-A	16.00	0.79	-4.33
Aradu.PI7XA	Doublestrand Break Repair Mre11	Val-C-FI-A	Val-C-DI-A	40.40	2.48	-4.03
Aradu.18J05	Calnexin Homolog	Val-C-FI-A	Val-C-DI-A	8.49	0.53	-4.00
Aradu.E6GI1	Ty1Copia Retrotransposon	Val-C-FI-A	Val-C-DI-A	4.90	0.32	-3.94
Aradu.X0HJW	Abscisic Acid 8 Hydroxylase 2	Val-C-FI-A	Val-C-DI-A	7.52	0.50	-3.92
Aradu.HP9JD	Serine Threonine Kinase Nek6	Val-C-FI-A	Val-C-DI-A	1.44	0.10	-3.92
Aradu.IAN0B	Calciumbinding Mitochondrial Carrier S 1A	Val-C-FI-A	Val-C-DI-A	2.14	0.14	-3.90
Aradu.E00AS	Patatin 6	Val-C-FI-A	Val-C-DI-A	19.71	1.44	-3.78
Aradu.80YRY	Tetratricopeptide Repeat 1	Val-C-FI-A	Val-C-DI-A	235.15	17.25	-3.77
Aradu.K3I53	Glucuronokinase 1	Val-C-FI-A	Val-C-DI-A	3.47	0.26	-3.74
Aradu.JEF8K	Senescenceassociated Family	Val-C-FI-A	Val-C-DI-A	11.62	0.88	-3.73

Aradu.L8Z8Y	Methyl Binding Domaincontaining 10	Val-C-FI-A	Val-C-DI-A	5.98	0.45	-3.72
Aradu.W71HY	Agamous Madsbox Agl8	Val-C-FI-A	Val-C-DI-A	3.81	0.33	-3.52
Aradu.I80VP	Magnesiumprotoporphyrin Ix Monomethyl Ester	Val-C-FI-A	Val-C-DI-A	4.30	0.38	-3.49
Aradu.KQ4EG	Rho Guanine Nucleotide Exchange Factor 8	Val-C-FI-A	Val-C-DI-A	4.88	0.45	-3.45
Aradu.8V9QR	Gtype Lectin Sreceptor Serine Threonine Kinase	Val-C-FI-A	Val-C-DI-A	191.11	17.49	-3.45
Aradu.S44IV	Pathogenesisrelated Bet V I Family	Val-C-FI-A	Val-C-DI-A	5.23	0.49	-3.42
Aradu.MT7GR	Soft Fertilization Envelope	Val-C-FI-A	Val-C-DI-A	3.77	0.36	-3.40
Aradu.79NM3	Translocase Subunit Chloroplastic	Val-C-FI-A	Val-C-DI-A	21.28	2.09	-3.35
Aradu.G07EM	Plant F3C22140	Val-C-FI-A	Val-C-DI-A	2.59	0.26	-3.30
Aradu.ZI0DW	Chaperone Dnaj Chloroplastic	Val-C-FI-A	Val-C-DI-A	7.18	0.74	-3.27
Aradu.618YE	Sucrase Ferredoxin Family	Val-C-FI-A	Val-C-DI-A	6.00	0.65	-3.20
Aradu.PD37S	Transcription Factor Bhlh123	Val-C-FI-A	Val-C-DI-A	2.59	0.29	-3.18
Aradu.I1TYP	Rna Exonuclease 3	Val-C-FI-A	Val-C-DI-A	6.62	0.73	-3.18
Aradu.GA7X1	DNA Replication ATPdependent Helicase Nuclease	Val-C-FI-A	Val-C-DI-A	568.14	67.16	-3.08
Aradu.85KYS	Atpdependent Helicase Brm	Val-C-FI-A	Val-C-DI-A	80.37	9.63	-3.06
Aradu.M2PEK	Wat1Related At1G70260	Val-C-FI-A	Val-C-DI-A	187.16	22.42	-3.06
Aradu.46UHL	Calciumbinding Mitochondrial Carrier S 2 Isoform X	Val-C-FI-A	Val-C-DI-A	104.79	12.73	-3.04
Aradu.ABT8D	Eukaryotic Translation Initiation Factor 5	Val-C-FI-A	Val-C-DI-A	4.74	0.59	-3.02
Aradu.JIB7P	Poly(Adpribose) Glycohydrolase 1 Isoform X1	Val-C-FI-A	Val-C-DI-A	7.40	0.92	-3.01
Aradu.8HL1Z	Cullinassociated Nedd8Dissociated 1	Val-C-FI-A	Val-C-DI-A	41.18	5.14	-3.00
Aradu.XQZ5U	Exordium 5	Val-C-FI-A	Val-C-DI-A	0.69	5.50	3.00
Aradu.637TZ	Asparagine Synthetase	Val-C-FI-A	Val-C-DI-A	28.91	231.64	3.00
Aradu.68MP6	Blue Copper	Val-C-FI-A	Val-C-DI-A	1.36	10.90	3.01
Aradu.7Y3DJ	Glucan Endo1,3Betaglucosidase 3	Val-C-FI-A	Val-C-DI-A	3.37	27.27	3.02
Aradu.XF675	Atpase Alpha Partial	Val-C-FI-A	Val-C-DI-A	5.68	46.23	3.02
Aradu.GEE52	Mannose1Phosphate Guanyltransferase Alphab	Val-C-FI-A	Val-C-DI-A	0.33	2.67	3.03
Aradu.U8TGW	Lysm Domain Receptor Kinase 4	Val-C-FI-A	Val-C-DI-A	0.28	2.31	3.03
Aradu.I2J7R	Lob Domaincontaining 4	Val-C-FI-A	Val-C-DI-A	6.80	55.76	3.03
Aradu.025HX	Ribosomal S12 (Chloroplast)	Val-C-FI-A	Val-C-DI-A	0.66	5.48	3.04
Aradu.PXP20	Receptor Kinase Hsl1	Val-C-FI-A	Val-C-DI-A	0.05	0.43	3.06
Aradu.RU8HS	Late Embryogenesis Abundant	Val-C-FI-A	Val-C-DI-A	0.44	3.64	3.06
Aradu.L46QG	Glucan Endo1,3Betaglucosidase 5	Val-C-FI-A	Val-C-DI-A	0.93	7.75	3.06
Aradu.I36IV	Stemspecific Tsjt1	Val-C-FI-A	Val-C-DI-A	0.44	3.66	3.07

Aradu.KRX9K	Iaaamino Acid Hydrolase Ilr1 4	Val-C-FI-A	Val-C-DI-A	0.22	1.81	3.07
Aradu.XC1GR	Gdsl Esterase Lipase	Val-C-FI-A	Val-C-DI-A	0.12	0.99	3.08
Aradu.Y328N	Chitinase 2	Val-C-FI-A	Val-C-DI-A	0.31	2.63	3.08
Aradu.V1798	Serine Threonine Phosphatase 7 Long Form Homolog	Val-C-FI-A	Val-C-DI-A	0.09	0.76	3.08
Aradu.25DKA	Nonspecific Lipidtransfer 4	Val-C-FI-A	Val-C-DI-A	12.49	106.16	3.09
Aradu.BX6BE	Malonylco-A:Anthocyanin3Ogluc6Omalonyltransfer	Val-C-FI-A	Val-C-DI-A	1.28	10.92	3.09
Aradu.2W676	Cationic Peroxidase 1	Val-C-FI-A	Val-C-DI-A	0.61	5.23	3.09
Aradu.WX3Q6	Atp Synthase Cf1 Alpha Subunit (Chloroplast)	Val-C-FI-A	Val-C-DI-A	0.44	3.78	3.09
Aradu.I3YXT	Hothead	Val-C-FI-A	Val-C-DI-A	0.39	3.36	3.10
Aradu.F0D6Z	Zinc Transporter 8	Val-C-FI-A	Val-C-DI-A	16.19	139.55	3.11
Aradu.X06N9	Not Transcription Complex Subunit Vip2	Val-C-FI-A	Val-C-DI-A	0.56	4.92	3.13
Aradu.Z49Y7	Plant T7H2070	Val-C-FI-A	Val-C-DI-A	1.23	10.75	3.13
Aradu.G88H9	Dnadirected Rna Polymerase Third Largest Subunit	Val-C-FI-A	Val-C-DI-A	2.06	18.08	3.13
Aradu.YD5BQ	Indole3Acetate Omethyltransferase 1	Val-C-FI-A	Val-C-DI-A	0.14	1.23	3.14
Aradu.AF71L	Atp Synthase Cf1 Alpha Subunit (Chloroplast)	Val-C-FI-A	Val-C-DI-A	1.52	13.51	3.15
Aradu.K0Y9B	Gmp Synthase [Glutaminehydrolyzing]	Val-C-FI-A	Val-C-DI-A	0.26	2.29	3.16
Aradu.D8UNY	Peroxidase 47	Val-C-FI-A	Val-C-DI-A	0.27	2.45	3.19
Aradu.YA6WS	Expansina15 Precursor	Val-C-FI-A	Val-C-DI-A	1.72	15.72	3.19
Aradu.S13AZ	Adenine Nucleotide Alpha Hydrolases Superfamily	Val-C-FI-A	Val-C-DI-A	0.34	3.10	3.20
Aradu.S88B1	Cell Wallassociated Partial	Val-C-FI-A	Val-C-DI-A	50.88	474.98	3.22
Aradu.4VP01	Nadhplastoquinone Oxidoreductase Subunit 1	Val-C-FI-A	Val-C-DI-A	3.45	33.15	3.26
Aradu.K88X4	Photosytem I Subunit Vii	Val-C-FI-A	Val-C-DI-A	1.11	10.73	3.27
Aradu.TMI5W	Growthregulating Factor 6	Val-C-FI-A	Val-C-DI-A	0.13	1.24	3.29
Aradu.LCM7P	Casp Aralydraft_485429	Val-C-FI-A	Val-C-DI-A	1.30	12.77	3.29
Aradu.T2SCC	Serine Threonine Kinase	Val-C-FI-A	Val-C-DI-A	2.51	24.74	3.30
Aradu.X0FA9	Senescenceassociated Family	Val-C-FI-A	Val-C-DI-A	3.87	38.99	3.33
Aradu.G3DHZ	Pectate Lyase	Val-C-FI-A	Val-C-DI-A	1.14	11.53	3.33
Aradu.PG6LA	Glucomannan 4Betamannosyltransferase 2	Val-C-FI-A	Val-C-DI-A	5.20	52.91	3.35
Aradu.32RQ0	Ringh2 Finger Atl3	Val-C-FI-A	Val-C-DI-A	0.47	4.75	3.35
Aradu.UP79J	Root Meristem Growth Factor 9	Val-C-FI-A	Val-C-DI-A	2.11	21.59	3.36
Aradu.QVN0R	Udparabinopyranose Mutase 1	Val-C-FI-A	Val-C-DI-A	2.72	28.26	3.38
Aradu.1X84T	Exordium 2	Val-C-FI-A	Val-C-DI-A	1.54	15.94	3.38
Aradu.ZKQ4D	Atp Synthase Cf0 Subunit Iv (Chloroplast)	Val-C-FI-A	Val-C-DI-A	0.13	1.41	3.39

Aradu.WB4HU	Lactosylceramide 4Alphagalactosyltransferase	Val-C-FI-A	Val-C-DI-A	0.28	2.99	3.40
Aradu.88B96	Beta1,4Xylosyltransferase Irx9	Val-C-FI-A	Val-C-DI-A	0.27	2.91	3.41
Aradu.LIS00	Rac Gtpbinding Rac2	Val-C-FI-A	Val-C-DI-A	0.63	6.71	3.41
Aradu.6JB4Q	Pollenspecific Sf21	Val-C-FI-A	Val-C-DI-A	0.29	3.09	3.43
Aradu.AYN79	Nad(P)Binding Rossmannfold	Val-C-FI-A	Val-C-DI-A	3.63	39.56	3.44
Aradu.413LK	Photosystem Ii D2 (Chloroplast)	Val-C-FI-A	Val-C-DI-A	0.10	1.09	3.46
Aradu.TEW52	Btb Poz Domaincontaining At3G49900	Val-C-FI-A	Val-C-DI-A	0.57	6.47	3.50
Aradu.ZB3Q7	Tbc1 Domain Family Member 15	Val-C-FI-A	Val-C-DI-A	1.93	21.86	3.50
Aradu.KHH68	Aquaporin Tip13	Val-C-FI-A	Val-C-DI-A	17.18	195.38	3.51
Aradu.K2F98	Alpha Tubulin 1	Val-C-FI-A	Val-C-DI-A	0.46	5.31	3.52
Aradu.39MPT	Plantspecific Domain Tigr01589 Family	Val-C-FI-A	Val-C-DI-A	14.35	165.19	3.52
Aradu.NN4ME	2Aminoethanethiol Dioxygenase	Val-C-FI-A	Val-C-DI-A	0.07	0.85	3.54
Aradu.0R425	Uncharacterized Membrane At1G06890	Val-C-FI-A	Val-C-DI-A	0.59	7.05	3.58
Aradu.8L0QQ	Bifunctional Polynucleotide Phosphatase Kinase	Val-C-FI-A	Val-C-DI-A	0.59	7.09	3.58
Aradu.R7QS7	Nadhplastoquinone Oxidoreductoase (Chloroplast)	Val-C-FI-A	Val-C-DI-A	0.29	3.54	3.60
Aradu.D5ZBD	Fringerelated Family	Val-C-FI-A	Val-C-DI-A	0.21	2.54	3.61
Aradu.3S3UE	Leucinerich Repeat Family	Val-C-FI-A	Val-C-DI-A	0.17	2.19	3.65
Aradu.NHB6E	Trichome Birefringence 33	Val-C-FI-A	Val-C-DI-A	0.19	2.43	3.66
Aradu.P709D	Gdsl Esterase Lipase At2G04570	Val-C-FI-A	Val-C-DI-A	0.16	2.05	3.67
Aradu.M0QVM	Probable Galacturonosyltransferase 1	Val-C-FI-A	Val-C-DI-A	0.20	2.61	3.67
Aradu.JZT16	Expansin 2	Val-C-FI-A	Val-C-DI-A	2.13	27.33	3.68
Aradu.G09L8	Notum Homolog	Val-C-FI-A	Val-C-DI-A	0.50	6.44	3.69
Aradu.4V2X0	Thioredoxin H2	Val-C-FI-A	Val-C-DI-A	5.27	69.54	3.72
Aradu.420FT	Uncharacterized Protein Loc100790018	Val-C-FI-A	Val-C-DI-A	0.80	10.73	3.75
Aradu.EQ6UY	Fbox Pp2B15	Val-C-FI-A	Val-C-DI-A	1.74	23.80	3.77
Aradu.GS29Q	Alphaamylase Subtilisin Inhibitor	Val-C-FI-A	Val-C-DI-A	6.70	91.96	3.78
Aradu.U9FPR	Ribosomal S15 (Chloroplast)	Val-C-FI-A	Val-C-DI-A	0.79	10.99	3.79
Aradu.13SFN	Gdsl Esterase Lipase	Val-C-FI-A	Val-C-DI-A	0.17	2.44	3.81
Aradu.0GP4E	Thaumatin	Val-C-FI-A	Val-C-DI-A	0.23	3.29	3.82
Aradu.G235T	Expansin A10	Val-C-FI-A	Val-C-DI-A	1.73	24.92	3.85
Aradu.N9RDH	Atp Synthase Cf0 Subunit Iv (Chloroplast)	Val-C-FI-A	Val-C-DI-A	0.27	3.93	3.85
Aradu.GXJ7L	Pollen Ole E 1 Allergen And Extensin Family	Val-C-FI-A	Val-C-DI-A	0.37	5.42	3.86
Aradu.28P5F	Bon1Associated 2	Val-C-FI-A	Val-C-DI-A	0.32	4.63	3.87

Aradu.BX0IC	Bon1Associated 2	Val-C-FI-A	Val-C-DI-A	2.18	32.30	3.89
Aradu.AW9GY	14 Kda Prolinerich	Val-C-FI-A	Val-C-DI-A	9.88	148.79	3.91
Aradu.AI0TY	Hypothetical Chloroplast Rf1 (Chloroplast)	Val-C-FI-A	Val-C-DI-A	0.45	6.85	3.92
Aradu.P6ZPS	Xyloglucan Endotransglucosylase Hydrolase 32	Val-C-FI-A	Val-C-DI-A	11.99	182.49	3.93
Aradu.A834G	Fasciclin Arabinogalactan 12	Val-C-FI-A	Val-C-DI-A	0.37	5.66	3.95
Aradu.P2130	Monothiol Glutaredoxins6	Val-C-FI-A	Val-C-DI-A	0.90	14.39	4.00
Aradu.B1FJV	Blue Copper	Val-C-FI-A	Val-C-DI-A	0.21	3.47	4.02
Aradu.M3XI9	Gdsl Lipase Acylhydrolase	Val-C-FI-A	Val-C-DI-A	0.11	1.73	4.03
Aradu.1T3UD	Kda Prolinerich	Val-C-FI-A	Val-C-DI-A	2.33	38.67	4.05
Aradu.WYR9Z	Atp Synthase Cf1 Alpha Subunit (Chloroplast)	Val-C-FI-A	Val-C-DI-A	4.76	79.57	4.06
Aradu.9AK3C	Asparticase	Val-C-FI-A	Val-C-DI-A	0.29	4.93	4.10
Aradu.P0ZYD	Glucose6Phosphate Phosphate Translocator	Val-C-FI-A	Val-C-DI-A	10.50	180.73	4.11
Aradu.YW2J0	Patatin 7	Val-C-FI-A	Val-C-DI-A	0.19	3.27	4.12
Aradu.AW9GY	14 Kda Prolinerich	Val-C-FI-A	Val-C-DI-A	4.87	85.73	4.14
Aradu.BWM82	Serine Decarboxylase	Val-C-FI-A	Val-C-DI-A	0.10	1.79	4.14
Aradu.H4BH8	Peroxisome Biogenesis 7	Val-C-FI-A	Val-C-DI-A	0.65	11.71	4.16
Aradu.5Z359	Patatin 2	Val-C-FI-A	Val-C-DI-A	1.71	30.89	4.18
Aradu.6U69W	Nadhplastoquinone Oxidoreductase Subunit K	Val-C-FI-A	Val-C-DI-A	0.30	5.66	4.23
Aradu.3GX6J	Pollen Ole E I	Val-C-FI-A	Val-C-DI-A	0.74	14.21	4.26
Aradu.79I5D	Germin Subfamily 2 Member 4	Val-C-FI-A	Val-C-DI-A	0.18	3.42	4.27
Aradu.78K64	Plastocyanin Domain	Val-C-FI-A	Val-C-DI-A	0.48	9.31	4.28
Aradu.CYG6N	Gonadotropin Beta Chain	Val-C-FI-A	Val-C-DI-A	3.15	61.30	4.28
Aradu.UHQ7N	Uncharacterized Protein Loc101507125 Isoform X2	Val-C-FI-A	Val-C-DI-A	0.34	6.71	4.30
Aradu.TZ1M5	Phosphatidylglycerol Phosphatidylinositol TransferD	Val-C-FI-A	Val-C-DI-A	0.42	8.49	4.34
Aradu.1P1D6	Zip Zinc Iron Transport Family	Val-C-FI-A	Val-C-DI-A	8.14	166.36	4.35
Aradu.1FT6I	Ribosomal S3 (Chloroplast)	Val-C-FI-A	Val-C-DI-A	1.40	28.64	4.36
Aradu.M7NEJ	Bifunctional 3Dehydroquinate Dehydratase Shikimat	Val-C-FI-A	Val-C-DI-A	0.86	17.59	4.36
Aradu.130HL	U6 Snrnaassociated Sm Lsm6	Val-C-FI-A	Val-C-DI-A	0.38	8.09	4.41
Aradu.HK886	Diphthamide Biosynthesis 1	Val-C-FI-A	Val-C-DI-A	0.02	0.47	4.43
Aradu.M1BLH	Monothiol Glutaredoxins6	Val-C-FI-A	Val-C-DI-A	3.06	66.14	4.44
Aradu.XGK7G	Wallassociated Receptor Kinase	Val-C-FI-A	Val-C-DI-A	0.15	3.30	4.45
Aradu.U86RL	12Oxophytodienoate Reductase 11	Val-C-FI-A	Val-C-DI-A	0.15	3.40	4.47
Aradu.9FN01	Premrnasplicing Factor Sf2	Val-C-FI-A	Val-C-DI-A	0.70	15.97	4.51

Aradu.49PAS	Isoflavone 2 Hydroxylase	Val-C-FI-A	Val-C-DI-A	0.94	23.61	4.64
Aradu.UN18Y	Peroxisomal Voltagedependent AnionselectiveChann	Val-C-FI-A	Val-C-DI-A	0.25	6.38	4.70
Aradu.CZ6KH	Hxxxdtype Acyltransferase Family	Val-C-FI-A	Val-C-DI-A	0.29	7.54	4.72
Aradu.MJ134	21 KDa	Val-C-FI-A	Val-C-DI-A	2.02	58.65	4.86
Aradu.S1EHV	DnA Mismatch Repair Msh6	Val-C-FI-A	Val-C-DI-A	0.84	24.45	4.87
Aradu.1W5FD	BON1Associated 2	Val-C-FI-A	Val-C-DI-A	0.33	9.61	4.88
Aradu.K3ZSF	Expansina8 Precursor	Val-C-FI-A	Val-C-DI-A	0.76	23.64	4.97
Aradu.WLE0A	Expansin A10	Val-C-FI-A	Val-C-DI-A	0.71	24.01	5.08
Aradu.39755	Kdeltailed Cysteine Endopeptidase Cep1	Val-C-FI-A	Val-C-DI-A	1.08	36.50	5.08
Aradu.AA5UH	Xyloglucan Endotransglucosylase Hydrolase	Val-C-FI-A	Val-C-DI-A	0.16	5.73	5.18
Aradu.WSW8I	Plant F18G18200	Val-C-FI-A	Val-C-DI-A	0.67	32.51	5.59
Araip.DZ2WD	Actindepolymerizing Factor 2	Val-C-FI-B	Val-C-DI-B	19.91	0.50	-5.32
Araip.NY165	K+H+ Exchange	Val-C-FI-B	Val-C-DI-B	3.89	0.21	-4.23
Araip.DDF1I	Transcription Factor Asg4	Val-C-FI-B	Val-C-DI-B	8.96	0.57	-3.97
Araip.E7LPR	Oxygenevolving Enhancer 2	Val-C-FI-B	Val-C-DI-B	57.66	3.81	-3.92
Araip.IBB26	Terpene Synthase Metalbinding Domain	Val-C-FI-B	Val-C-DI-B	6.96	0.47	-3.88
Araip.MXY7L	Trichome Birefringence 10	Val-C-FI-B	Val-C-DI-B	2.65	0.19	-3.83
Araip.76C4P	Gpnloop GTPase 1 Homolog Isoform X1	Val-C-FI-B	Val-C-DI-B	2.89	0.21	-3.80
Araip.XV9A7	Glutathione Stransferase T3	Val-C-FI-B	Val-C-DI-B	0.54	0.04	-3.77
Araip.RL6MC	(+)Neomenthol Dehydrogenase	Val-C-FI-B	Val-C-DI-B	4.90	0.36	-3.77
Araip.HGW1G	Probable Lrr Receptor Serine Threonine Kinase	Val-C-FI-B	Val-C-DI-B	5.58	0.41	-3.75
Araip.I73VG	Glucuronokinase 1	Val-C-FI-B	Val-C-DI-B	7.24	0.56	-3.70
Araip.DMI2F	Integral Membrane Family	Val-C-FI-B	Val-C-DI-B	11.32	0.89	-3.67
Araip.G4DKZ	Calnexin Homolog	Val-C-FI-B	Val-C-DI-B	22.44	1.77	-3.66
Araip.0Z62R	1Aminocyclopropane1Carboxylate Oxidase Homolog	Val-C-FI-B	Val-C-DI-B	19.29	1.53	-3.65
Araip.2LP4Y	Gibberellin 20 Oxidase 1	Val-C-FI-B	Val-C-DI-B	1.68	0.13	-3.65
Araip.V23ZE	Flowering Locus T	Val-C-FI-B	Val-C-DI-B	199.23	15.96	-3.64
Araip.3DN9J	Dna Rnabinding Kin17	Val-C-FI-B	Val-C-DI-B	11.33	0.94	-3.60
Araip.8P2IP	Atpcitrate Synthase Beta Chain 1	Val-C-FI-B	Val-C-DI-B	6.18	0.54	-3.51
Araip.9K47M	Probable Phosphatase 2C 76	Val-C-FI-B	Val-C-DI-B	4.27	0.38	-3.50
Araip.68AAR	Acyl Binding Domaincontaining 4	Val-C-FI-B	Val-C-DI-B	2.59	0.24	-3.43
Araip.VKU4A	Thiol Protease Aleurain	Val-C-FI-B	Val-C-DI-B	4.72	0.45	-3.39
Araip.68YBY	Rj2	Val-C-FI-B	Val-C-DI-B	2.55	0.24	-3.38

Araip.T1SIZ	Flowering Locus T	Val-C-FI-B	Val-C-DI-B	38.51	3.72	-3.37
Araip.DM9S9	Receptor 12 Isoform X1	Val-C-FI-B	Val-C-DI-B	1.72	0.17	-3.33
Araip.L20X2	Uncharacterized Protein	Val-C-FI-B	Val-C-DI-B	5.65	0.60	-3.24
Araip.KY4DP	Histidine Kinase 1	Val-C-FI-B	Val-C-DI-B	0.96	0.10	-3.22
Araip.6S6W2	Auxininduced 5Ng4	Val-C-FI-B	Val-C-DI-B	4.55	0.49	-3.21
Araip.51VQE	Sucrose Synthase 6	Val-C-FI-B	Val-C-DI-B	2.74	0.30	-3.20
Araip.L5JPH	Glutamine Dumper 5	Val-C-FI-B	Val-C-DI-B	5.88	0.65	-3.18
Araip.QC465	Salicylate Omethyltransferase	Val-C-FI-B	Val-C-DI-B	7.14	0.79	-3.18
Araip.TXS4L	Uncharacterized Wd Repeatcontaining Isoform X1	Val-C-FI-B	Val-C-DI-B	12.26	1.38	-3.15
Araip.TJ4P5	Nrt1 Ptr Family	Val-C-FI-B	Val-C-DI-B	0.96	0.11	-3.14
Araip.AR3S4	Plastid Movement Impaired	Val-C-FI-B	Val-C-DI-B	86.02	9.97	-3.11
Araip.S175R	Isoleucine Nmonooxygenase 2	Val-C-FI-B	Val-C-DI-B	8.41	0.98	-3.10
Araip.97T9K	Uncharacterized Protein Loc101255289	Val-C-FI-B	Val-C-DI-B	3.78	0.45	-3.07
Araip.FJW22	Probable Gpianchored Adhesin Pga55	Val-C-FI-B	Val-C-DI-B	26.66	3.23	-3.05
Araip.9X632	Hydroxyprolinerich Glyco	Val-C-FI-B	Val-C-DI-B	3.61	0.45	-3.01
Araip.0U8TC	Transportin3 Isoform 1	Val-C-FI-B	Val-C-DI-B	5.91	0.74	-3.00
Araip.0G8MF	Trichome Birefringence 42	Val-C-FI-B	Val-C-DI-B	0.24	1.89	3.00
Araip.QK072	Root Phototropism 3	Val-C-FI-B	Val-C-DI-B	1.24	9.90	3.00
Araip.B43HB	Atp Synthase Cf0 Subunit Iv (Chloroplast)	Val-C-FI-B	Val-C-DI-B	1.04	8.39	3.01
Araip.5M5DL	Ralf 34	Val-C-FI-B	Val-C-DI-B	0.64	5.22	3.02
Araip.47DVE	Prolinerich 4	Val-C-FI-B	Val-C-DI-B	3.01	24.68	3.03
Araip.13WAT	Hxxxdtype Acyltransferase Family	Val-C-FI-B	Val-C-DI-B	1.62	13.32	3.04
Araip.X3MVE	Atp Synthase Cf0 Subunit Iv (Chloroplast)	Val-C-FI-B	Val-C-DI-B	0.35	2.92	3.04
Araip.YB61P	Uncharacterized PrLoc101506875	Val-C-FI-B	Val-C-DI-B	1.81	14.96	3.04
Araip.BB9A1	Serine Threonine Planttype	Val-C-FI-B	Val-C-DI-B	0.24	2.02	3.05
Araip.7HD9S	Chitinase 2	Val-C-FI-B	Val-C-DI-B	0.29	2.45	3.06
Araip.XM65N	Endoglucanase 24	Val-C-FI-B	Val-C-DI-B	0.52	4.31	3.06
Araip.7KT30	Pentatricopeptide Repeatcontaining Mitochondrial	Val-C-FI-B	Val-C-DI-B	0.94	7.85	3.06
Araip.H1W3S	Growthregulating Factor 6	Val-C-FI-B	Val-C-DI-B	0.15	1.22	3.07
Araip.E2PJR	Lrr Receptor Serine Threonine Kinase Gso1	Val-C-FI-B	Val-C-DI-B	0.34	2.87	3.07
Araip.61BVJ	Glucan Endo1,3Betaglucosidase 5	Val-C-FI-B	Val-C-DI-B	0.90	7.63	3.08
Araip.87H0B	Acid Phosphatase 1	Val-C-FI-B	Val-C-DI-B	1.06	8.98	3.08
Araip.X3K6D	Ribosomal L2 (Chloroplast)	Val-C-FI-B	Val-C-DI-B	1.22	10.43	3.10

Araip.U0G8M	Cell Wallassociated Partial	Val-C-FI-B	Val-C-DI-B	5.48	47.00	3.10
Araip.6L5KM	Calmodulin 3	Val-C-FI-B	Val-C-DI-B	0.85	7.34	3.11
Araip.038T5	Tmv Resistance N	Val-C-FI-B	Val-C-DI-B	0.21	1.86	3.12
Araip.1ZJ86	Lysm Domain Receptor Kinase 4	Val-C-FI-B	Val-C-DI-B	0.28	2.42	3.12
Araip.XH0KD	Malonylco-A:Anthocyanin3Ogluco6Omalonyltransfe	Val-C-FI-B	Val-C-DI-B	0.44	3.93	3.16
Araip.06PW6	Receptor Kinase Hsl1	Val-C-FI-B	Val-C-DI-B	0.05	0.43	3.21
Araip.E0Q5W	Glucan Endo1,3Betaglucosidase 4	Val-C-FI-B	Val-C-DI-B	0.06	0.51	3.21
Araip.CF2W6	Uric Acid Degradation Bifunctional Ttl Isoform X2	Val-C-FI-B	Val-C-DI-B	0.28	2.64	3.22
Araip.K461S	1Deoxydxylulose5Phosphate Synthase	Val-C-FI-B	Val-C-DI-B	0.26	2.47	3.23
Araip.WY33Y	Cationic Peroxidase 1	Val-C-FI-B	Val-C-DI-B	0.34	3.19	3.23
Araip.6P9JS	Glucomannan 4Betamannosyltransferase 2	Val-C-FI-B	Val-C-DI-B	6.54	61.63	3.24
Araip.WY8CJ	Indole3Acetate Omethyltransferase 1	Val-C-FI-B	Val-C-DI-B	0.14	1.30	3.25
Araip.RA8PB	Ethyleneresponsive Transcription Factor Erf023	Val-C-FI-B	Val-C-DI-B	3.05	29.15	3.26
Araip.CAX6Y	Zinc Transporter 5	Val-C-FI-B	Val-C-DI-B	13.13	126.51	3.27
Araip.C5RH9	Casp Aralydraft_485429	Val-C-FI-B	Val-C-DI-B	1.73	16.76	3.28
Araip.VW283	Expansina15 Precursor	Val-C-FI-B	Val-C-DI-B	1.83	17.79	3.28
Araip.BL74U	Atp Synthase Cf1 Alpha Partial	Val-C-FI-B	Val-C-DI-B	2.96	28.83	3.28
Araip.Y41TM	Beta1,4Xylosyltransferase Irx9	Val-C-FI-B	Val-C-DI-B	0.30	2.98	3.29
Araip.RT7B0	Cytochrome C Oxidase Assembly Cox19	Val-C-FI-B	Val-C-DI-B	0.83	8.08	3.29
Araip.HDP7H	Transferring Glycosyl Group Transferase	Val-C-FI-B	Val-C-DI-B	0.37	3.64	3.30
Araip.X8EK5	Plant T7H2070	Val-C-FI-B	Val-C-DI-B	1.23	12.15	3.30
Araip.GC9VN	Pectate Lyase	Val-C-FI-B	Val-C-DI-B	1.07	10.56	3.31
Araip.L9WSM	Exordium 2	Val-C-FI-B	Val-C-DI-B	1.55	15.33	3.31
Araip.W9BBB	Uncharacterized PrLoc100790018	Val-C-FI-B	Val-C-DI-B	0.64	6.34	3.31
Araip.V628M	Vacuolarprocessing Enzyme	Val-C-FI-B	Val-C-DI-B	1.35	13.68	3.34
Araip.5V59L	Cysteine Ase Inhibitor 1	Val-C-FI-B	Val-C-DI-B	348.38	3542.92	3.35
Araip.QD22A	Abc Transporter G Family Member 11	Val-C-FI-B	Val-C-DI-B	0.25	2.60	3.35
Araip.Y37JR	Acrs (Chloroplast)	Val-C-FI-B	Val-C-DI-B	0.80	8.22	3.36
Araip.R7MQK	Lactosylceramide 4Alphagalactosyltransferase	Val-C-FI-B	Val-C-DI-B	0.28	2.90	3.36
Araip.FHQ6L	Histone Deacetylase 9 Isoform X2	Val-C-FI-B	Val-C-DI-B	2.29	23.66	3.37
Araip.04C4D	Rna Polymerase Beta Subunit (Chloroplast)	Val-C-FI-B	Val-C-DI-B	0.14	1.43	3.37
Araip.GT9V5	Atp Synthase Cf1 Alpha Subunit (Chloroplast)	Val-C-FI-B	Val-C-DI-B	11.95	123.96	3.37
Araip.N335X	Senescenceassociated Family	Val-C-FI-B	Val-C-DI-B	3.72	39.02	3.39

Araip.B6Q3S	Gdsl Esterase Lipase	Val-C-FI-B	Val-C-DI-B	0.17	1.78	3.40
Araip.I8RB5	Hypothetical Chloroplast Rf19 (Chloroplast)	Val-C-FI-B	Val-C-DI-B	0.36	3.82	3.42
Araip.MS7L3	Nad(P)Binding Rossmannfold	Val-C-FI-B	Val-C-DI-B	3.63	38.86	3.42
Araip.KRG2J	Serine Threonine Phosphatase 7 Long Form Homolog	Val-C-FI-B	Val-C-DI-B	0.08	0.87	3.43
Araip.83LG1	Duf4228 Domain	Val-C-FI-B	Val-C-DI-B	4.21	45.78	3.44
Araip.ZS1T2	Fbox At1G61340	Val-C-FI-B	Val-C-DI-B	0.35	3.81	3.45
Araip.20CID	Chitinase (Class Ib) Hevein	Val-C-FI-B	Val-C-DI-B	0.65	7.36	3.49
Araip.S6YA0	Purple Acid Phosphatase 17	Val-C-FI-B	Val-C-DI-B	1.47	16.64	3.50
Araip.BG5YS	Btb Poz Domaincontaining At3G49900	Val-C-FI-B	Val-C-DI-B	0.56	6.40	3.51
Araip.94IT6	Cytochrome B Reductase 1	Val-C-FI-B	Val-C-DI-B	1.35	15.41	3.51
Araip.EMI4R	Alpha1,4Glucan Synthase [Udpforming] 2	Val-C-FI-B	Val-C-DI-B	2.60	29.91	3.53
Araip.321WW	Probable Galacturonosyltransferase 1	Val-C-FI-B	Val-C-DI-B	0.16	1.84	3.56
Araip.4RI8H	Pollen Ole E 1 Allergen And Extensin Family	Val-C-FI-B	Val-C-DI-B	0.63	7.48	3.57
Araip.35VAB	Uncharacterized Membrane At1G06890	Val-C-FI-B	Val-C-DI-B	0.59	7.02	3.57
Araip.CR2PP	Minichromosome Instability 12	Val-C-FI-B	Val-C-DI-B	0.36	4.32	3.57
Araip.U5J78	Aquaporin Tip21	Val-C-FI-B	Val-C-DI-B	26.07	311.58	3.58
Araip.Y9DP8	Dna Mismatch Repair Msh3 Isoform X1	Val-C-FI-B	Val-C-DI-B	1.35	16.16	3.58
Araip.RVY5J	Gdsl Esterase Lipase At2G04570	Val-C-FI-B	Val-C-DI-B	0.17	2.09	3.62
Araip.EA0AH	Thaumatin	Val-C-FI-B	Val-C-DI-B	0.17	2.08	3.62
Araip.IN97X	Fbox Pp2B15	Val-C-FI-B	Val-C-DI-B	0.23	2.81	3.63
Araip.XVW43	Fbox Pp2B15	Val-C-FI-B	Val-C-DI-B	1.53	18.94	3.63
Araip.JPJ83	Pathogenesisrelated Class 10	Val-C-FI-B	Val-C-DI-B	0.34	4.28	3.63
Araip.NLH67	Hypothetical Chloroplast Rf21 (Chloroplast)	Val-C-FI-B	Val-C-DI-B	0.18	2.19	3.63
Araip.GV2U7	Adenine Nucleotide Alpha Hydrolases Superfamily	Val-C-FI-B	Val-C-DI-B	0.24	3.07	3.66
Araip.97TWR	Notum Homolog	Val-C-FI-B	Val-C-DI-B	0.53	6.77	3.66
Araip.E30MW	Expansin 2	Val-C-FI-B	Val-C-DI-B	1.88	24.26	3.69
Araip.4BJ8N	Chitinase Homologue	Val-C-FI-B	Val-C-DI-B	0.13	1.62	3.69
Araip.UC7G4	Calmodulinbinding Isoform Partial	Val-C-FI-B	Val-C-DI-B	0.09	1.21	3.69
Araip.SK1X8	Trichome Birefringence 33	Val-C-FI-B	Val-C-DI-B	0.16	2.07	3.69
Araip.BV4C5	Permease Transmembrane	Val-C-FI-B	Val-C-DI-B	0.53	6.91	3.70
Araip.4M817	Plantspecific Domain Tigr01589 Family	Val-C-FI-B	Val-C-DI-B	36.58	476.48	3.70
Araip.5L3CH	50S Ribosomal L9	Val-C-FI-B	Val-C-DI-B	0.37	4.83	3.72
Araip.I0HKJ	Xyloglucan Endotransglucosylase Hydrolase 32	Val-C-FI-B	Val-C-DI-B	10.25	137.54	3.75

Araip.M81B9	14 Kda Prolinerich	Val-C-FI-B	Val-C-DI-B	27.14	372.57	3.78
Araip.UJR3L	Zinc Finger Zat11	Val-C-FI-B	Val-C-DI-B	1.39	19.27	3.79
Araip.3WS5S	Ribosomal L20 (Chloroplast)	Val-C-FI-B	Val-C-DI-B	2.10	29.24	3.80
Araip.I8Z18	Arogenate Dehydrogenase Chloroplastic	Val-C-FI-B	Val-C-DI-B	1.24	17.47	3.81
Araip.UWV5P	Histonelysine Nmethyltransferase Atxr3	Val-C-FI-B	Val-C-DI-B	0.46	6.51	3.81
Araip.T2JQ6	Maturase K (Chloroplast)	Val-C-FI-B	Val-C-DI-B	0.33	4.58	3.82
Araip.9N5S4	Nac Domaincontaining 7	Val-C-FI-B	Val-C-DI-B	0.05	0.78	3.83
Araip.V7ZDG	Nadhplastoquinone Oxidoreductase Subunit 2	Val-C-FI-B	Val-C-DI-B	1.02	14.92	3.87
Araip.62GEG	Bon1Associated 2	Val-C-FI-B	Val-C-DI-B	2.30	33.61	3.87
Araip.I5AXB	Acetyltransferase (Gnat) Domain	Val-C-FI-B	Val-C-DI-B	0.42	6.28	3.90
Araip.QZA57	Expansin A10	Val-C-FI-B	Val-C-DI-B	1.24	18.55	3.90
Araip.31ZB6	Alphaamylase Subtilisin Inhibitor	Val-C-FI-B	Val-C-DI-B	6.89	104.21	3.92
Araip.W6I1W	Photosystem I P700 Apo Partial (Chloroplast)	Val-C-FI-B	Val-C-DI-B	0.60	9.23	3.95
Araip.7BB5W	Thioredoxin H2	Val-C-FI-B	Val-C-DI-B	4.24	66.86	3.98
Araip.CX2J8	B3 Domaincontaining	Val-C-FI-B	Val-C-DI-B	0.05	0.85	3.99
Araip.X36UP	Monothiol Glutaredoxins6	Val-C-FI-B	Val-C-DI-B	0.91	14.50	4.00
Araip.NR3WR	General Transcription Factor Iie Subunit 1	Val-C-FI-B	Val-C-DI-B	0.06	0.97	4.03
Araip.DV8IK	Blue Copper	Val-C-FI-B	Val-C-DI-B	0.22	3.56	4.04
Araip.ZB5LG	Pollen Ole E I	Val-C-FI-B	Val-C-DI-B	0.59	10.12	4.10
Araip.E274D	Nadhplastoquinone Oxidoreductase Subunit K	Val-C-FI-B	Val-C-DI-B	0.74	12.70	4.10
Araip.GQ5UU	Nadhplastoquinone Oxidoreductase Subunit Partial	Val-C-FI-B	Val-C-DI-B	0.65	11.15	4.10
Araip.K1GCE	Swi Snf Complex Subunit Swi3B	Val-C-FI-B	Val-C-DI-B	0.94	16.29	4.11
Araip.R1G22	Fringerelated Family	Val-C-FI-B	Val-C-DI-B	0.16	2.78	4.13
Araip.TK3NZ	Bifunctional 3Dehydroquinate Dehydratase Shikimat	Val-C-FI-B	Val-C-DI-B	1.12	19.59	4.13
Araip.U23Q6	Patatin 7	Val-C-FI-B	Val-C-DI-B	0.17	3.10	4.17
Araip.U8DJR	Atp Synthase Cf0 Subunit I (Chloroplast)	Val-C-FI-B	Val-C-DI-B	2.98	54.71	4.20
Araip.Y4HNU	Partial (Chloroplast)	Val-C-FI-B	Val-C-DI-B	0.25	4.68	4.24
Araip.JRH45	Probable Betadxylosidase 7	Val-C-FI-B	Val-C-DI-B	0.17	3.27	4.27
Araip.WX8L5	Isoflavone 2 Hydroxylase	Val-C-FI-B	Val-C-DI-B	1.04	20.48	4.29
Araip.V9JLA	Zip Zinc Iron Transport Family	Val-C-FI-B	Val-C-DI-B	15.18	298.07	4.30
Araip.YA4KW	Phosphatidylglycerol Phosphatidylinositol Transfer D	Val-C-FI-B	Val-C-DI-B	0.52	10.43	4.32
Araip.G67V4	Fasciclin Arabinogalactan	Val-C-FI-B	Val-C-DI-B	0.24	4.84	4.33
Araip.J6PP8	Germin Subfamily 2 Member 4	Val-C-FI-B	Val-C-DI-B	0.18	3.90	4.45

Araip.767YL	Gdsl Esterase Lipase Exl1	Val-C-FI-B	Val-C-DI-B	0.05	1.19	4.47
Araip.77QS1	Wallassociated Receptor Kinase	Val-C-FI-B	Val-C-DI-B	0.19	4.17	4.48
Araip.HEJ11	Monothiol Glutaredoxins6	Val-C-FI-B	Val-C-DI-B	3.75	86.76	4.53
Araip.TUE3L	E3 Ubiquitin Ligase Ring1	Val-C-FI-B	Val-C-DI-B	0.16	3.81	4.54
Araip.T1I8I	Uncharacterized PrLoc101507125 Isoform X2	Val-C-FI-B	Val-C-DI-B	0.32	7.58	4.56
Araip.NFP9Z	Kda Prolinerich	Val-C-FI-B	Val-C-DI-B	2.09	49.62	4.57
Araip.D3HLX	Kdeltailed Cysteine Endopeptidase Cep1	Val-C-FI-B	Val-C-DI-B	1.03	24.99	4.60
Araip.9A07Z	Phosphoinositide Phospholipase C 6	Val-C-FI-B	Val-C-DI-B	0.19	4.58	4.63
Araip.B4LS2	Gibberellinregulated 4	Val-C-FI-B	Val-C-DI-B	0.26	6.60	4.65
Araip.YH80N	Receptor Kinase	Val-C-FI-B	Val-C-DI-B	0.12	3.22	4.71
Araip.G3WHQ	Pra1 Family	Val-C-FI-B	Val-C-DI-B	0.44	11.66	4.74
Araip.Q2JPR	Bon1Associated 2	Val-C-FI-B	Val-C-DI-B	0.28	7.45	4.74
Araip.F5BPJ	Hypothetical Protein Glysoja_041633	Val-C-FI-B	Val-C-DI-B	0.15	4.48	4.89
Araip.NP9TU	Aspartic Ase Oryzasin1	Val-C-FI-B	Val-C-DI-B	0.11	3.30	4.92
Araip.RXA31	Expansina8 Precursor	Val-C-FI-B	Val-C-DI-B	0.79	25.02	4.98
Araip.7EX46	14 Kda Prolinerich	Val-C-FI-B	Val-C-DI-B	2.85	94.37	5.05
Araip.A0CQ3	Lectin Precursor	Val-C-FI-B	Val-C-DI-B	0.97	34.94	5.17
Araip.2H071	Xyloglucan Endotransglucosylase Hydrolase	Val-C-FI-B	Val-C-DI-B	0.14	5.09	5.18
Araip.77Y8N	Ureide Permease 2 Isoform X1	Val-C-FI-B	Val-C-DI-B	0.21	9.19	5.46
Araip.SE39K	Expansin A10	Val-C-FI-B	Val-C-DI-B	0.50	22.00	5.47
Araip.441FD	Homogentisate Phytyltransferase Chloroplastic	Val-C-FI-B	Val-C-DI-B	0.15	6.95	5.54
Araip.9WG11	NAD(P)Hdependent 6 Deoxychalcone Synthase	Val-C-FI-B	Val-C-DI-B	0.12	10.97	6.47
Araip.3M3I5	EFR3 B	Val-C-FI-B	Val-C-DI-B	0.98	97.06	6.63

Table S7: List of differentially expressed genes between C-76 and Val-C during full irrigation (FI) conditions

Gene id	Gene locus	C-76-FI (FPKM)	Val-C-FI (FPKM)	log2 fold	Annotation
Aradu.U86RL	Aradu.A05:18488502-18490271	0.99	0.15	-2.69	12-Oxophytodienoate Reductase 11
Aradu.5H0AD	Aradu.A07:71194093-71468341	22.87	0.12	-7.60	LRRReceptor-Like Serine Threonine- Kinase Fls2-Like
Aradu.TDC45	Aradu.A03:26559808-26561766	7.56	0.11	-6.10	Clavaminate Synthase At3G21360
Aradu.1H6YX	Aradu.A03:39874621-39890407	6.33	0.09	-6.09	Sieve Element Occlusion C
Aradu.1FT6I	Aradu.A07:13535513-13536380	81.76	1.40	-5.87	Ribosomal S3 (Chloroplast)
Aradu.0XN7S	Aradu.A04:121009626-121013152	3.95	0.07	-5.80	Epidermal Growth Factor Receptor Substrate 15-Like 1
Aradu.CMH7M	Aradu.A08:30074469-30078143	26.88	0.50	-5.74	Oxidoreductase Transition Metal Ion-Binding
Aradu.49PAS	Aradu.A04:20800676-20804904	38.90	0.94	-5.36	Isoflavone 2 -Hydroxylase-Like
Aradu.130HL	Aradu.A09:11310473-11314951	15.40	0.38	-5.34	U6 Snrna-Associated Sm Lsm6
Aradu.78K64	Aradu.A09:8043188-8046037	17.96	0.48	-5.22	Plastocyanin-Like Domain
Aradu.4X46H	Aradu.A07:17005899-17008602	2.47	0.07	-5.14	Poly(Rc)-Binding 1-Like
Aradu.WYR9Z	Adur1943:1839-2484	166.81	4.76	-5.13	ATP Synthase Cf1 Alpha Subunit (Chloroplast)
Aradu.AI0TY	Aradu.A06:44837293-44837947	15.88	0.45	-5.13	Hypothetical Chloroplast Rf1 (Chloroplast)
Aradu.9FN01	Aradu.A10:84023649-84025476	22.08	0.70	-4.98	Pre-Mrna-Splicing Factor Sf2
Aradu.U9FPR	Aradu.A09:42017748-42018099	22.76	0.79	-4.84	Ribosomal S15 (Chloroplast)
Aradu.AYN79	Aradu.A04:48207858-48235908	103.85	3.63	-4.84	NAD(P)-Binding Rossmann-Fold
Aradu.03ENG	Aradu.A02:84440367-84441960	3303.52	118.14	-4.81	Lipid Transfer
Aradu.15WFY	Aradu.A04:76875156-76879733	3.15	0.11	-4.80	Phosphatidylinositol:Ceramide Inositolphosphotransferase 1
Aradu.671CI	Aradu.A04:8048664-8055502	5.57	0.20	-4.76	Probable Lrr Receptor-Like Serine Threonine- Kinase
Aradu.SMZ6S	Aradu.A05:51450939-51458617	6.54	0.24	-4.76	Bromodomain-Containing Factor 1-Like Isoform X2
Aradu.6U69W	Aradu.A03:96524242-96525179	7.94	0.30	-4.72	Nadh-Plastoquinone Oxidoreductase Subunit K (Chloroplast)
Aradu.R7QS7	Aradu.A09:61406801-61408539	7.32	0.29	-4.65	Nadh-Plastoquinone Oxidoreductase Subunit 7 (Chloroplast)
Aradu.025HX	Aradu.A01:90958907-90961295	16.11	0.66	-4.60	Ribosomal S12 (Chloroplast)
Aradu.NP81U	Aradu.A02:93016336-93019724	2.62	0.11	-4.60	Metacaspase-1-Like
Aradu.S1EHV	Aradu.A04:30101833-30104322	20.23	0.84	-4.59	DNA Mismatch Repair Msh6
Aradu.YH2WE	Aradu.A02:43071569-43072534	34.99	1.47	-4.58	Ribosomal S12 (Chloroplast)
Aradu.92LSD	Aradu.A06:17939131-17942134	62.18	2.75	-4.50	Hypothetical Protein Phavu_003G055000G
Aradu.WL7RN	Aradu.A05:82815040-82818057	16.76	0.75	-4.49	Predicted: Uncharacterized Protein Loc104211086, Partial
Aradu.ZKQ4D	Aradu.A01:86347234-86349001	2.87	0.13	-4.42	Atp Synthase Cf0 Subunit Iv (Chloroplast)
Aradu.8Q8DX	Aradu.A05:34039969-34041388	6.98	0.33	-4.42	E3 Ubiquitin- Ligase Rf298

Aradu.E0DEN	Aradu.A03:120321820-120322931	2.96	0.14	-4.41	Hypothetical Chloroplast Rf2 (Chloroplast)
Aradu.24J3X	Aradu.A02:76052839-76061194	1.75	0.09	-4.36	6B-Interacting 2
Aradu.15REA	Aradu.A09:93730993-93737283	3.79	0.19	-4.34	Isoprene Partial
Aradu.413LK	Aradu.A09:13531291-13534141	1.97	0.10	-4.32	Photosystem Ii D2 (Chloroplast)
Aradu.0X4JL	Aradu.A03:57744010-57745584	8.46	0.43	-4.31	Maturase K (Chloroplast)
Aradu.HK886	Aradu.A02:78847815-78858979	0.42	0.02	-4.25	Diphthamide Biosynthesis 1
Aradu.8H8DD	Aradu.A01:11549548-11554953	15.28	0.81	-4.24	Photosystem I P700 Apo A2 (Chloroplast)
Aradu.42JCM	Aradu.A03:123376135-123376969	4.45	0.25	-4.17	Hypothetical Chloroplast Rf19 (Chloroplast)
Aradu.DY203	Adur2007:17515-19406	3.37	0.19	-4.16	NAD(P)H-Quinone Oxidoreductase Subunit H
Aradu.32P5Q	Aradu.A05:34042346-34044434	13.02	0.76	-4.10	Chloroplastic
Aradu.GFT6J	Aradu.A03:132380091-132380966	5.94	0.35	-4.08	Tify 5A
Aradu.F6FA7	Adur3089:77-407	97.33	5.84	-4.06	Ribosomal S7 (Chloroplast)
Aradu.N5Y0T	Adur1570:46524-48052	6.31	0.38	-4.04	Maturase (Chloroplast)
Aradu.79WAC	Aradu.A07:64286291-64290975	13.71	0.89	-3.95	Ribulose-1,5-Bisphosphate Carboxylase Oxygenase Large
Aradu.JIY90	Aradu.A02:74896147-74896740	18.23	1.20	-3.92	NADH
Aradu.AF71L	Adur1943:20552-21989	22.91	1.52	-3.91	ATP Synthase Cf1 Alpha Subunit (Chloroplast)
Aradu.4VP01,Ara	Aradu.A03:64921662-64923153	51.26	3.45	-3.89	NADH-Plastoquinone Oxidoreductase Subunit I (Chloroplast)
Aradu.JM7KB	Aradu.A06:7070983-7073818	1.13	0.08	-3.89	Probable Glycerol-3-Phosphate Acyltransferase 2-Like
Aradu.YJB40	Aradu.A04:40019117-40020959	1.66	0.11	-3.88	Photosystem I P700 Chlorophyll A Apo A1 (Chloroplast)
Aradu.XF675	Aradu.A01:32780195-32780752	82.71	5.68	-3.86	Atpase Alpha Partial
Aradu.WBM86	Aradu.A08:8771409-8778254	4.79	0.34	-3.82	Oxidoreductase Nad-Binding Rossmann Fold
Aradu.Y3QBI	Aradu.A05:100316307-100325967	4.66	0.33	-3.81	Alpha-Dioxygenase 1-Like
Aradu.1W5FD	Aradu.A02:7550917-7554786	4.58	0.33	-3.81	Bon1-Associated 2-Like
Aradu.32RQ0	Aradu.A06:1334862-1335687	5.97	0.47	-3.68	Ring-H2 Finger Atl3
Aradu.75VJN	Aradu.A02:88586681-88592824	14.94	1.17	-3.68	Disease Resistance (Tir-Nbs-Lrr Class)
Aradu.8WP5Z	Aradu.A09:9513780-9515534	15.22	1.24	-3.62	Duf506 Family
Aradu.WX3Q6	Aradu.A03:57703558-57706597	5.37	0.44	-3.60	Atp Synthase Cf1 Alpha Subunit (Chloroplast)
Aradu.3TG3W	Aradu.A09:42014650-42017056	5.44	0.45	-3.58	Partial (Chloroplast)
Aradu.V83WY	Aradu.A09:40951589-40952069	27.44	2.29	-3.58	Hypothetical Chloroplast Rf1 (Chloroplast)
Aradu.EAG5M	Aradu.A02:7054740-7058037	1.04	0.09	-3.58	Vacuolar-Sorting Receptor 1-Like
Aradu.6E94N	Aradu.A01:106256060-106260643	15.98	1.37	-3.54	Bromodomain-Containing Factor 1-Like Isoform X2
Aradu.U8TGW	Aradu.A05:9005069-9007455	3.29	0.28	-3.54	Lysm Domain Receptor-Like Kinase 4
Aradu.SL2ND	Aradu.A03:3850167-3855343	7.51	0.65	-3.53	Extra-Large Guanine Nucleotide-Binding 1-Like

Aradu.S88B1	Aradu.A01:26902718-26904142	587.24	50.88	-3.53	Cytochrome B Reductase 1
Aradu.A4MFG	Aradu.A01:106021641-106034380	32.33	2.81	-3.52	Hypothetical_ (Chloroplast)
Aradu.AB4DD	Aradu.A05:31687169-31687460	41.61	3.66	-3.51	Ribosomal S12 (Chloroplast)
Aradu.RU8HS	Aradu.A10:104125539-104130118	4.96	0.44	-3.51	Late Embryogenesis Abundant
Aradu.GXJ7L	Aradu.A03:150901-152588	4.25	0.37	-3.50	Pollen Ole E 1 Allergen And Extensin Family
Aradu.D8UNY	Aradu.A05:82654463-82656303	3.04	0.27	-3.50	Peroxidase 47
Aradu.MSL3N	Aradu.A03:17262791-17263975	2.65	0.23	-3.50	Myb-Related Myb4
Aradu.8KH6E	Aradu.A03:122541382-122553782	2.82	0.25	-3.49	U-Box Domain-Containing 21-Like
Aradu.77SYB	Aradu.A03:37374155-37375298	374.53	33.60	-3.48	Hypothetical_ (Chloroplast)
Aradu.K3VD2	Aradu.A02:33823187-33823813	8.21	0.74	-3.48	Ribosomal S3 (Chloroplast)
Aradu.BX0IC	Aradu.A02:7424229-7424736	24.28	2.18	-3.48	Bon1-Associated 2-Like
Aradu.61DK7	Aradu.A06:17539817-17540250	190.34	17.23	-3.47	Cell Wall-Associated Hydrolase
Aradu.V72Z0	Aradu.A06:96456773-96460037	1.41	0.13	-3.45	Predicted: Uncharacterized Protein Loc105043294 Isoform X1
Aradu.PEA62	Aradu.A10:30590205-30600505	8.04	0.74	-3.45	Replication Factor-A Carboxy-Terminal Domain
Aradu.420FT	Aradu.A06:52571873-52574703	8.73	0.80	-3.45	Predicted: Uncharacterized Protein Loc100814311
Aradu.RA346	Aradu.A02:75888435-75892373	13.66	1.26	-3.43	L-Ala-D L-Glu Epimerase
Aradu.XIP04	Aradu.A10:43343119-43346547	1.34	0.12	-3.42	Probable Lrr Receptor-Like Serine Threonine- Kinase At3G47570
Aradu.M3XI9	Aradu.A06:17666121-17670693	1.13	0.11	-3.42	Gdsl-Like Lipase Acylhydrolase
Aradu.F8RAG	Aradu.A09:90320707-90323526	2.89	0.27	-3.40	Myb-Related Myb4
Aradu.3V07T	Aradu.A06:100882110-100884662	5.12	0.48	-3.40	Phosphoglycerate Mutase Family
Aradu.H1E2F	Adur97_2:39261-40585	11.11	1.05	-3.40	Cytochrome B Reductase 1
Aradu.WQX7T	Aradu.A09:111463057-111465064	21.09	2.00	-3.40	Cytochrome B Reductase 1
Aradu.U1FLK	Aradu.A04:110970897-110974132	4.15	0.40	-3.36	Nuclease Harbi1-Like
Aradu.CI98G	Aradu.A02:86487922-86490739	1.38	0.14	-3.35	LRR Receptor-Like Kinase Family
Aradu.W09PA	Aradu.A01:1925333-1928482	41.96	4.14	-3.34	Ribulose 1,5-Bisphosphate Carboxylase Oxygenase Large Subunit
Aradu.ZXE5G	Aradu.A06:34293841-34296911	3.89	0.39	-3.33	Pyruvate Dehydrogenase E1 Component Subunit Mitochondrial
Aradu.3YG82	Aradu.A03:13122727-13128044	5.54	0.55	-3.33	NADH-Plastoquinone Oxidoreductase Subunit Partial
Aradu.U5WMC	Aradu.A02:43072743-43075675	2.05	0.20	-3.32	NADH Dehydrogenase Subunit 2 (Chloroplast)
Aradu.EF37G	Aradu.A01:8598438-8600820	9.14	0.91	-3.32	Abscisic Acid 8 -Hydroxylase
Aradu.G4NCZ	Aradu.A03:120064927-120067376	7.69	0.77	-3.32	Rac-Like Gtp-Binding Rac13
Aradu.2QX0T	Aradu.A08:45390368-45392781	16.49	1.66	-3.31	Isoflavone 7-O-Methyltransferase-Like
Aradu.T2SCC	Aradu.A06:73560058-73564419	24.56	2.51	-3.29	Serine Threonine- Kinase
Aradu.DF5FL	Aradu.A08:17130457-17134361	1.75	0.18	-3.29	Endo-1,4-Beta-Xylanase A

Aradu.3219N	Aradu.A07:39747901-39749132	2.90	0.30	-3.28	Photosystem I P700 Apo A1
Aradu.GG3LG	Aradu.A06:109586436-109590897	3.94	0.41	-3.28	Ap2-Like Ethylene-Responsive Transcription Factor Ant
Aradu.V0PXB	Aradu.A06:2989336-2992566	6.89	0.71	-3.28	Coatomer Subunit Beta -2-Like Isoform X1
Aradu.D91GM	Aradu.A09:4189033-4197962	1.62	0.17	-3.28	Leucine-Rich Repeat Receptor Kinase
Aradu.T1Z0F	Aradu.A04:110959001-110961137	0.92	0.10	-3.27	Oligopeptide Transporter 4
Aradu.QYF40	Adur2351:10084-13595	5.42	0.57	-3.25	Rna Polymerase Beta Subunit (Chloroplast)
Aradu.0HC1A	Aradu.A03:125418027-125419817	4.40	0.47	-3.24	Clavaminate Synthase At3G21360
Aradu.M9E5N	Aradu.A07:13482770-13483778	26.02	2.81	-3.21	Cell Wall-Associated Partial
Aradu.JJU8Z	Aradu.A07:6738238-6741296	18.25	1.97	-3.21	Receptor Kinase
Aradu.KG41H	Aradu.A06:2586564-2587836	27.03	2.94	-3.20	Wrky Transcription Factor 30
Aradu.81UZ4	Aradu.A04:468565-470025	1.47	0.16	-3.19	Pentatricopeptide Repeat-Containing Mitochondrial-Like
Aradu.DV5XC	Aradu.A08:46505942-46511354	2.86	0.31	-3.18	Zinc Finger Mym-Type 1-Like
Aradu.L67UJ	Aradu.A01:71934807-71940341	5.28	0.58	-3.18	Hmg (High Mobility Group) Box
Aradu.0LH9P	Aradu.A10:51186922-51209602	391.90	43.46	-3.17	Senescence-Associated
Aradu.EU1QI	Aradu.A10:54444733-54446119	232.25	25.80	-3.17	Rrna Intron-Encoded Homing Endonuclease
Aradu.K88X4	Aradu.A04:40043911-40044424	9.99	1.11	-3.17	Photosytem I Subunit Vii
Aradu.8I4EW	Aradu.A03:112487260-112489047	1.82	0.20	-3.16	Periodic Tryptophan 1 Homolog
Aradu.UGD4H	Aradu.A10:54490150-54491409	213.15	23.92	-3.16	Senescence-Associated Partial
Aradu.YY5C4	Aradu.A08:11204308-11210110	1.48	0.17	-3.15	Auxin-Induced 5Ng4
Aradu.B2QV1	Aradu.A01:6984325-6988868	20.18	2.29	-3.14	26S Proteasome Non-Atpase Regulatory Subunit 4
Aradu.023N4	Aradu.A03:38984267-38987093	1.01	0.12	-3.13	Transcription Factor Bhlh041
Aradu.73Q5I	Aradu.A06:101041470-101045618	4.56	0.52	-3.13	Structural Maintenance Of Chromosomes 2-1-Like
Aradu.GD6AN	Aradu.A03:34991420-34993549	2.05	0.23	-3.13	Calmodulin Binding -
Aradu.RHX7G	Aradu.A08:33549917-33554798	0.83	0.10	-3.12	Disease Resistance (Tir-Nbs-Lrr Class)
Aradu.YD5BQ	Aradu.A07:2403015-2408062	1.21	0.14	-3.12	Indole-3-Acetate O-Methyltransferase 1
Aradu.95FNB	Aradu.A06:95759474-95762811	3.48	0.40	-3.12	Nadh-Plastoquinone Oxidoreductase Subunit 5 (Chloroplast)
Aradu.FJ2EW	Aradu.A09:80446159-80448302	54.87	6.33	-3.12	Senescence-Associated
Aradu.NTZ01	Aradu.A07:14971543-14972336	4.55	0.53	-3.11	Jq0280 Hypothetical 12K (Trna Intron) - Rice Chloroplast
Aradu.T5GD5	Aradu.A09:42002793-42003165	43.76	5.11	-3.10	Photosystem Ii D2
Aradu.97XXG	Aradu.A09:80446159-80448302	51.52	6.03	-3.10	ATP Synthase Subunit Beta
Aradu.DK48U	Aradu.A06:53515425-53519110	18.17	2.15	-3.08	Peroxisome Biogenesis 6
Aradu.M3CK1	Aradu.A08:40383259-40394965	10.10	1.23	-3.04	Homogentisate Phytyltransferase Chloroplastic-Like Isoform X2
Aradu.85352	Aradu.A08:14623355-14626640	48.50	5.91	-3.04	Conserved

Aradu.6JX3E	Aradu.A03:20327681-20331612	322.58	39.53	-3.03	Surfeit Locus
Aradu.99525	Aradu.A03:37378015-37379764	3.19	0.39	-3.03	Envelope Membrane (Chloroplast)
Aradu.8G53Y	Aradu.A09:97857374-97858588	11.67	1.44	-3.01	Ribosomal L23 (Chloroplast)
Aradu.BW81J	Adur97_2:37950-38452	27.46	3.41	-3.01	Atp Synthase Cf1 Epsilon Subunit (Chloroplast)
Aradu.L6GSR	Aradu.A09:109120205-109121800	1.69	0.21	-3.00	Probable Membrane-Associated Kinase Regulator 5
Aradu.YNT13	Adur2001:18-994	6.62	0.83	-2.99	Hypothetical Chloroplast Rf1 (Chloroplast)
Aradu.ZX7AV	Aradu.A10:106783263-106792098	0.90	0.11	-2.99	Uncharacterized Protein Loc100777206 Isoform X4
Aradu.N5Q48	Aradu.A03:125815754-125821546	2.58	0.33	-2.99	NADH-Plastoquinone Oxidoreductase Subunit 4 (Chloroplast)
Aradu.11T9C	Aradu.A03:3109492-3111324	17.44	2.20	-2.99	Coatomer Subunit Beta -2 Isoform X2
Aradu.HG560	Aradu.A01:90965348-90965692	14.28	1.83	-2.96	Cell Wall-Associated Hydrolase
Aradu.2DM5J	Aradu.A06:12972043-12974106	0.64	0.08	-2.96	Ankyrin Repeat Domain-Containing 65-Like
Aradu.Z0RM4	Aradu.A03:57721826-57722198	113.81	14.63	-2.96	Hypothetical_ (Chloroplast)
Aradu.DZY1X	Aradu.A08:36370543-36376452	6.48	0.83	-2.96	Uncharacterized Protein Loc105043294 Isoform X1
Aradu.S4XSK	Aradu.A05:91090286-91093086	0.89	0.11	-2.96	Rho Guanine Nucleotide Exchange Factor 8-Like
Aradu.VI45B	Aradu.A07:7214053-7218889	4.78	0.62	-2.95	Coatomer Subunit Beta -2-Like Isoform X1
Aradu.9I2RZ	Aradu.A09:40954192-40955259	1.97	0.26	-2.93	Hypothetical Chloroplast Rf21 (Chloroplast)
Aradu.VL3IZ	Aradu.A08:8671727-8673376	1.19	0.16	-2.91	Probable Wrky Transcription Factor 53
Aradu.ZB3Q7	Aradu.A02:23446644-23452401	14.42	1.93	-2.90	Tbc1 Domain Family Member 15-Like
Aradu.T6VPT	Aradu.A01:96057232-96059743	1.67	0.22	-2.90	Cobra 7
Aradu.UDK2M	Aradu.A06:95592140-95594966	1.23	0.17	-2.90	Chaperone 1
Aradu.D6H9Q	Aradu.A03:37371568-37374110	74.50	10.09	-2.88	Photosystem I P700 Apo A1 (Chloroplast)
Aradu.D8WVG	Aradu.A10:4724996-4728538	24.49	3.32	-2.88	Proline Iminopeptidase
Aradu.M8IZW	Aradu.A10:10708749-10710803	3.63	0.49	-2.88	Sufe Chloroplastic Isoform X1
Aradu.5YW6Q	Aradu.A04:18191607-18192090	83.48	11.44	-2.87	Hypothetical Protein, Partial
Aradu.DB48N	Aradu.A05:31686294-31686716	15.72	2.18	-2.85	Chromosome Genomic Cultivar Chinese Spring
Aradu.U260V	Aradu.A09:3752718-3756699	34.04	4.73	-2.85	Gdsl Esterase Lipase Apg-Like
Aradu.HF6QX	Aradu.A03:33786443-33787001	8.33	1.16	-2.84	Nim1-Interacting 2-Like
Aradu.BH653	Aradu.A07:2748885-2749827	16.07	2.24	-2.84	Heterodimeric Geranylgeranyl Pyrophosphate Synthase
Aradu.P7MGC	Aradu.A06:30910440-30912960	4.20	0.59	-2.84	Sulfate Transporter -Like
Aradu.2EU9J	Aradu.A10:106879157-106881701	5.46	0.77	-2.83	Adp-Ribosylation Factor Gtpase-Activating Agd12
Aradu.CL7N4	Aradu.A05:20063361-20066123	3.72	0.53	-2.81	5-Methyltetrahydropteroyltriglutamatecysteine Methyltransferase
Aradu.V4UNF	Aradu.A07:13474421-13477569	3.51	0.50	-2.81	Photosystem II D2
Aradu.ZF5BJ	Aradu.A06:91579530-91581213	20.00	2.86	-2.80	Hypothetical Chloroplast Rf68

Aradu.NL01W	Aradu.A04:3742606-3749174	0.94	0.14	-2.80	Mediator Of Rna Polymerase Ii Transcription Subunit 33A-Like
Aradu.3R3ED	Aradu.A02:15229538-15255205	3.91	0.56	-2.80	Uncharacterized Protein Loc100800997 Isoform X3
Aradu.0X4TI	Aradu.A10:103575382-103581322	1.12	0.16	-2.79	Calcium-Dependent Kinase 1-Like
Aradu.1X84T	Aradu.A10:5491603-5492491	10.50	1.54	-2.77	Exordium-Like 2
Aradu.MA01V	Aradu.A03:7523326-7525001	26.80	3.96	-2.76	Ubiquitin-Conjugating Enzyme E2 20-Like
Aradu.QH692	Aradu.A06:70048485-70049133	23.11	3.42	-2.76	Photosystem I Assembly Ycf4 (Chloroplast)
Aradu.L4IHQ	Aradu.A01:90965984-90966505	548.14	81.48	-2.75	Ribulose Bisphosphate Carboxylase Large Chain
Aradu.P3X2X	Aradu.A04:106287966-106289234	5.72	0.85	-2.75	Hypothetical Protein Glysoja_037341
Aradu.A3TK2	Aradu.A10:43437138-43438412	12.38	1.85	-2.74	Small Heat Shock Chloroplastic
Aradu.K52PG	Aradu.A05:5173973-5174219	58.95	8.85	-2.74	Atp Synthase Cf0 C Subunit (Chloroplast)
Aradu.B9597	Aradu.A03:130073940-130080687	4.96	0.75	-2.73	Polygalacturonase Inhibitor
Aradu.NY9NH	Aradu.A06:105676566-105679680	2.81	0.43	-2.72	Wall-Associated Receptor Kinase
Aradu.W5GQC	Aradu.A03:621531-627547	7.68	1.19	-2.68	Argonaute Family
Aradu.5VY47	Aradu.A08:47683590-47685477	17.93	2.79	-2.68	Dof Zinc Finger -Like Isoform X1
Aradu.A79DM	Aradu.A03:57722252-57726037	134.06	20.96	-2.68	Hypothetical Protein Glysoja_046181
Aradu.UA4I1	Aradu.A03:57737163-57738459	5.38	0.84	-2.67	Atp Synthase Cf1 Beta Subunit (Chloroplast)
Aradu.85609	Aradu.A07:47208143-47210648	24.52	3.85	-2.67	Photosystem I P700 Apo A2 (Chloroplast)
Aradu.Y27NY	Aradu.A05:508033-512988	9.57	1.51	-2.66	Glycosyltransferase Family
Aradu.3N4PQ	Aradu.A07:3703202-3710390	1.78	0.28	-2.66	Probable Lrr Receptor-Like Serine Threonine- Kinase
Aradu.X4R7M	Aradu.A01:89238161-89239959	3.83	0.61	-2.64	Tify 10A-Like
Aradu.08UMR	Aradu.A06:75839262-75842616	1.53	0.25	-2.64	Ovate Family
Aradu.YA6WS	Aradu.A05:52840092-52842934	10.66	1.72	-2.63	Expansin-A15-Like Precursor
Aradu.H0G14	Aradu.A06:107845767-107848867	7.60	1.24	-2.61	12-Oxophytodienoate Reductase 2-Like
Aradu.XQZ5U	Aradu.A09:59187052-59188460	4.20	0.69	-2.61	Exordium-Like 5
Aradu.96H72	Aradu.A04:5868390-5875626	5.08	0.83	-2.61	Transposase En Spm
Aradu.2W676	Aradu.A04:100934139-100936232	3.75	0.61	-2.61	Cationic Peroxidase 1-Like
Aradu.ZC0QI	Aradu.A10:54456725-54468094	99.08	16.25	-2.61	Senescence-Associated
Aradu.T37GN	Aradu.A05:6419784-6428911	5.22	0.86	-2.61	Calcium-Transporting Atpase Plasma Membrane-Type
Aradu.J2LS8	Aradu.A02:1869796-1874790	0.61	0.10	-2.61	Disease Resistance At3G14460-Like
Aradu.78NGX	Aradu.A04:96430081-96436578	14.29	2.35	-2.60	Nucleic Acid Binding
Aradu.GB0R9	Aradu.A08:43235904-43242231	0.45	0.07	-2.60	Tmv Resistance N-Like
Aradu.1F1FD	Aradu.A04:16981209-16981650	31.71	5.24	-2.60	Ring-H2 Finger Atl2-Like
Aradu.SEI26	Aradu.A05:39094443-39095193	388.24	64.44	-2.59	F-Box Skip22-Like

Aradu.JK7IR	Aradu.A10:15976405-15979129	292.34	48.56	-2.59	Hypothetical Protein Glysoja_046181
Aradu.61A42	Aradu.A01:104549223-104551190	23.12	3.85	-2.59	Cytochrome P450 78A5-Like
Aradu.HB0SB	Aradu.A10:2486097-2497899	10.36	1.75	-2.57	Mitochondrial Ribosome-Associated Gtpase 1 Isoform X3
Aradu.XCD6I	Aradu.A05:7133508-7141484	6.12	1.03	-2.57	Isoflavone 2 -Hydroxylase
Aradu.P709D	Aradu.A07:7498846-7502778	0.95	0.16	-2.56	Gdsl Esterase Lipase At2G04570-Like
Aradu.5JL10	Aradu.A09:1869627-1890387	24.82	4.21	-2.56	Leucine-Rich Repeat Receptor-Like Serine Threonine Tyrosine- Kinase
Aradu.YS103	Aradu.A03:27507226-27515451	5.93	1.01	-2.56	Glycerophosphoryl Diester Phosphodiesterase 3
Aradu.KZW97	Aradu.A03:32431542-32432007	34.98	5.98	-2.55	Arabinogalactan Peptide 22-Like
Aradu.71A1E	Aradu.A07:32675222-32676783	1.97	0.34	-2.55	NADH Dehydrogenase Subunit 2 (Chloroplast)
Aradu.7QZ7K	Aradu.A07:42834987-42836735	2.39	0.41	-2.55	NAD(P)-Binding Rossmann-Fold
Aradu.77ZLW	Aradu.A10:5944901-5947659	1.32	0.23	-2.54	Laccase-14
Aradu.9DS9L	Aradu.A04:42665420-42692355	148.56	25.61	-2.54	Senescence-Associated Partial
Aradu.67T23	Aradu.A02:21731391-21732041	3.09	0.53	-2.53	Cytochrome F (Chloroplast)
Aradu.42UNZ	Aradu.A01:88440965-88450093	2.77	0.48	-2.53	Splicing Regulatory Glutamine Lysine-Rich 1-Like
Aradu.6S4B3	Aradu.A02:4404973-4405951	6.23	1.08	-2.53	Permease Transmembrane
Aradu.ZU011	Aradu.A04:4776902-4780791	3.02	0.52	-2.53	F-Box Plant
Aradu.EJA5A	Aradu.A03:12674636-12678257	2.80	0.49	-2.52	Aluminum-Activated Malate Transporter 2
Aradu.618KY	Aradu.A01:81960127-81962501	6.20	1.09	-2.52	Nadh-Plastoquinone Oxidoreductase Subunit 4 (Chloroplast)
Aradu.9G9GJ	Aradu.A01:97073759-97076821	12.43	2.18	-2.51	Casp Vit_05S0020G01820
Aradu.2BI47	Aradu.A01:18056573-18060249	8.88	1.56	-2.51	Peroxidase 73
Aradu.84CRR	Aradu.A01:60233005-60233716	27.93	4.92	-2.50	Cell Wall-Associated Hydrolase
Aradu.5K7BE	Aradu.A05:97210770-97214274	14.85	2.62	-2.50	Purple Acid Phosphatase 17-Like
Aradu.I35R0	Aradu.A02:660260-660789	71.33	12.61	-2.50	Multispecies: Rnaase
Aradu.1MC5F	Aradu.A09:55264823-55272005	3.49	0.62	-2.50	Aspartic Ase 2
Aradu.JG4GI	Aradu.A06:111201205-111206886	1.35	0.24	-2.49	Patatin-Like Phospholipase
Aradu.83N8C	Aradu.A01:23818825-23821610	1.28	0.23	-2.46	Transcription Factor Bhlh18-Like
Aradu.UFC3R	Aradu.A02:7167843-7173551	5.06	0.92	-2.46	Acid Phosphatase 1-Like
Aradu.QL7M2	Aradu.A02:84934311-84936210	0.96	0.18	-2.45	Probable Inactive Receptor Kinase At5G58300
Aradu.TYN4X	Aradu.A10:104520837-104526027	4.06	0.74	-2.45	Leucine-Rich Repeat Receptor Kinase
Aradu.7X8N5	Adur93_2:49048-49596	6.93	1.27	-2.44	Leucine-Rich Repeat Receptor- Serine Threonine Tyrosine- Kinase
Aradu.4V3MR	Aradu.A03:123606514-123609297	1.36	0.25	-2.43	Receptor-Like Cytosolic Serine Threonine- Kinase Rbk1
Aradu.2GI2F	Aradu.A01:34940215-34940548	1044.08	193.92	-2.43	Cytochrome B Reductase 1
Aradu.I9K2A	Aradu.A04:121364675-121380589	12.78	2.38	-2.42	Isoflavone 7-O-Methyltransferase-Like

Aradu.FD4R7	Aradu.A06:16421561-16422038	5.70	1.07	-2.42	Ocs Element-Binding Factor 1-Like
Aradu.20TYF	Aradu.A10:87219664-87222096	6.50	1.22	-2.41	Wrky Transcription Factor 40
Aradu.IA6YI	Aradu.A05:91107751-91116638	0.74	0.14	-2.40	Pentatricopeptide Repeat-Containing At2G20540-Like
Aradu.GC3H9	Aradu.A03:96938428-96951506	8.00	1.52	-2.39	Uric Acid Degradation Bifunctional Ttl Isoform X2
Aradu.AE3JB	Aradu.A04:112215148-112226336	14.56	2.80	-2.38	Probable Receptor Kinase At5G24010
Aradu.W5PK0	Aradu.A09:118465464-118467834	2.76	0.53	-2.38	F-Box Skip19-Like
Aradu.H4688	Aradu.A10:32431296-32431997	8.91	1.74	-2.36	Photosystem Ii Reaction Center
Aradu.37P6F	Aradu.A04:47732590-47734168	8.29	1.62	-2.36	Leucine-Rich Repeat
Aradu.LNZ6T	Aradu.A03:104531550-104534243	1.28	0.25	-2.35	Squamosa Promoter-Binding 13
Aradu.AVR14	Aradu.A09:14865055-14867760	22.25	4.36	-2.35	Cytochrome P450 71A1-Like
Aradu.077V4	Aradu.A10:82228416-82229737	14.20	2.78	-2.35	23S Ribosomal Rna
Aradu.39MPT	Aradu.A04:121706092-121707958	72.49	14.35	-2.34	Plant-Specific Domain Tigr01589 Family
Aradu.K3NB7	Aradu.A07:43281253-43283608	0.63	0.13	-2.33	Duf946 Family
Aradu.U999X	Aradu.A06:10656035-10658785	2.07	0.42	-2.32	Cytokinin Dehydrogenase 1-Like
Aradu.A3AX6	Aradu.A01:2916051-2919650	9.34	1.88	-2.31	Aspartic Ase Cdr1-Like
Aradu.010SR	Aradu.A03:57722252-57726037	159.15	32.09	-2.31	Ycf68 Protein
Aradu.XT4RY	Aradu.A01:20822583-20823046	242.08	48.81	-2.31	Hypothetical Protein Mtr_3G035650
Aradu.HY1A1	Aradu.A01:99906837-99907534	3.85	0.78	-2.30	Probable Adp-Ribosylation Factor Gtpase-Activating Agd13
Aradu.750CN	Aradu.A01:22157247-22159551	1.42	0.29	-2.30	Probable Xyloglucan Endotransglucosylase Hydrolase 6-Like
Aradu.LCH2B	Aradu.A01:92275589-92277674	19.09	3.87	-2.30	Wat1-Related At4G30420-Like
Aradu.TBQ82	Aradu.A09:5380094-5385793	0.48	0.10	-2.28	Abc Transporter C Family Member 8-Like
Aradu.G8P6Y	Aradu.A10:97138119-97143983	3.25	0.67	-2.28	Agamous-Like Mads-Box Agl8-Like
Aradu.54M2C	Aradu.A10:1158910-1161000	1.67	0.34	-2.28	Receptor-Like Kinase
Aradu.E4KVL	Aradu.A05:51180223-51187848	0.72	0.15	-2.28	Csc1 Hyp1
Aradu.U8NZD	Aradu.A03:6655406-6657355	1.54	0.32	-2.27	Lignin-Forming Anionic Peroxidase
Aradu.IP5K7	Aradu.A03:1873376-1877001	28.87	6.00	-2.27	Mitogen-Activated Kinase 3
Aradu.I4MAR	Aradu.A06:95750202-95751925	797.16	165.79	-2.27	Hypothetical_ (Chloroplast)
Aradu.WH49R	Aradu.A05:89490485-89494378	4.30	0.90	-2.26	Receptor Kinase Hsl1-Like
Aradu.65JMT	Aradu.A10:89252771-89257904	14.03	2.92	-2.26	Dna Topoisomerase 2-Binding 1
Aradu.42WYD	Adur1855:20800-25578	2.07	0.43	-2.26	Cytochrome C Oxidase Subunit 1 (Mitochondrion)
Aradu.0YE33	Aradu.A01:96541524-96542582	486.82	102.00	-2.25	Late Embryogenesis Abundant Lea5
Aradu.DA77H	Aradu.A05:87784393-87786201	3.04	0.64	-2.25	P-Loop Nucleoside Triphosphate Hydrolase Superfamily
Aradu.C7EQY	Aradu.A02:84997126-85006265	14.39	3.04	-2.24	Replication Factor-A Carboxy-Terminal Domain

Aradu.D4Z5N	Aradu.A08:39776886-39778341	4.86	1.03	-2.24	Mlp 43
Aradu.Y5KV9	Aradu.A01:39812299-39815736	1.61	0.34	-2.24	Cell Cycle Checkpoint Rad1
Aradu.G29LA	Aradu.A09:7158099-7225871	36.95	7.82	-2.24	Tmv Resistance N-Like Isoform X1
Aradu.FE38M	Aradu.A08:37925166-37927060	4.09	0.87	-2.24	Basic Helix-Loop-Helix
Aradu.5Z6ML	Adur1683:19272-19800	222.31	47.29	-2.23	Atp Synthase Subunit Alpha
Aradu.ER7XF	Aradu.A08:293431-296077	2.29	0.49	-2.23	Gmp Synthase [Glutamine-Hydrolyzing]
Aradu.Z7Z26	Aradu.A06:70064133-70064739	14.15	3.02	-2.23	Photosystem I Assembly Ycf4 (Chloroplast)
Aradu.K3YJZ	Aradu.A05:93789817-93792818	1.23	0.26	-2.23	Probable Receptor Kinase At1G67000-Like Isoform X2
Aradu.C21WZ	Aradu.A06:2856935-2861500	8.25	1.77	-2.22	Programmed Cell Death 4
Aradu.F529W	Aradu.A05:553978-568114	18.09	3.92	-2.21	Mannitol Dehydrogenase
Aradu.AU70V	Aradu.A02:91475445-91479422	1.26	0.27	-2.20	Probable Carboxylesterase 15
Aradu.C6WK8	Aradu.A04:8461201-8464483	1.11	0.24	-2.20	Mate Efflux Family Alf5
Aradu.C826W	Aradu.A04:31440998-31447316	0.74	0.16	-2.20	Root Hair Defective 3 Homolog 2-Like
Aradu.IY6CW	Aradu.A09:52719866-52720805	36.37	7.94	-2.19	Cell Wall-Associated Hydrolase
Aradu.D3M3F	Aradu.A03:114871376-114874881	0.92	0.20	-2.18	L-Ascorbate Oxidase
Aradu.0746P	Aradu.A09:29848007-29848977	47.73	10.52	-2.18	Bnac09G29270D
Aradu.ZH9NQ	Aradu.A03:7879331-7882714	1.50	0.33	-2.18	Aspartyl Glutamyl-Trna(Asn Gln) Amidotransferase
Aradu.1L0P8	Aradu.A07:16137238-16141228	2.13	0.47	-2.18	P-Loop Nucleoside Triphosphate Hydrolase Superfamily
Aradu.S5T1J	Aradu.A03:1121270-1125312	0.68	0.15	-2.18	P-Loop Containing Nucleoside Triphosphate Hydrolases
Aradu.7PC3U	Aradu.A01:54164221-54166339	9.76	2.16	-2.18	Senescence-Inducible Chloroplast Stay-Green
Aradu.9V11P	Aradu.A01:94219932-94221470	33.54	7.42	-2.18	Blue Copper -Like
Aradu.Z49Y7	Aradu.A04:74983250-74987156	5.53	1.23	-2.17	Plant T7H20-70
Aradu.4V5VR	Aradu.A03:10195282-10197065	1.20	0.27	-2.16	Photosystem Ii 47 Kda (Chloroplast)
Aradu.EX30Z	Aradu.A08:6978695-6982189	18.25	4.08	-2.16	Metal-Nicotianamine Transporter Ysl3
Aradu.XD7VB	Aradu.A08:42251577-42253460	4.12	0.92	-2.16	Zinc Finger 512B
Aradu.0Q24I	Aradu.A05:93751868-93756347	4.35	0.98	-2.15	Calmodulin Binding -
Aradu.0J6QE	Aradu.A07:13533922-13534591	21.82	4.90	-2.15	Photosystem I P700 Chlorophyll A Apo Partial (Chloroplast)
Aradu.U2XYQ	Aradu.A08:33725604-33742269	1.69	0.38	-2.15	Disease Resistance (Tir-Nbs-Lrr Class)
Aradu.4R68R	Aradu.A05:26694177-26697542	4.54	1.02	-2.15	Methylsterol Monooxygenase 2-2
Aradu.VPR7N	Aradu.A06:14696881-14699842	1.01	0.23	-2.14	Alg-2 Interacting X-Like
Aradu.665TV	Aradu.A08:24203714-24207550	2.56	0.58	-2.14	G2 Mitotic-Specific Cyclin S13-7-Like
Aradu.9M8WB	Aradu.A01:6999881-7004668	6.74	1.53	-2.14	Transcription Factor Bhlh93
Aradu.XU9GE	Aradu.A08:11467297-11470621	3.25	0.74	-2.14	Auxin Transporter 3

Aradu.M0NEF	Aradu.A10:63788470-63793096	21.04	4.80	-2.13	Lysine Methyltransferase Mettl21D Isoform X1
Aradu.L2ZIJ	Aradu.A03:113255556-113259418	4.23	0.97	-2.13	Hexokinase- Chloroplastic
Aradu.Z9E7L	Aradu.A06:93206770-93210875	26.68	6.10	-2.13	Iaa-Amino Acid Hydrolase Ilr1-Like 4
Aradu.WDP7Q	Aradu.A03:110222865-110228463	0.72	0.17	-2.12	Fatty Acid Amide Hydrolase
Aradu.S3RLK	Aradu.A02:78861196-78865270	0.42	0.10	-2.12	Diphthamide Biosynthesis 1
Aradu.S3UJF	Aradu.A03:2328383-2330519	3.63	0.84	-2.12	Ras-Related Rabe1C
Aradu.CZ7XQ	Aradu.A08:37147874-37149258	2.92	0.68	-2.11	Stem-Specific Tsjt1-Like
Aradu.TKG8T	Aradu.A06:110422166-110424136	4.00	0.93	-2.11	S-Adenosyl-L-Methionine-Dependent Methyltransferases
Aradu.B9QD5	Aradu.A10:3776298-3796816	0.80	0.18	-2.11	G-Type Lectin S-Receptor-Like Serine Threonine- Kinase
Aradu.SJ7I8	Aradu.A09:4543925-4548924	2.63	0.61	-2.10	Anoctamin At1G73020 Isoform X1
Aradu.FZ6D2	Aradu.A05:93963641-93967404	6.40	1.50	-2.09	Probable Serine Threonine- Kinase At1G18390 Isoform X2
Aradu.UQA95	Aradu.A06:5861513-5865642	2.28	0.54	-2.09	Encodes Alpha-Helical If Isoform 1
Aradu.5Z359	Aradu.A05:1312575-1315675	7.20	1.71	-2.07	Patatin 2
Aradu.ZQ4AK	Aradu.A02:21760834-21761896	54.69	12.99	-2.07	Photosystem Ii D1 (Chloroplast)
Aradu.3W36V	Aradu.A04:104338150-104345223	4.04	0.96	-2.07	Lrr Receptor-Like Serine Threonine- Kinase
Aradu.NBA8B	Aradu.A08:31525608-31537835	18.39	4.39	-2.07	Two Pore Calcium Channel 1
Aradu.39755	Aradu.A08:33469430-33471274	4.51	1.08	-2.07	Kdel-Tailed Cysteine Endopeptidase Cep1-Like
Aradu.MW4MQ	Aradu.A07:71152723-71153794	3.40	0.82	-2.06	Lactoylglutathione Lyase
Aradu.MU0PR	Aradu.A05:108212363-108228146	1.20	0.29	-2.06	Disease Resistance (Tir-Nbs-Lrr Class)
Aradu.0B0KP	Aradu.A01:106718273-106720916	7.04	1.69	-2.06	Hypersensitive-Induced Response 1
Aradu.5PB9J	Aradu.A06:110789181-110792365	1.74	0.42	-2.05	Ppr Superfamily
Aradu.US93A	Aradu.A09:10720238-10725411	6.32	1.53	-2.05	Nadh Dehydrogenase [Ubiquinone] 1 Alpha Subcomplex
Aradu.N441K	Aradu.A01:101265990-101274713	0.61	0.15	-2.05	Pleiotropic Drug Resistance 3-Like
Aradu.Y0R7I	Aradu.A04:16209288-16212326	0.84	0.20	-2.05	Catalytic Phosphatase Type 2C
Aradu.BX6BE	Aradu.A08:30519573-30521204	5.28	1.28	-2.04	Malonyl-Co A:Anthocyanin 3-Glucoside 6-Malonyltransferase
Aradu.V6MKI	Aradu.A09:105994877-106000165	1.19	0.29	-2.04	Pyruvate Cytosolic Isozyme-Like
Aradu.YLV00	Aradu.A03:846993-848902	34.95	8.54	-2.03	Ring Finger And Chy Zinc Finger Domain-Containing 1
Aradu.SD5C0	Aradu.A01:104452931-104456476	1.88	0.46	-2.03	Squamosa Promoter-Binding 6
Aradu.AG5X8	Aradu.A03:114053703-114055116	2.78	0.68	-2.03	Apo Mitochondrial
Aradu.KC4QF	Aradu.A10:70351738-70353779	11.31	2.77	-2.03	Inactive Kinase Selmodraft_444075-Like Isoform X1
Aradu.MK4GU	Aradu.A03:2158905-2160708	1.09	0.27	-2.03	3-Ketoacyl- Synthase 12
Aradu.N2AA8	Aradu.A02:32164846-32165195	154.05	37.88	-2.02	Ycf68
Aradu.QL20A	Aradu.A06:11888611-11891298	4.22	1.04	-2.02	Transforming Growth Factor-Beta Receptor-Associated 1

Aradu.CP3UH	Aradu.A02:88923822-88930409	12.87	3.17	-2.02	Phenazine Biosynthesis Family
Aradu.NE9UM	Aradu.A03:45778608-45782855	1.82	0.45	-2.02	Dna Repair
Aradu.BYP3X	Aradu.A10:7465572-7467281	2.65	0.66	-2.01	Embryonic Abundant Usp92
Aradu.JV4TN	Aradu.A04:100366877-100370299	1.65	0.41	-2.01	Coatomer Subunit Gamma
Aradu.SK9II	Aradu.A03:86708604-86712140	4.16	1.04	-2.01	Serine Threonine- Kinase At5G01020-Like Isoform X1
Aradu.H1J0M	Aradu.A05:93971862-93973599	1.60	0.40	-2.00	Probable Receptor Kinase At1G67000-Like Isoform X1
Aradu.IP2GS	Aradu.A07:73716822-73725731	4.06	1.01	-2.00	Glycine--Trna Ligase Mitochondrial-Like
Aradu.LD7BF	Aradu.A05:4635190-4636033	7.66	1.91	-2.00	Ethylene-Responsive Transcription Factor 1A-Like

Table S8: Associated pathways of differentially expressed genes during deficit irrigation stress in peanut

Pathway	Occurrence
Biosynthesis of antibiotics	23
Starch and sucrose metabolism	15
Phenylalanine, tyrosine and tryptophan biosynthesis	11
Amino sugar and nucleotide sugar metabolism	9
Cysteine and methionine metabolism	7
Flavonoid biosynthesis	7
Phenylalanine metabolism	6
Phenylpropanoid biosynthesis	6
Fructose and mannose metabolism	5
Galactose metabolism	5
Glycerophospholipid metabolism	5
Pentose and glucuronate interconversions	5
Tyrosine metabolism	5
Alanine, aspartate and glutamate metabolism	4
Ascorbate and aldarate metabolism	4
Carbon fixation in photosynthetic organisms	4
Glycerolipid metabolism	4
Isoquinoline alkaloid biosynthesis	4
Novobiocin biosynthesis	4
Purine metabolism	4
Pyruvate metabolism	4
Tropane, piperidine and pyridine alkaloid biosynthesis	4
alpha-Linolenic acid metabolism	3
Carotenoid biosynthesis	3
Drug metabolism - cytochrome P450	3
Fatty acid degradation	3
Glycine, serine and threonine metabolism	3
Glycolysis / Gluconeogenesis	3
Isoflavonoid biosynthesis	3
Metabolism of xenobiotics by cytochrome P450	3
Pentose phosphate pathway	3
Pyrimidine metabolism	3
Streptomycin biosynthesis	3
Terpenoid backbone biosynthesis	3
Aminobenzoate degradation	2
Arginine biosynthesis	2
Carbon fixation pathways in prokaryotes	2
Cutin, suberine and wax biosynthesis	2
Cyanoamino acid metabolism	2
Drug metabolism - other enzymes	2
Glucosinolate biosynthesis	2
Glycosaminoglycan biosynthesis - heparan sulfate/ h	2
Linoleic acid metabolism	2
Methane metabolism	2
Neomycin, kanamycin and gentamicin biosynthesis	2

Retinol metabolism	2
Riboflavin metabolism	2
Selenocompound metabolism	2
Sphingolipid metabolism	2
Steroid hormone biosynthesis	2
Stilbenoid, diarylheptanoid and gingerol biosynthesis	2
T cell receptor signaling pathway	2
Thiamine metabolism	2
Tryptophan metabolism	2
Valine, leucine and isoleucine biosynthesis	2
Valine, leucine and isoleucine degradation	2
Acarbose and validamycin biosynthesis	1
Aflatoxin biosynthesis	1
Aminoacyl-tRNA biosynthesis	1
Arachidonic acid metabolism	1
Arginine and proline metabolism	1
Benzoxazinoid biosynthesis	1
Biosynthesis of ansamycins	1
Biosynthesis of terpenoids and steroids	1
Biosynthesis of unsaturated fatty acids	1
Biosynthesis of vancomycin group antibiotics	1
Caffeine metabolism	1
Caprolactam degradation	1
Chlorocyclohexane and chlorobenzene degradation	1
Citrate cycle (TCA cycle)	1
Dioxin degradation	1
Diterpenoid biosynthesis	1
Ether lipid metabolism	1
Fatty acid biosynthesis	1
Fatty acid elongation	1
Fluorobenzoate degradation	1
Glutathione metabolism	1
Glycosaminoglycan biosynthesis - chondroitin sulfate /	1
Glycosaminoglycan degradation	1
Glycosphingolipid biosynthesis - ganglio series	1
Glycosphingolipid biosynthesis - globo and isoglobo s	1
Glycosphingolipid biosynthesis - lacto and neolacto se	1
Glyoxylate and dicarboxylate metabolism	1
Histidine metabolism	1
Indole alkaloid biosynthesis	1
Lipopolysaccharide biosynthesis	1
Lysine biosynthesis	1
Mannose type O-glycan biosynthesis	1
mTOR signaling pathway	1
Naphthalene degradation	1
One carbon pool by folate	1
Other glycan degradation	1
Pantothenate and CoA biosynthesis	1

Polycyclic aromatic hydrocarbon degradation	1
Polyketide sugar unit biosynthesis	1
Porphyrin and chlorophyll metabolism	1
Propanoate metabolism	1
Steroid biosynthesis	1
Sulfur metabolism	1
Taurine and hypotaurine metabolism	1
Th1 and Th2 cell differentiation	1
Toluene degradation	1
Ubiquinone and other terpenoid-quinone biosynthesis	1
Zeatin biosynthesis	1

Table S9: List of antibiotic synthesis pathway genes expressed during deficit irrigation (DI) stress in peanut

**Sequence name**	**Gene Id**	**Annotation**
XLOC_002120	*Aradu.ZI52D*	1-Deoxy-D-Xylulose-5-Phosphate Synthase
XLOC_007322	*Aradu.YZZ7F*	Acetylornithine Deacetylase
XLOC_008224	*Aradu.M7NEJ*	Bifunctional 3-Dehydroquinate Dehydratase Shikimate Chloroplastic-Like Isoform X1
XLOC_010816	*Aradu.L2ZIJ*	Hexokinase- Chloroplastic
XLOC_010859	*Aradu.GLM5E*	Bifunctional Aspartate Aminotransferase And Glutamate Aspartate-Prephenate Aminotransferase
XLOC_015240	*Aradu.MN6GR*	D-3-Phosphoglycerate Chloroplastic-Like
XLOC_018270	*Aradu.6SA52*	UDP-Glucuronic Acid Decarboxylase 6 Isoform X1
XLOC_018437	*Aradu.BL8NF*	Probable Ribose-5-Phosphate Isomerase 2
XLOC_018608	*Aradu.Z0G82*	Acetyl-Coenzyme A Carboxylase Carboxyl Transferase Subunit Chloroplastic-Like
XLOC_018880	*Aradu.NY5CH*	Chorismate Mutase Chloroplastic-Like
XLOC_022169	*Aradu.UL8XP*	3-Hydroxy-3-Methylglutaryl-Coenzyme A Reductase
XLOC_022911	*Aradu.32P5Q*	Chloroplastic
XLOC_027525	*Aradu.ZXE5G*	Pyruvate Dehydrogenase E1 Component Subunit Mitochondrial-Like
XLOC_029880	*Aradu.SB00U*	Phytoene Synthase Chloroplastic
XLOC_033352	*Aradu.348PZ*	Branched-Chain-Amino-Acid Aminotransferase Chloroplastic-Like Isoform X2
XLOC_039825	-	Probable Ribose-5-Phosphate Isomerase 2
XLOC_040555	-	Methionine Gamma-Lyase-Like
XLOC_043134	*Aradu.RK7RG*	Phospho-2-Dehydro-3-Deoxyheptonate Aldolase Chloroplastic-Like

Table S10: List of the primers used for qRT-PCR studies

S. No.	Gene Id	Gene name	Forward primer(5'-3')	Reverse primer (5'-3')
**1**	*Araip.982E5*	Ubiquitin- ligase	CCTTGCAGTATCATTCTCCGT	CAAATCAAATCGAACCCTGA
**2**	*Araip.9WJ7K*	NHL domain-containing isoform	AGTTGTTCCCTTGTGCTGTAAAT	GCCTATTTGCAAGACCACAAAA
**3**	*Araip.26ZIU*	Peptidyl-prolyl cis-trans isomerase	GCATAGCATGGATTTTCATAGTGAT	AGCAAATGGGCAAAAGATTA
**4**	*Araip.AD1F5*	LRR receptor serine threonine kinase	AGAAGTCAAATTCCCCAAAGC	CATCTGACTGGGACTATTTCTAATG
**5**	*Araip.J8WWD*	Vinorine synthase-like	CAATTCACTTATCTCAGTGTCCTTC	AGTGATGCTATGTGAGAATGTGAAG
**6**	*Aradu.96ASX*	Late embryogenesis abundant	CATCATAAACAGCATCTCTCAGACT	GTGAGGGATTGGCTCAACA
**7**	*Aradu.S1EHV*	DNA mismatch repair MSH6	TGAGAGTTTAAGCTACTGTTGAGGG	GGGGATGCTTTTGTTTATAATTCC
**8**	*Aradu.XF675*	ATPase alpha partial	TGGGTATTTCGGTTTCCAGA	TTGAGCCGCAGATCCAACT
**9**	*Aradu.77ZLW*	Laccase-14	CAGGTGCAGTGTTCAAACG	CCTCTGTGGTCAAATGGATAACTC
**10**	*Aradu.AGI8J*	Plastid movement impaired 2	TTGAATATATCCCAAGGCCAG	CATGTTAACAATGCAATCAAGGAC

Table S11: Allele specific primers used for the SNP identification during deficit irrigation stress in peanut

S. No.	Primer Name	Orientation	SNP	Primer sequence 5'-3'
1	*Araip.982E5*	FORWARD1	T	CCTTGCAGTATCATTCTCCGT
	Araip.982E5	REVERSE 1	G	CAAATCAAATCGAACCCTGC
	Araip.982E5	REVERSE 2	T	CAAATCAAATCGAACCCTGA
2	*Araip.9WJ7K*	FORWARD 1	A	AGTTGTTCCCTTGTGCTGTAAAA
	Araip.9WJ7K	FORWARD 2	T	AGTTGTTCCCTTGTGCTGTAAAT
	Araip.9WJ7K	REVERSE 1	T	GCCTATTTGCAAGACCACAAAA
3	*Araip.26ZIU*	FORWARD 1	T	GCATAGCATGGATTTTCATAGTGAT
	Araip.26ZIU	REVERSE 1	A	AGCAAATGGGCAAAAGATTA
	Araip.26ZIU	REVERSE 1	T	AGCAAATGGGCAAAAGATTA
4	*Araip.AD1F5*	FORWARD 1	T	AGAGAAGTCAAATTCCCCAAAGT
	Araip.AD1F5	FORWARD 2	C	AGAAGTCAAATTCCCCAAAGC
	Araip.AD1F5	REVERSE 1	C	CATCTGACTGGGACTATTTCTAATG
5	*Araip.J8WWD*	FORWARD 1	C	CAATTCACTTATCTCAGTGTCCTTC
	Araip.J8WWD	REVERSE 1	G	AGTGATGCTATGTGAGAATGTGAAC
	Araip.J8WWD	REVERSE 1	C	AGTGATGCTATGTGAGAATGTGAAG
6	*Aradu.96ASX*	FORWARD 1	T	CATCATAAACAGCATCTCTCAGACT
	Aradu.96ASX	REVERSE 1	A	TGTGAGGGATTGGCTCAACT
	Aradu.96ASX	REVERSE 2	T	GTGAGGGATTGGCTCAACA
7	*Aradu.S1EHV*	FORWARD 1	G	TGAGAGTTTAAGCTACTGTTGAGGG
	Aradu.S1EHV	FORWARD 2	T	TGAGAGTTTAAGCTACTGTTGAGGT
	Aradu.S1EHV	REVERSE 1	G	GGGGATGCTTTTGTTTATAATTCC
8	*Aradu.XF675*	FORWARD 1	G	GGGTATTTCGGTTTCCAGG
	Aradu.XF675	FORWARD 2	A	TGGGTATTTCGGTTTCCAGA
	Aradu.XF675	REVERSE 1	A	TTGAGCCGCAGATCCAACT
9	*Aradu.77ZLW*	FORWARD	G	CAGGTGCAGTGTTCAAACG
	Aradu.77ZLW	REVERSE 1	G	CCTCTGTGGTCAAATGGATAACTC
	Aradu.77ZLW	REVERSE 2	A	CCTCTGTGGTCAAATGGATAACTT
10	*Araip.1ZJ86*	FORWARD	A	AGAAACTGCAACAACCTCTTGA
	Araip.1ZJ86	REVERSE 1	A	CACACTCGCAAAACTAAAAGGAAT
	Araip.1ZJ86	REVERSE 2	T	CACTCGCAAAACTAAAAGGAAA
11	*Araip.XI9UR*	FORWARD	C	ACCACTTGTGGATCTTCTACTTGTC
	Araip.XI9UR	REVERSE 1	G	CCTGGTTAACAATGGCTTCC
	Araip.XI9UR	REVERSE 2	C	CCTGGTTAACAATGGCTTCG
12	*Araip.L4UC8*	FORWARD 1	G	TTCCAAACAAGGGTGGCAG
	Araip.L4UC8	FORWARD 2	A	TCCAAACAAGGGTGGCAA
	Araip.L4UC8	REVERSE	A	CACTATTGTCATATCTCGGCGT
13	*Aradu.03ENG*	FORWARD 1	A	AAGTAGGAAGTGGCTGAGTGGA
	Aradu.03ENG	FORWARD 2	T	AAGTAGGAAGTGGCTGAGTGGT
	Aradu.03ENG	REVERSE 1	A	TGTGTTAGAGAGGAAGCGGATT
	Aradu.03ENG	REVERSE 2	T	TGTGTTAGAGAGGAAGCGGATA
14	*Araip.G4DKZ*	FORWARD	A	GGTTGGATGATGAACCCGA
	Araip.G4DKZ	REVERSE 1	G	GCTTCAGGGTCATCAATTTCTC
	Araip.G4DKZ	REVERSE 2	A	GCTTCAGGGTCATCAATTTCTT
15	*Aradu.77ZLW*	FORWARD	G	CAGGTGCAGTGTTCAAACG
	Aradu.77ZLW	REVERSE 1	G	CCTCTGTGGTCAAATGGATAACTC

	Aradu.77ZLW	REVERSE 2	A	CCTCTGTGGTCAAATGGATAACTT
16	*Araip.26ZIU*	FORWARD	T	GCATAGCATGGATTTTCATAGTGAT
	Araip.26ZIU	REVERSE	T	AGCAAATGGGCAAAAGATTA
17	*Aradu.CVI2N*	FORWARD	C	TGTTGGATAATAATGCAGGTCATC
	Aradu.CVI2N	REVERSE 1	T	CTTTGATACATCAGGTGCCTTTA
	Aradu.CVI2N	REVERSE 2	C	TTTGATACATCAGGTGCCTTTG
18	*Aradu.618YE*	FORWARD 1	A	GATCCAAGCAAGCAAAGAAGA
	Aradu.618YE	FORWARD 2	C	ATCCAAGCAAGCAAAGAAGC
	Aradu.618YE	REVERSE	C	TTCAGATTCAGAGAGGTTCCG
19	*Aradu.S1EHV*	FORWARD 1	G	TGAGAGTTTAAGCTACTGTTGAGGG
	Aradu.S1EHV	FORWARD 2	T	TGAGAGTTTAAGCTACTGTTGAGGT
	Aradu.S1EHV	REVERSE	G	GGGGATGCTTTTGTTTATAATTCC
20	*Aradu.AGI8J*	FORWARD 1	G	TTGAATATATCCCAAGGCCAG
	Aradu.AGI8J	FORWARD 2	A	TTGAATATATCCCAAGGCCAA
	Aradu.AGI8J	REVERSE	G	CATGTTAACAATGCAATCAAGGAC
21	*Aradu.R479P*	FORWARD	C	GTATTACTCTCAGTCCTGTTCAAGC
	Aradu.R479P	REVERSE 1	T	CAAGAGCATTAGCCCTTTGAA
	Aradu.R479P	REVERSE 2	C	CAAGAGCATTAGCCCTTTGAG
22	*Araip.JH8FI*	FORWARD	G	CTGTCTTTGATTTGGAGTTCATTTG
	Araip.JH8FI	REVERSE 1	G	TGGGACAAGATTGTATATGGAAC
	Araip.JH8FI	REVERSE 2	C	TGGGACAAGATTGTATATGGAAG
23	*Araip.JH8FI*	FORWARD	C	CTGTCTTTGATTTGGAGTTCATTTC
	Araip.JH8FI	REVERSE 1	G	TGGGACAAGATTGTATATGGAAC
	Araip.JH8FI	REVERSE 2	C	TGGGACAAGATTGTATATGGAAG

Table S12: List of the differentially expressed genes between C-76 and Val-C genotypes with their expression values during full irrigation (FI) and deficit irrigation (DI) conditions

Gene Id	C-76-FI (FPKM)	C-76-DI (FPKM)	Val-C-FI (FPKM)	Val-C-DI (FPKM)	Annotation
Aradu.023N4	1.0	0.0	0.0	0.0	Transcription factor bHLH041
Aradu.025HX	16.1	8.4	0.0	5.5	Ribosomal S12 (Chloroplast)
Aradu.03ENG	3303.5	1935.4	118.1	348.3	Lipid Transfer
Aradu.0572C	0.0	0.0	1.1	0.0	Transcription Factor bHLH30
Aradu.05AEE	0.0	0.0	7.5	2.4	---Na---
Aradu.0HC1A	4.4	3.0	0.0	0.0	Clavaminate Synthase At3G21360
Aradu.0LH9P	391.9	408.5	43.5	107.3	Senescence-Associated
Aradu.0X4JL	8.5	4.4	0.0	3.0	Maturase K (Chloroplast)
Aradu.0XN7S	3.9	10.5	0.0	0.0	Epidermal Growth Factor Receptor Substrate 15
Aradu.130HL	15.4	9.8	0.0	8.1	U6 Snrna-Associated Sm Lsm6
Aradu.15REA	3.8	2.9	0.0	0.0	Isoprene Partial
Aradu.15WFY	3.2	2.7	0.0	0.0	Phosphatidylinositol:Ceramide Inositolphosphotransferase 1
Aradu.18J05	0.0	0.0	8.5	0.0	NAD-Dependent Malic Enzyme 59 Kda Mitochondrial
Aradu.1FT6I	81.8	23.7	1.4	28.6	Ribosomal S3 (Chloroplast)
Aradu.1H6YX	6.3	8.2	0.0	0.0	Sieve Element Occlusion C
Aradu.1W5FD	4.6	0.0	0.0	9.6	BON1-Associated 2
Aradu.24J3X	1.8	1.7	0.0	0.0	6B-Interacting 2
Aradu.2QX0T	16.5	9.8	1.7	9.2	Isoflavone 7-O-Methyltransferase
Aradu.3219N	2.9	3.9	0.0	1.1	Photosystem I P700 Apo A1
Aradu.32P5Q	13.0	7.6	0.0	0.0	Chloroplastic
Aradu.32RQ0	6.0	1.7	0.0	4.7	Ring-H2 Finger Atl3
Aradu.344E5	6.7	5.5	0.0	9.8	---Na---
Aradu.3TG3W	5.4	4.4	0.0	1.8	Partial (Chloroplast)
Aradu.3V07T	5.1	1.3	0.0	2.7	Phosphoglycerate Mutase Family
Aradu.3YG82	5.5	4.8	0.0	3.4	Nadh-Plastoquinone Oxidoreductase Subunit Partial (Chloroplast)
Aradu.413LK	2.0	1.5	0.0	1.1	Photosystem II D2 (Chloroplast)
Aradu.420FT	8.7	5.8	0.0	10.7	Predicted: Uncharacterized Protein Loc100814311

Aradu.42JCM	4.4	3.7	0.0	1.8	Hypothetical Chloroplast Rf19 (Chloroplast)
Aradu.46TFL	27.4	34.2	411.5	200.1	Dnaj Heat Shock Family
Aradu.49PAS	38.9	27.4	0.0	23.6	Isoflavone 2 -Hydroxylase
Aradu.4E997	0.0	1.0	125.1	50.5	MLP 43
Aradu.4V2X0	0.0	0.0	5.3	69.5	Thioredoxin H2
Aradu.4VP01	51.3	37.6	3.5	33.1	NADH-Plastoquinone Oxidoreductase Subunit I (Chloroplast)
Aradu.4X46H	2.5	2.6	0.0	0.0	Poly(Rc)-Binding 1
Aradu.53A5W	24.8	33.4	3.1	23.5	---Na---
Aradu.54RPC	48.7	11.6	6.0	30.8	---Na---
Aradu.572L7	1.7	2.2	15.2	3.5	Isoprene Synthase
Aradu.5A0JT	11.1	11.9	145.3	29.1	Probable Isoaspartyl Peptidase L-Asparaginase 2
Aradu.5H0AD	22.9	20.6	0.0	1.0	Lrr Receptorserine Threonine- Kinase Fls2
Aradu.61DK7	190.3	259.1	17.2	45.9	Cell Wall-Associated Hydrolase
Aradu.671CI	5.6	1.9	0.0	0.0	Probable Lrr Receptorserine Threonine- Kinase At3G47570
Aradu.6E94N	16.0	5.5	1.4	1.4	Bromodomain-Containing Factor 1Isoform X2
Aradu.6JX3E	322.6	462.1	39.5	237.2	Surfeit Locus
Aradu.6U69W	7.9	5.1	0.0	5.7	Nadh-Plastoquinone Oxidoreductase Subunit K (Chloroplast)
Aradu.73Q5I	4.6	4.2	0.0	0.0	Structural Maintenance Of Chromosomes 2-1
Aradu.75VJN	14.9	4.6	1.2	8.0	Disease Resistance (Tir-Nbs-Lrr Class)
Aradu.77SYB	374.5	226.5	33.6	231.4	Hypothetical_ (Chloroplast)
Aradu.78K64	18.0	1.2	0.0	9.3	Plastocyanindomain
Aradu.79NM3	1.1	1.3	21.3	2.1	40S Ribosomal Sa
Aradu.79WAC	13.7	9.0	0.0	6.9	Ribulose-1,5-Bisphosphate Carboxylase Oxygenase Large Partial (Chloroplast)
Aradu.7NI0J	0.0	0.0	2.3	0.0	Ulp1 Protease Carboxy-Terminal Domain
Aradu.7X3WY	1.9	2.9	15.4	2.8	Poly(Adp-Ribose) Glycohydrolase 1Isoform X1
Aradu.7ZK0R	0.0	0.0	1.4	0.0	Peroxidase Family
Aradu.80YRY	18.1	3.5	235.1	17.3	Flowering Locus T
Aradu.8101U	0.0	0.0	4.6	2.5	Bidirectional Sugar Transporter N3
Aradu.81UZ4	1.5	0.0	0.0	0.0	Pentatricopeptide Repeat-Containing Mitochondrial
Aradu.85352	48.5	43.5	5.9	35.2	Conserved
Aradu.8D21Y	9.3	7.1	0.0	6.5	---Na---

Aradu.8G53Y	11.7	15.3	1.4	11.7	Ribosomal L23 (Chloroplast)
Aradu.8G7CT	25.5	8.9	230.8	40.2	Wat1-Related At1G70260
Aradu.8GJ9B	113.3	21.4	11.2	62.6	---Na---
Aradu.8H8DD	15.3	14.6	0.0	5.4	Photosystem I P700 Apo A2 (Chloroplast)
Aradu.8I4EW	1.8	0.0	0.0	1.1	Periodic Tryptophan 1 Homolog
Aradu.8KH6E	2.8	0.0	0.0	1.7	U-Box Domain-Containing 21
Aradu.8Q8DX	7.0	6.5	0.0	0.0	E3 Ubiquitin- Ligase Rf298
Aradu.8WP5Z	15.2	10.7	1.2	9.1	Duf506 Family
Aradu.92LSD	62.2	52.3	2.8	3.3	Hypothetical Protein Phavu_003G055000G
Aradu.93Y3Y	24.5	23.0	0.0	0.0	---Na---
Aradu.94UA3	1.0	0.0	12.6	0.0	---Na---
Aradu.95FNB	3.5	3.0	0.0	2.1	NADH-Plastoquinone Oxidoreductase Subunit 5 (Chloroplast)
Aradu.97XXG	51.5	52.8	6.0	10.0	ATP Synthase Subunit Beta
Aradu.99525	3.2	2.3	0.0	1.7	Envelope Membrane (Chloroplast)
Aradu.99PKX	0.0	0.0	5.0	1.2	---Na---
Aradu.9AK3C	6.1	6.6	0.0	4.9	---Na---
Aradu.9FN01	22.1	22.2	0.0	16.0	Pre-Mrna-Splicing Factor Sf2
Aradu.A4MFG	32.3	49.6	2.8	18.1	Hypothetical_ (Chloroplast)
Aradu.AB4DD	41.6	42.4	3.7	20.6	Ribosomal S12 (Chloroplast)
Aradu.AF71L	22.9	16.4	1.5	13.5	ATP Synthase Cf1 Alpha Subunit (Chloroplast)
Aradu.AGI8J	0.0	0.0	24.2	0.0	Terpene Synthase Metal-Binding Domain
Aradu.AI0TY	15.9	14.0	0.0	6.8	Hypothetical Chloroplast Rf1 (Chloroplast)
Aradu.AYN79	103.9	100.4	3.6	39.6	Nad(P)-Binding Rossmann-Fold
Aradu.B2QV1	20.2	15.8	2.3	3.1	26S Proteasome Non-Atpase Regulatory Subunit 4
Aradu.B5X95	0.0	1.1	7.3	1.2	Probable Ccr4-Associated Factor 1 Homolog 11
Aradu.BLN97	0.0	0.0	4.1	1.5	Replication Factor C Subunit 1
Aradu.BW81J	27.5	26.3	3.4	16.5	Atp Synthase Cf1 Epsilon Subunit (Chloroplast)
Aradu.BX0IC	24.3	8.6	2.2	32.3	BON1-Associated 2
Aradu.CI98G	1.4	0.0	0.0	0.0	Lrr Receptorkinase Family
Aradu.CMH7M	26.9	33.8	0.0	0.0	Oxidoreductase Transition Metal Ion-Binding
Aradu.D7VM0	2.3	1.5	0.0	2.0	---Na---

Aradu.D8UNY	3.0	0.0	0.0	2.5	Peroxidase 47
Aradu.D91GM	1.6	2.6	0.0	0.0	Leucine-Rich Repeat Receptor Kinase
Aradu.DF5FL	1.7	1.3	0.0	0.0	Endo-1,4-Beta-Xylanase A
Aradu.DK48U	18.2	3.3	2.2	4.8	Peroxisome Biogenesis 6
Aradu.DV5XC	2.9	1.8	0.0	0.0	Zinc Finger Mym-Type 1
Aradu.DY203	3.4	3.8	0.0	1.9	NAD(P)H-Quinone Oxidoreductase Subunit H
Aradu.DZ6IG	0.0	0.0	3.4	0.0	Acid Phosphatase 1
Aradu.E0DEN	3.0	3.7	0.0	1.7	Hypothetical Chloroplast Rf2 (Chloroplast)
Aradu.EAG5M	1.0	1.0	0.0	0.0	Vacuolar-Sorting Receptor 1
Aradu.EF37G	9.1	4.3	0.0	0.0	Abscisic Acid 8 -Hydroxylase
Aradu.EGH8I	0.0	0.0	14.0	15.4	---Na---
Aradu.EU1QI	232.3	240.7	25.8	64.4	Rrna Intron-Encoded Homing Endonuclease
Aradu.F30S6	0.0	0.0	2.3	1.4	---Na---
Aradu.F5JK8	0.0	1.4	8.5	5.7	Cytochrome P450 71A1
Aradu.F6FA7	97.3	76.6	5.8	46.4	Ribosomal S7 (Chloroplast)
Aradu.F8AYK	0.0	0.0	12.4	10.7	Nsp-Interacting Kinase 3
Aradu.F8RAG	2.9	0.0	0.0	0.0	MYB-Related Myb4
Aradu.FJ2EW	54.9	61.6	6.3	15.0	Senescence-Associated
Aradu.G0NJW	5.0	11.1	72.1	77.5	Flowering Locus T
Aradu.G4NCZ	7.7	5.4	0.0	3.5	Racgtp-Binding Rac13
Aradu.GD6AN	2.0	0.0	0.0	0.0	Calmodulin Binding -
Aradu.GFT6J	5.9	0.0	0.0	1.2	Tify 5A
Aradu.GG3LG	3.9	3.2	0.0	0.0	AP2Ethylene-Responsive Transcription Factor Ant
Aradu.GWN14	0.0	0.0	3.4	2.0	Methylecgonone Reductase
Aradu.GXJ7L	4.2	3.5	0.0	5.4	Pollen Ole E 1 Allergen And Extensin Family
Aradu.H1E2F	11.1	13.3	1.1	4.7	Cytochrome B Reductase 1
Aradu.H33XQ	0.0	1.4	5.4	0.0	Far1-Related Sequence 5
Aradu.H563E	0.0	0.0	7.2	7.1	Uncharacterized Protein Loc100500574
Aradu.HR9H4	0.0	0.0	1.6	3.6	Udp-Glycosyltransferase 83A1
Aradu.I2J7R	0.0	1.8	6.8	55.8	Lob Domain-Containing 4
Aradu.I5C3V	0.0	1.4	10.4	1.9	Poly(Adp-Ribose) Glycohydrolase 1Isoform X2

Aradu.I8HGC	2.8	3.5	47.8	33.4	Hypothetical Protein Glysoja_043054
Aradu.J6XSM	0.0	0.0	8.1	2.5	Chalcone Synthase
Aradu.JEF8K	0.0	0.0	11.6	0.0	Casp Rcom_1174750
Aradu.JIY90	18.2	9.3	1.2	7.5	NADH
Aradu.JJU8Z	18.2	11.7	2.0	6.6	Receptor Kinase
Aradu.JM7KB	1.1	0.0	0.0	0.0	Probable Glycerol-3-Phosphate Acyltransferase 2
Aradu.K26ER	12.3	5.6	0.0	0.0	---Na---
Aradu.K3I53	0.0	0.0	3.5	0.0	NRT1 PTR Family
Aradu.K3VD2	8.2	11.0	0.0	4.2	Ribosomal S3 (Chloroplast)
Aradu.K88X4	10.0	16.3	1.1	10.7	Photosytem I Subunit VII
Aradu.KG41H	27.0	16.8	2.9	8.7	WRKY Transcription Factor 30
Aradu.L4Z5B	1.4	1.1	19.6	15.5	---Na---
Aradu.L67UJ	5.3	3.8	0.0	0.0	HMG (High Mobility Group) Box
Aradu.LIH6D	0.0	0.0	4.8	0.0	39S Ribosomal Mitochondrial
Aradu.M3CK1	10.1	2.5	1.2	0.0	Homogentisate Phytyltransferase Chloroplasticisoform X2
Aradu.M3XI9	1.1	1.1	0.0	1.7	Gdsllipase Acylhydrolase
Aradu.M9E5N	26.0	28.2	2.8	14.5	Cell Wall-Associated Partial
Aradu.MSL3N	2.6	1.5	0.0	1.1	Myb-Related Myb4
Aradu.N5Y0T	6.3	3.5	0.0	2.2	Maturase (Chloroplast)
Aradu.NE93Q	0.0	1.5	5.1	1.0	Plasma Membrane-Associated Cation-Binding 1
Aradu.NK24P	0.0	0.0	3.4	1.8	Ethylene-Responsive Transcription Factor Abr1Isoform X1
Aradu.NP81U	2.6	1.5	0.0	0.0	Metacaspase-1
Aradu.NTZ01	4.6	9.8	0.0	2.2	Jq0280 Hypothetical 12K (Trna Intron) - Rice Chloroplast
Aradu.P47M2	0.0	0.0	12.4	6.0	Pathogenesis-Related 1
Aradu.PEA62	8.0	0.0	0.0	4.3	Replication Factor-A Carboxy-Terminal Domain
Aradu.PI7XA	4.0	2.8	40.4	2.5	---Na---
Aradu.PRD5G	0.0	0.0	0.0	1.8	ABC Transporter C Family Member 10
Aradu.PU456	0.0	1.9	24.5	4.7	Sesquiterpene Synthase
Aradu.QYF40	5.4	6.5	0.0	2.2	Rna Polymerase Beta Subunit (Chloroplast)
Aradu.R7QS7	7.3	6.0	0.0	3.5	Nadh-Plastoquinone Oxidoreductase Subunit 7 (Chloroplast)
Aradu.RA346	13.7	13.4	1.3	1.2	L-Ala-D L-Glu Epimerase

Aradu.RA7PE	2.3	4.3	18.8	10.7	O-Acyltransferase Wsd1
Aradu.RU8HS	5.0	0.0	0.0	3.6	Late Embryogenesis Abundant
Aradu.S1EHV	20.2	18.4	0.0	24.5	Dna Mismatch Repair Msh6
Aradu.S4CJ2	4.4	4.4	49.8	32.8	MADS-Box Soc1
Aradu.S88B1	587.2	582.6	50.9	475.0	Cytochrome B Reductase 1
Aradu.SL2ND	7.5	3.1	0.0	2.1	Extra-Large Guanine Nucleotide-Binding 1
Aradu.SMZ6S	6.5	4.2	0.0	0.0	Bromodomain-Containing Factor 1Isoform X2
Aradu.STH91	0.0	0.0	4.6	7.0	Dna Rna Polymerases Superfamily Isoform 2
Aradu.T2SCC	24.6	22.8	2.5	24.7	Serine Threonine- Kinase
Aradu.T5GD5	43.8	40.8	5.1	29.1	Photosystem Ii D2
Aradu.T98DX	0.0	0.0	3.5	2.7	---Na---
Aradu.TDC45	7.6	7.2	0.0	0.0	Clavaminate Synthase At3G21360
Aradu.U1FLK	4.1	3.1	0.0	0.0	Nuclease Harbi1
Aradu.U5WMC	2.0	2.2	0.0	1.1	Nadh Dehydrogenase Subunit 2 (Chloroplast)
Aradu.U8TGW	3.3	0.0	0.0	2.3	Lysm Domain Receptorkinase 4
Aradu.U9FPR	22.8	16.8	0.0	11.0	Ribosomal S15 (Chloroplast)
Aradu.UGD4H	213.2	211.5	23.9	69.0	Senescence-Associated Partial
Aradu.V0PXB	6.9	2.9	0.0	5.3	Coatomer Subunit Beta -2Isoform X1
Aradu.V72Z0	1.4	1.8	0.0	0.0	Predicted: Uncharacterized Protein Loc105043294 Isoform X1
Aradu.V83WY	27.4	19.3	2.3	13.2	Hypothetical Chloroplast Rf1 (Chloroplast)
Aradu.W09PA	42.0	42.3	4.1	20.2	Ribulose 1,5-Bisphosphate Carboxylase Oxygenase Large Subunit (Chloroplast)
Aradu.WBM86	4.8	2.6	0.0	0.0	Oxidoreductase Nad-Binding Rossmann Fold
Aradu.WL7RN	16.8	18.7	0.0	0.0	Predicted: Uncharacterized Protein Loc104211086, Partial
Aradu.WQX7T	21.1	22.0	2.0	15.6	Cytochrome B Reductase 1
Aradu.WX3Q6	5.4	4.1	0.0	3.8	ATP Synthase Cf1 Alpha Subunit (Chloroplast)
Aradu.WYR9Z	166.8	70.7	4.8	79.6	ATP Synthase Cf1 Alpha Subunit (Chloroplast)
Aradu.XF675	82.7	48.7	5.7	46.2	ATPase Alpha Partial
Aradu.XIP04	1.3	0.0	0.0	0.0	Probable Lrr Receptorserine Threonine- Kinase At3G47570
Aradu.XJY61	2.5	8.4	33.0	23.8	---Na---
Aradu.Y02KH	3.6	2.4	56.3	7.7	Mads-Box Partial
Aradu.Y3QBI	4.7	0.0	0.0	0.0	Alpha-Dioxygenase 1

Aradu.Y6LUX	3.1	2.2	41.3	35.7	Late Embryogenesis Abundant Group 3
Aradu.YD5BQ	1.2	0.0	0.0	1.2	Indole-3-Acetate O-Methyltransferase 1
Aradu.YH2WE	35.0	13.9	1.5	11.4	Ribosomal S12 (Chloroplast)
Aradu.YJB40	1.7	1.7	0.0	0.0	Photosystem I P700 Chlorophyll A Apo A1 (Chloroplast)
Aradu.YY5C4	1.5	0.0	0.0	0.0	Auxin-Induced 5Ng4
Aradu.Z2UCJ	0.0	0.0	8.0	11.7	Serine Threonine- Kinase Tricorner Isoform X2
Aradu.Z6652	0.0	0.0	6.0	6.3	GDSl Esterase Lipase Exl3
Aradu.ZD91B	12.0	14.0	0.0	5.7	---Na---
Aradu.ZD9KZ	0.0	0.0	4.2	19.2	Expansin Alpha ,Expa1
Aradu.ZKQ4D	2.9	2.1	0.0	1.4	ATP Synthase Cf0 Subunit Iv (Chloroplast)
Aradu.ZXE5G	3.9	3.2	0.0	0.0	Pyruvate Dehydrogenase E1 Component Subunit Mitochondrial

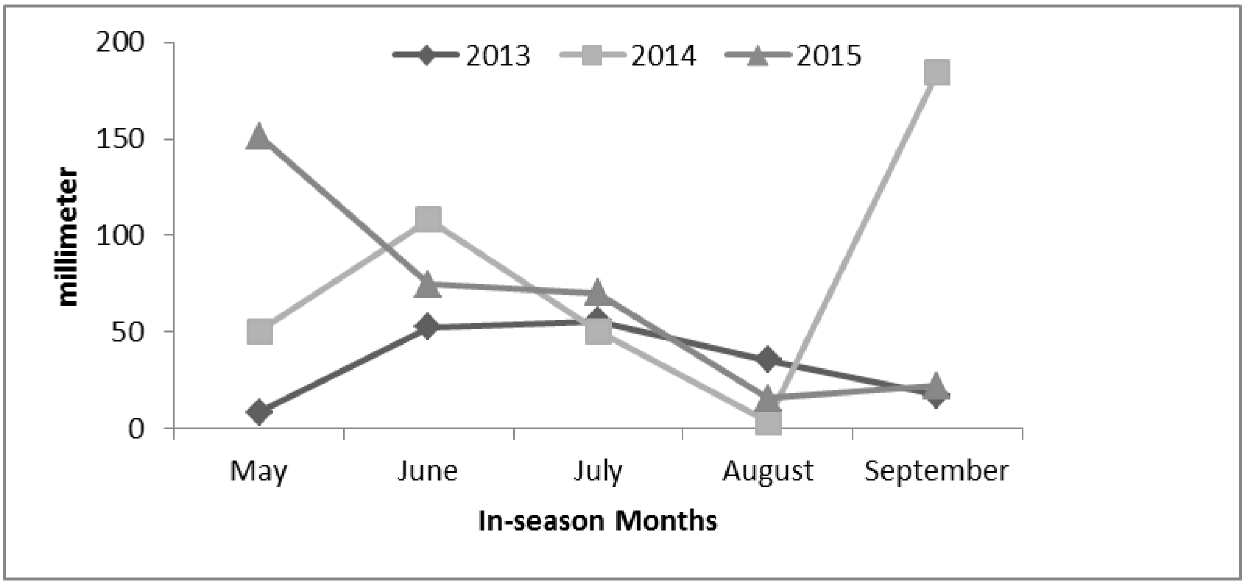

Supplementary Figure 1: Mean precipitation (mm) obtained during peanut production months from May to September. The highest amount of precipitation was recorded in 2014 (38 cm), followed by 2015 (34 cm) and 2013 (17 cm).

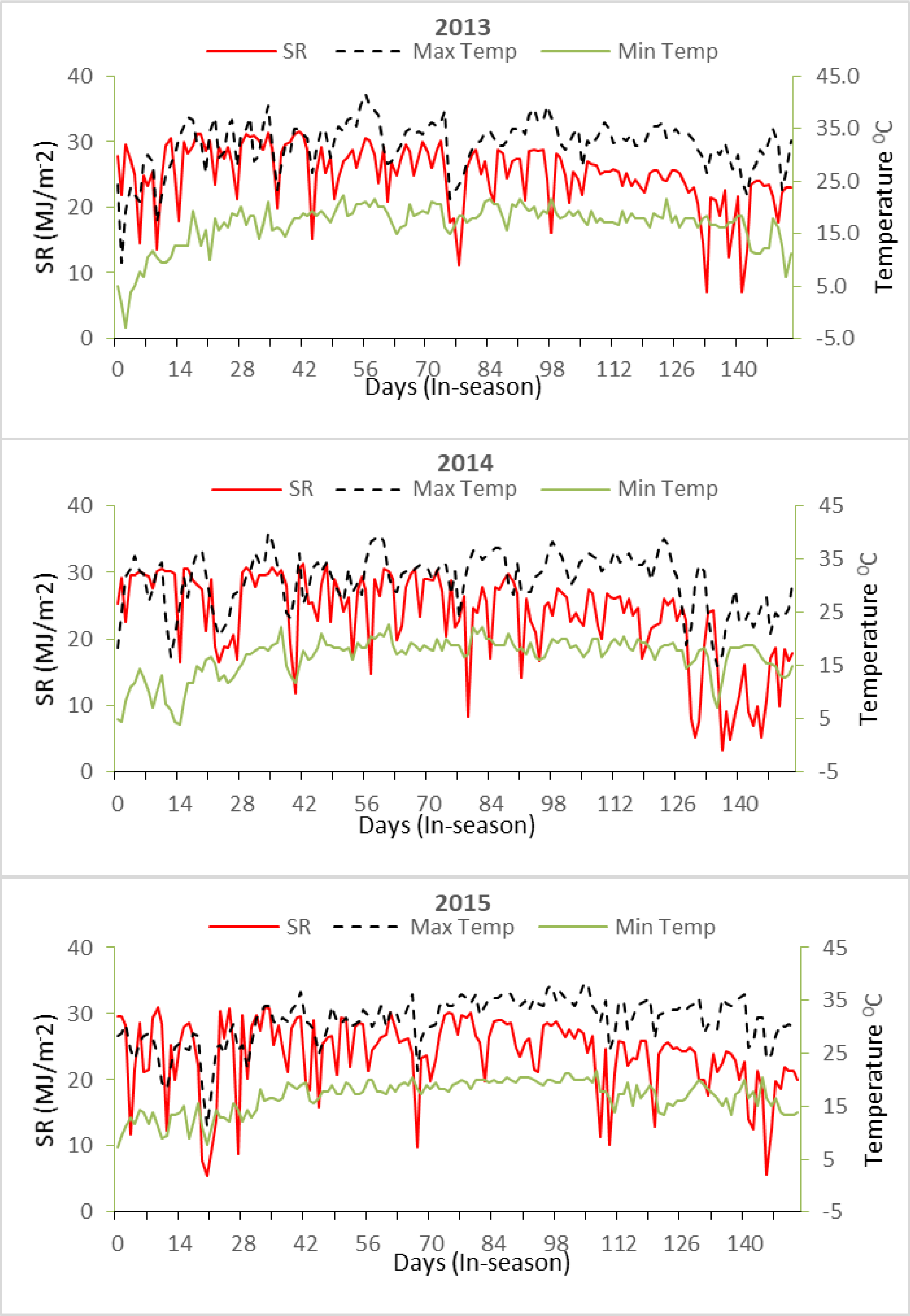

Supplementary Figure 2: Mean solar radiation (SR), maximum air temperature and minimum air temperature during the in-season months of peanut cultivation from May to September of years 2013, 2014 and 2015.

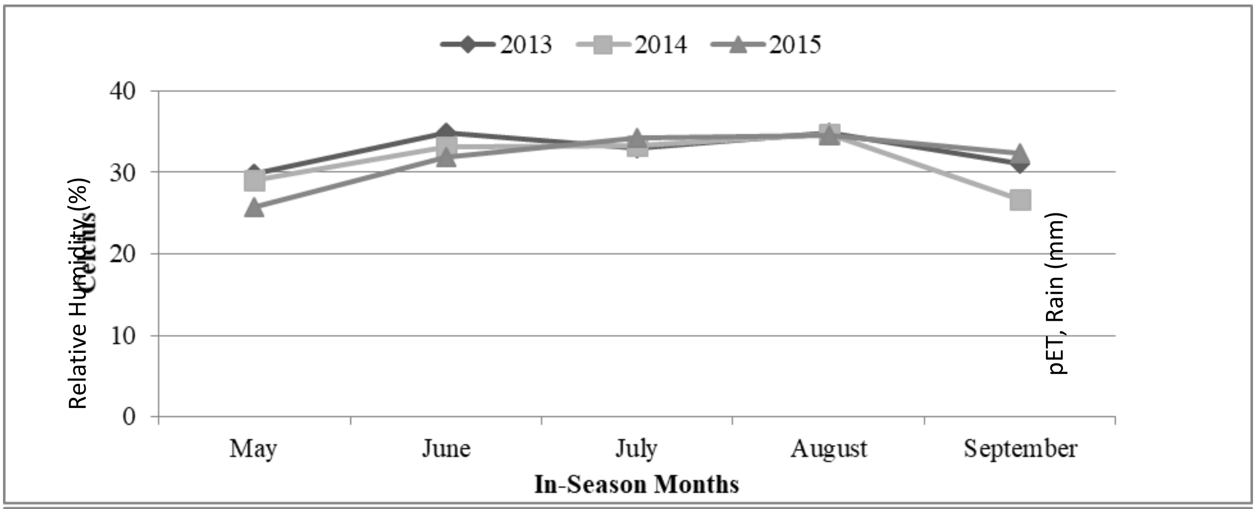

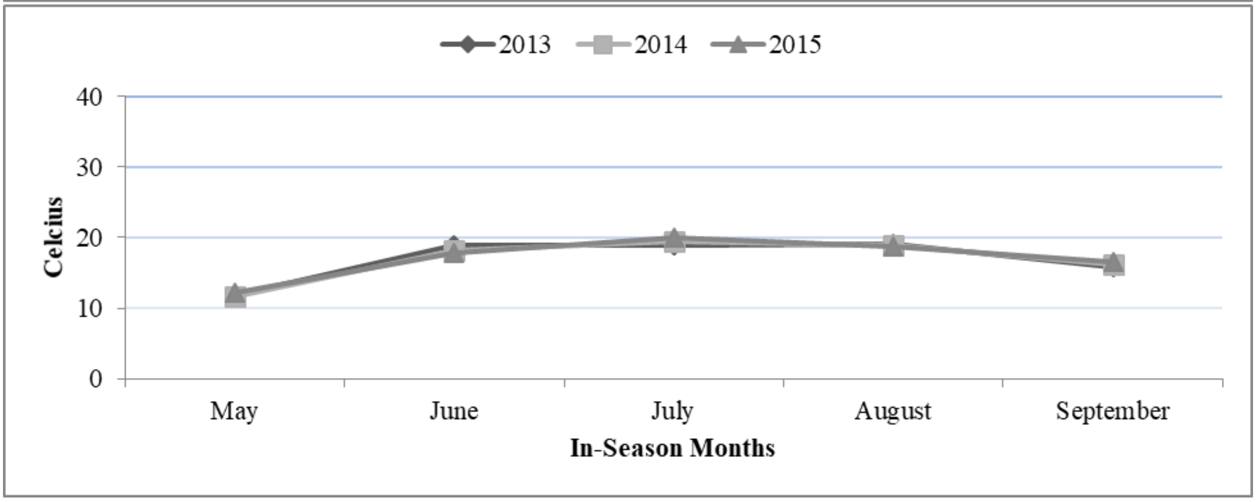

Supplementary Figure 3: Mean maximum and minimum temperature during the in-season months of peanut production from May to September.

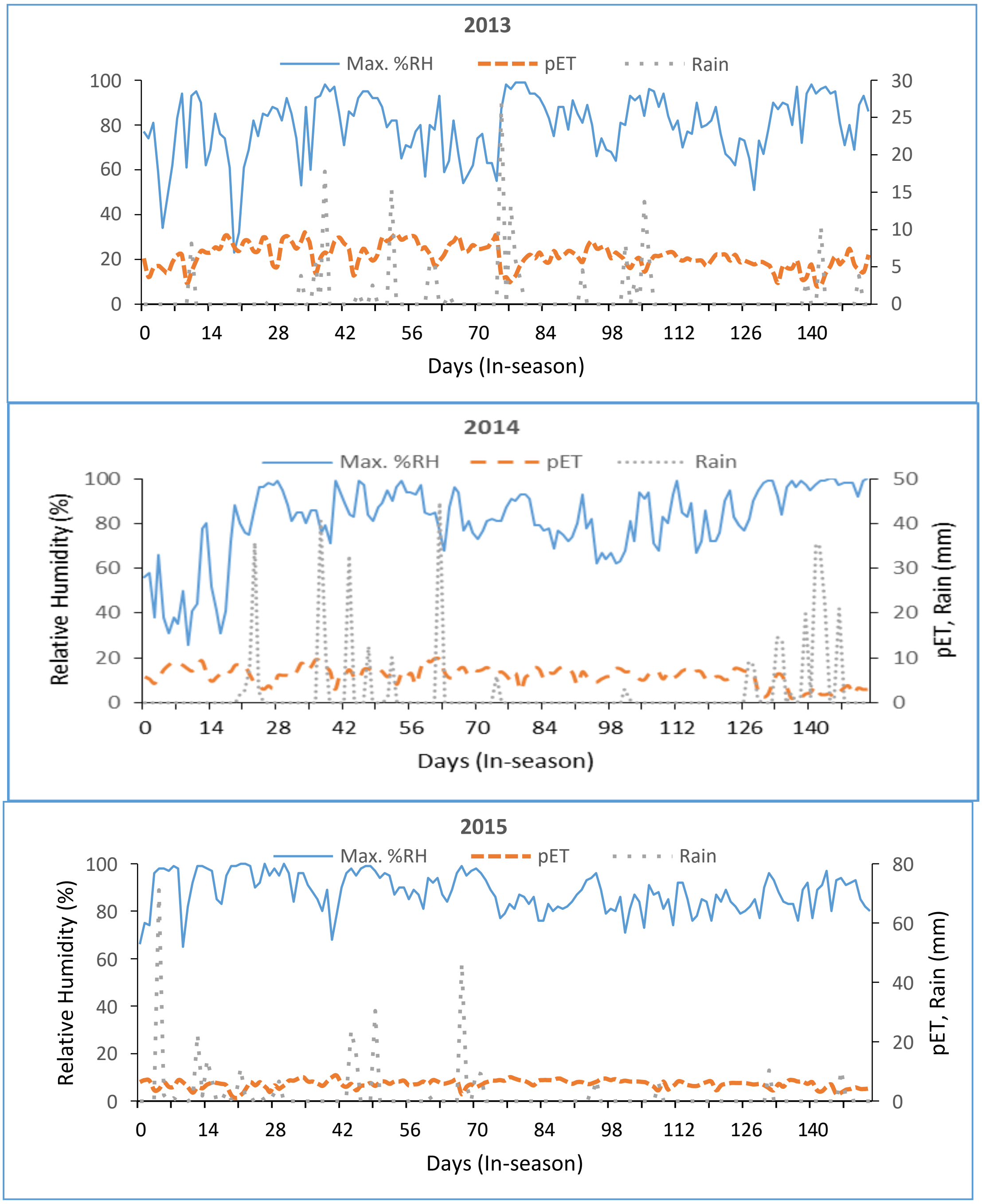

Supplementary Figure 4: Mean relative humidity, potential evapotranspiration (pET) and rain during the in-season months of peanut cultivation from May to September of years 2013, 2014 and 2015: Relative humidity was highest in 2015, followed by 2014 and 2013 and evapotranspiration rate was highest in 2013, followed by 2014 and 2015.

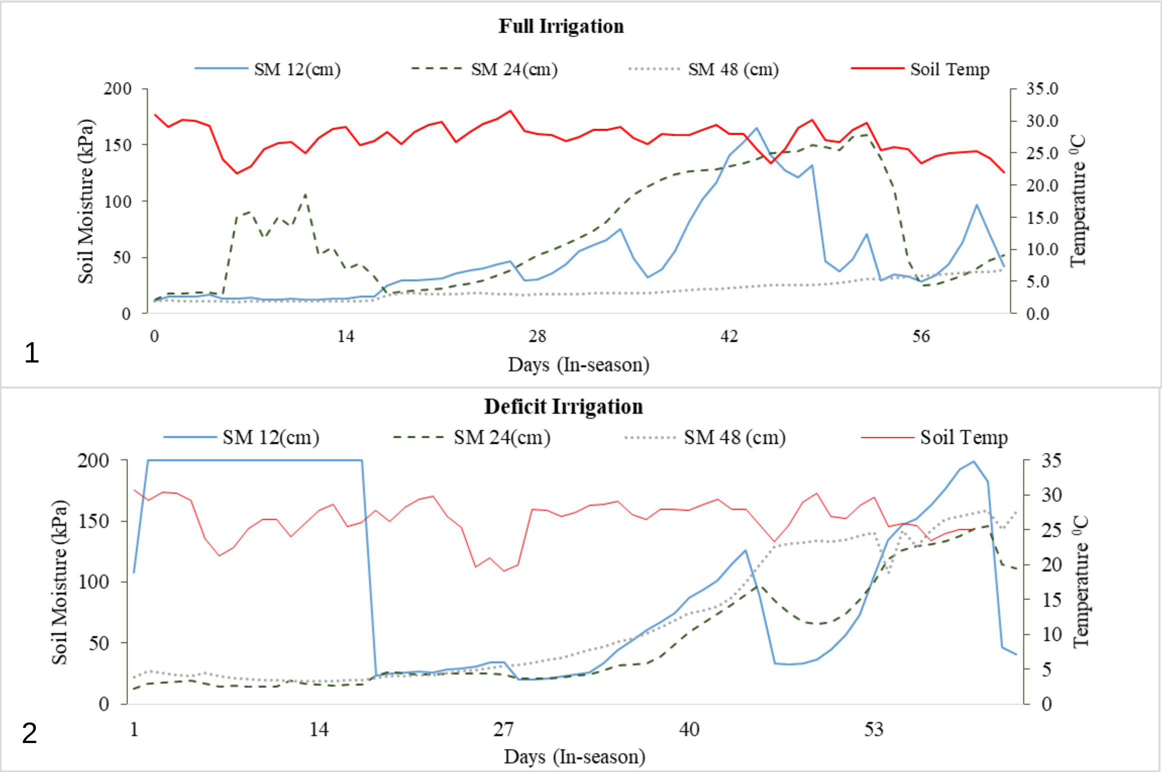

Supplementary Figure 5: Soil moisture was measured at 12, 24, and 48 cm below the soil surface while soil temperature was measured at 4 cm below the soil surface for the two different types of irrigation methods- a) Full irrigation b) Deficit irrigation.

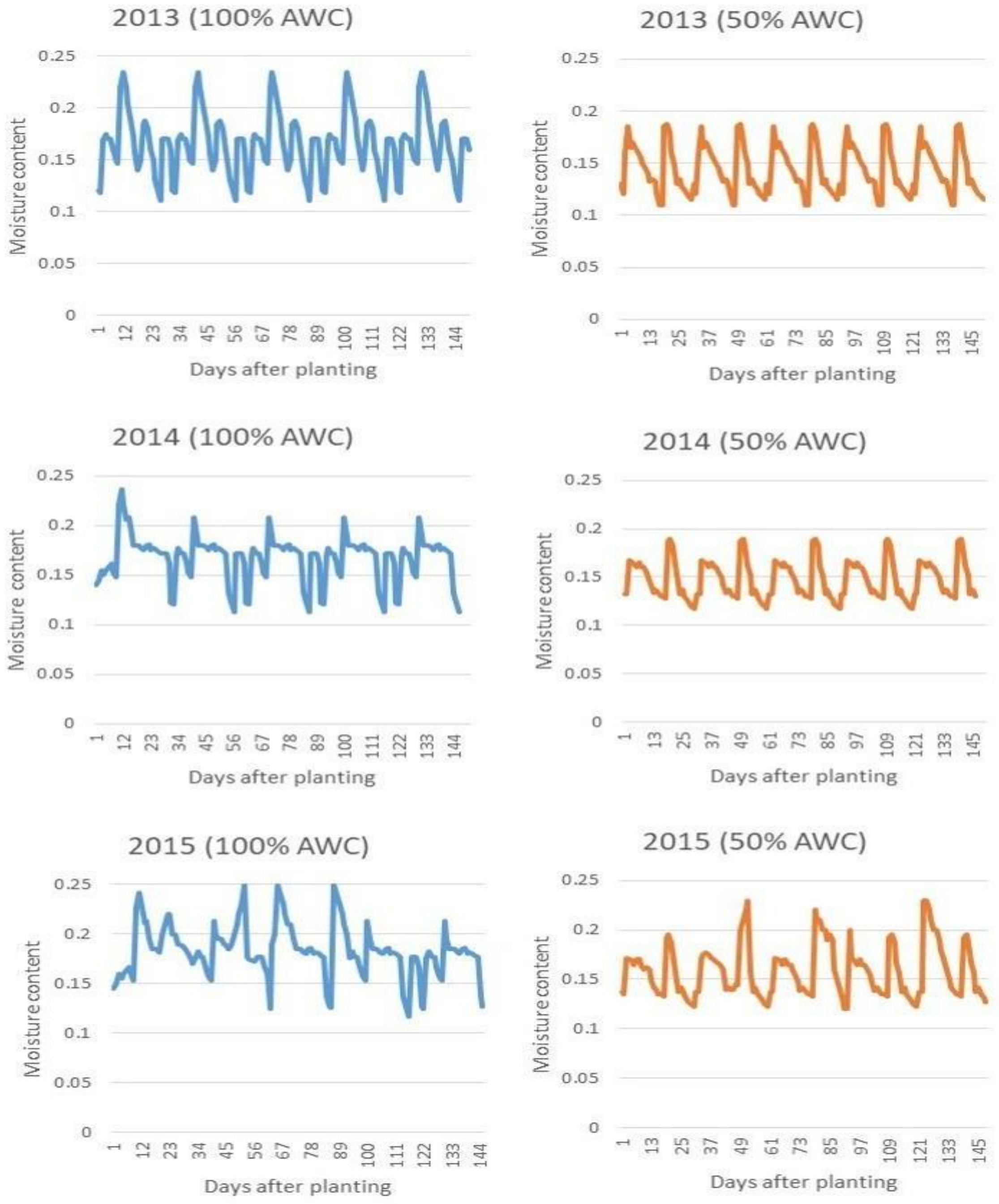

Supplementary Figure 6: Average soil moisture content (0-30 cm depth) during the growing season.

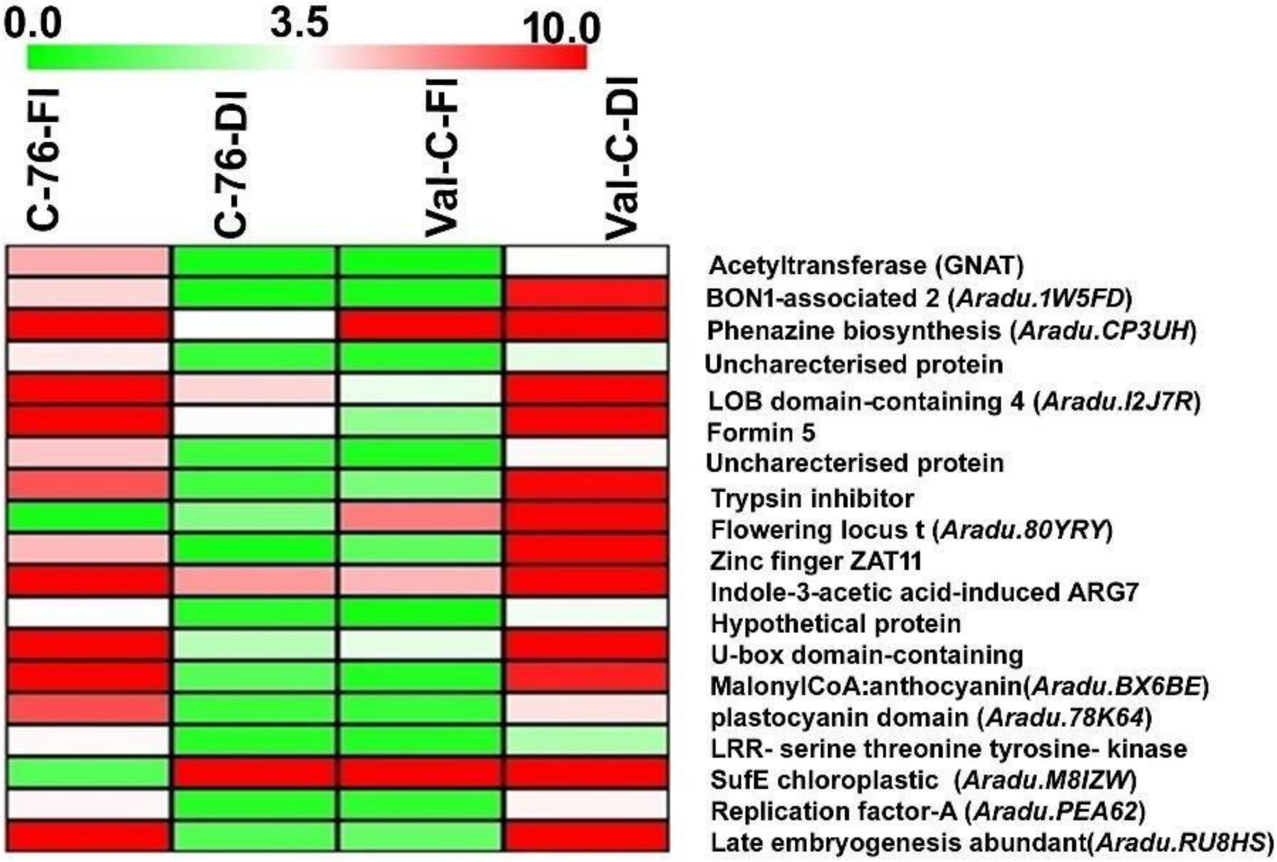

Supplementary Figure 7: Heat map representing the expression of common differentially expressed genes on A genome in C-76 and Val-C genotypes during full irrigation and deficit irrigation (DI) conditions.

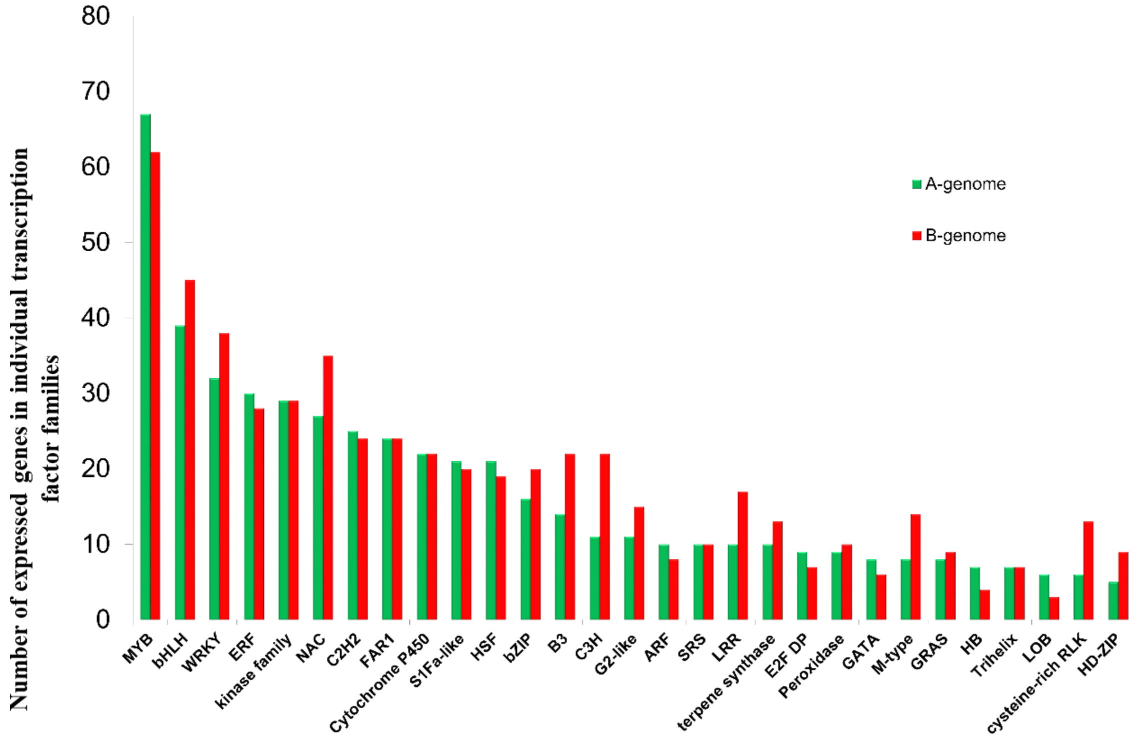

Supplementary Figure 8: Most abundant transcription factor (TF) families encoding genes under deficit irrigation (DI) stress in peanut.

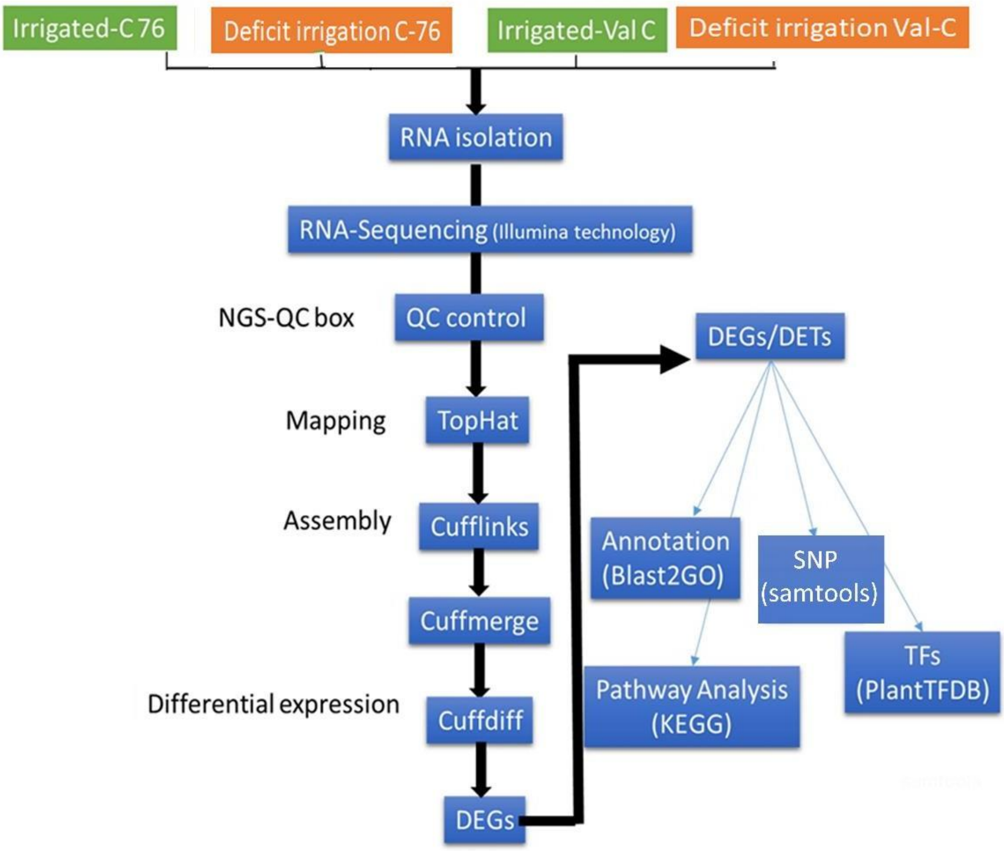

Supplementary Figure 9: Overall workflow of the peanut drought stress transcriptomic study.

Supplementary information on methodology

Crop management methodology

Prior to peanut cultivation, wheat was cultivated in the field each year. Fields were tilled and ploughed before planting peanut seeds. Plots were marked and seeds were planted on May 13th in 2013, May 14th in 2014 and May 12th in 2015. Each plot was about 4.5 m long, having rows in which seeds were planted 5 cm apart; in 2 to 3 m long alleys to separate each germplasm and 100 cm between rows. Peanut seeds were planted using a two row mechanical planter (John Deere MaxEmerge planter), each planter had a seed metering cone. Each year, the treatments were separated from 200 to 500 m in space. The space between the treatments ensured that excess water did not reach the deficit irrigation field. Each treatment was separated by two to four border plants from the farmer's field crops, so as to minimize interactions. The field was irrigated through a central pivot irrigation system.

The field was fertilized prior to planting and thoroughly disked with a mouldboard plough. During plant growth, fertilizers (67 kg N^{-1} and 40 kg DAP^{-1}) were applied both pre-plant and mid-season around 60- 65 days after planting (DAP). Each year after 45, 60 and 90 DAP, liquid micronutrients were applied through the irrigation system. Diseases, insects and weeds were periodically controlled or prevented (on need basis) using labelled products each year.

Both treatments received the same crop management practices except irrigation levels. The full-irrigation field received 100% AWC while the deficit irrigation field received about 50% AWC. The full irrigation plot received 4.45 cm of water every 10 days while the deficit irrigation plot received 4.45 cm of water every 20 days in all three years. The irrigation cycle was every 10 days, for half-irrigation the pivot sprinklers watering the plots were shut off every alternating irrigation cycle.

Data collection

Weather and meteorological conditions

Precipitation and temperature were recorded during the in-season month of peanut production. The in-season months of peanut production in West Texas are usually between May to September. Data were collected from the archives of Texas Tech University, weather station (westtexasmesonet, 2016). Daily meteorological data was recorded using an automated weather station from West Texas Mesonet located in Brownfield, Texas. Daily weather data

collected were used in determining the total water received (rainfall+irrigation) as a percentage of the ETo (TWRPET). Reference ETo was calculated using the Peanman-Monteith equation (FAO-56 method) using crop coefficient values for peanuts (Allen et al., 1998; Monteith 1965). The daily ETc was cumulated and when it equals the management allowable depletion (MAD), it was divided by the system efficiency to get the gross irrigation rate which was applied. We used center pivot irrigation with 85% application efficiency. The TWRPET was calculated at 0-45 days after planting (DAP), 45-90 DAP, and 90-harvest to determine how TWRPET might affect critical periods of phenological development because inherent variability in different sensitivities to water-deficit stress in peanut (Reddy, Reddy and Anbumozhi 2003). The 45-90 DAP encompasses development period when pod yield reduction is most sensitive to water stress, whereas > 90 DAP is relatively insensitive (Patel and Golakiya 1988). This approach aids in determining the relative potential impact of TWRPET on peanut yields; if TWRPET is low during developmental periods when stress sensitivity is high, the impact on yield is predicted to be high.

The mean, maximum and minimum temperature of each month were obtained from individual days throughout the period of crop cultivation. The mean precipitation for each month was also obtained in the same way.

Soil moisture and temperature

Watermark moisture probes (Irrometer, Riverside, CA) were placed at 12, 24 and 48 cm below the soil surface. One temperature probe was placed at 6 cm below the soil surface. All the probes were connected to a data logger. The data logger was either a WatchDog – Model 400 or WatchDog 1000 series (Spectrum Technologies, Inc.). Data from the data logger was obtained using the Spec 9 Pro software (Spectrum Technologies, Inc.). Measurements were recorded throughout the season. Soil moisture was recorded in kilo Pascal (kPa) and the reading ranged from 0 to 200; 0 being very wet and 200 being very dry.

References

54. Dwivedi, S. L., Nigam, S. N., Reddy, D. V. R., Ranga Rao, G. V. & Reddy, A. S. Registration of ICGV 86388 peanut germplasm. *Crop Sci.* **36**, 1423 (1996).
55. Nigam, S. N., Rao, M. J. V., Upadhyaya, H. D., Rao, Y. L. C. & Reddy, N. S. Registration of an early-maturing peanut germplasm ICGV 86015. *Crop Sci.* **35**, 1718–1719 (1995).
56. Smith, O. D., Simpson, C. E., Grichar, W. J. & Melouk, H. A. Registration of Tamspan 90 peanut. *Crop Sci.* **31**, 1711 (1991).
57. Okello, D. K., Deom, C. M., Puppala, N., Monyo, E. & Bravo-Ureta, B. Registration of Serenut 5R. *J Plant Regist.* **10(2)**, 115–118 (2016).
58. Okello, D. K., Deom, C. M., Puppala, N., Monyo, E. & Bravo-Ureta, B. Registration of Serenut 6T. *J Plant Regist.* **12**, 43–47 (2018).
59. Holbrook, C. C. & Dong, W. Development and evaluation of a mini core collection f or the U.S. peanut germplasm collection. *Crop Sci.* **45**, 1540–1544 (2005).
60. Nigam, S. N., Dwivedi, S. L., Rao, Y. L. C. & Gibbons, R. W. Registration of ICGV 87141 peanut. *Crop Sci.* **31**, 1096 (1991).
61. Hsi, D. C. H. Registration of New Mexico Valencia C peanut. *Crop Sci*. **20**, 113–114 (1980).
62. Rathnakumar, A. L., Singh, R., Parmar, D. L., Misra, J. B. Groundnut a crop profile and compendium of notified varieties of India (2013).
